# Drought and disease trigger novel interactions among top predators

**DOI:** 10.1038/s41586-026-11194-z

**Published:** 2026-10-21

**Authors:** Fabrizio Sergio, Haruki Natsukawa, Alessandro Tanferna, Javier Chicano, Francisco Carro, Ramón C. Soriguer, Fernando Hiraldo, Julio Blas

**Affiliations:** 1 Department of Conservation Biology and Global Change, Estación Biológica de Doñana - CSIC, Seville, Spain; 2 Faculty of Agriculture, Niigata University, Niigata, Japan

**Keywords:** Climate-change ecology, Climate-change impacts

## Abstract

Interactions among species are increasingly recognized as key determinants of climate change impacts on ecosystems^1–5^. However, we know little about how climatic perturbations affect interactions among top vertebrate predators. Here we show that, when an emergent disease caused the decline of shared prey (rabbits), after decades of neutral co-existence a mammalian carnivore (lynx) started to prey heavily on a top predatory raptor (kites). These predation bouts occurred exclusively under drought conditions and when lynx found rabbit remains under the raptor nests, consistent with the removal of a potential competitor. Drought-induced lynx predation depressed lifetime reproductive success and triggered territory abandonment and dispersal by the raptor, suggestive of a new arms race among predators. A reduction in raptor abundance was associated with an increase in an insectivorous bird (a small owl), showing that the new interaction had the potential to reach lower trophic levels. These results underline the importance of diet flexibility and mobility as important mediators of resilience to stressors. In the future, synergistic effects of multiple anthropogenic stressors may reshape the interactions and ecological roles of many apex predators, with potential cascading consequences for whole ecosystems. A key to forecasting the future impact of global change may reside in understanding how behavioural flexibility and interacting keystone species respond to interacting shocks.

## Abstract (web summary)

Climate-driven drought and prey decline triggered lynx predation on raptors, reducing raptor fitness and abundance and producing cascading effects across lower trophic levels.

## Main

Species interactions, such as pollination or herbivory, are increasingly recognized as key drivers of climate impacts on biodiversity^1–5^. Climate change can profoundly alter biotic interactions—which in turn can reshape its impact—thus constraining species distribution^6,7^, imposing population declines^8^, driving extinctions^9–11^ and modifying food web structure, function and stability^12,13^. Understanding the relation between climate and biotic interactions is therefore considered an essential prerequisite for anticipating the ecosystem effects of climatic perturbations^2,3^. However, despite many recent advances for certain taxa and ecosystems^14–16^, we have scarce knowledge of the interplay between climate and several key interactions.

From a broader point of view, species interactions are likely to mediate a wide range of multiple interacting stressors, such as climate extremes, habitat degradation, environmental contamination, overhunting by humans or introduction of non-native species and pathogens^17,18^. Animals facing such stressors can either succumb (for example, decline to extinction^19^) or enact coping strategies that allow them to resist the stressor impact^20,21^. Resistance is usually attained through phenotypic plasticity and altered use of resources, such as food or habitats^22,23^, which may change or broaden the realized niche of a species. Such niche-rewiring has produced some recent, striking examples of species using new, unexpected foods, with positive consequences in terms of their abundance and resilience^23–26^. In turn, these niche alterations are likely to reshape the network of ecological interactions in which a species is embedded, with potential consequences for its supporting community. This will be particularly expected for species already involved in large networks of key interactions, such as top predators. Thus, there is a need for studies that examine how the response to multiple stressors by keystone predators repercusses on their interactions with other predators, with potential indirect consequences for lower-trophic level species.

One common interaction among predators is intraguild predation, which is the killing and consumption of species that compete for the same resource^27^. Intraguild predation is considered ubiquitous in food webs, widespread in guilds of top vertebrate predators^28,29^, and a common driver of population dynamics and community structure^30–32^. Intraguild predation is an ideal candidate for research on the interface between climate and biotic interactions, because it integrates competition and predation, both of which are affected by climate change and in turn modulate its impact^3–5,7,11^. However, knowledge on the interplay between climate change and intraguild predation is based almost exclusively on warming experiments on invertebrates^13,33,34^, with little research on vertebrate predators. This is despite two recent studies showing that climate change will probably increase the encounter rates among intraguild predation killers and victims^35,36^. Such a lack of information is particularly striking given that top predatory vertebrates: receive a large amount of management and research; are highly interactive species; can structure whole food webs through top-down forcing; are especially sensitive to climate change; and are considered ideal biotic multipliers, capable of amplifying the impact of climate change on their communities^32,37–39^.

Another less studied form of intraguild predation is represented by killings among taxonomically distant predators, such as mammalian carnivores and birds of prey. Such cross-taxonomic intraguild predation has proven difficult to investigate, probably due to the extreme difficulty of detection of rare events involving distantly related taxa typically studied by independent research specialists^40^. As such, research has been devoted to the sole description of individual casualties^41–43^. Knowledge is limited to a handful of studies that quantified the predation rate, or tested its impact on the victim abundance or behaviour^44–46^. The relation between cross-taxonomic intraguild predation and climate change remains uncertain.

**Fig. 1:**
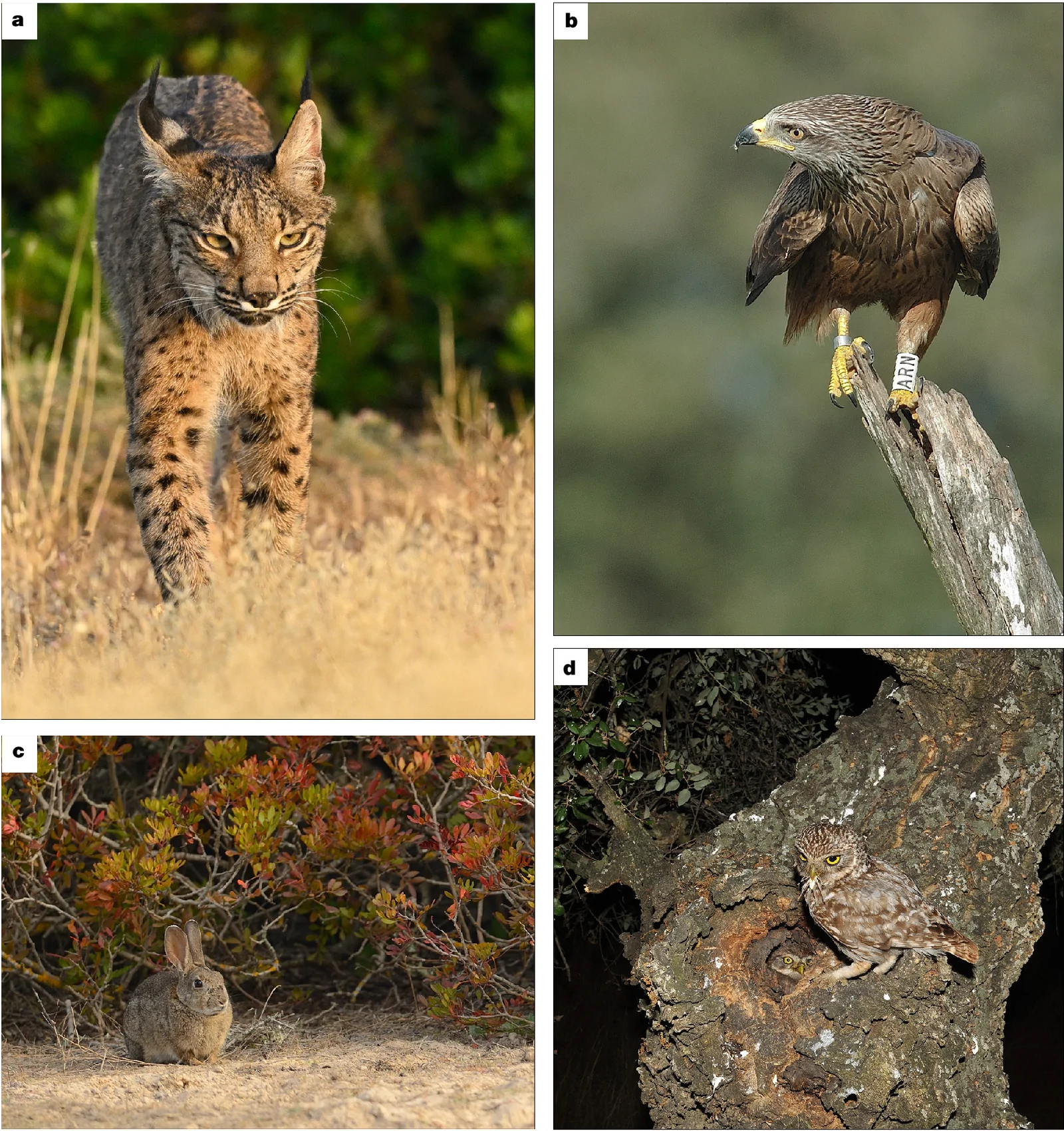
Model species composing the intraguild predation module of the study system. **a**–**d**, A mammalian carnivore (the Iberian lynx, **a**) depredates a diurnal raptor (the black kite, **b**) that competes for the same main prey (the Eurasian rabbit, **c**), with potential cascading consequences for lower-trophic-level species such as the insectivorous little owl (here delivering a typical grasshopper prey to its cavity nest in a cork oak, **d**). Lynx predation on a kite population, subject to long-term monitoring of marked individuals of known age (**b**), provided a unique opportunity to examine the fitness consequences and individual counter-strategies operated by the raptor in response to the new carnivore threat induced by climate change. Photo credit: F. Sergio.

Here we study an intraguild predation module composed of a mammalian carnivore (the Iberian lynx *Lynx pardinus*) and a raptorial bird (the black kite *Milvus migrans*) that share the same main prey (the European rabbit *Oryctolagus cuniculus*) (Fig. 1). These kinds of community modules are considered ideal study systems for gaining the necessary mechanistic understanding of interactions that are simultaneously vulnerable to climate and key determinants of community function and structure—an essential prerequisite for predicting community responses to climate change^2^. The Iberian lynx (hereafter lynx) is a medium-sized solitary felid (with a body mass of 9–13 kg and a longevity of up to 11 years) considered as a rabbit specialist and mainly active at night and twilight^47^. The black kite (hereafter kite) is a medium-sized, diurnal, long-lived, opportunistic raptor (with a body mass of 0.8–1 kg, a wingspan of about 1.5 m and a longevity of up to 30 years) that preys heavily on rabbits when these are abundant^48^. The European rabbit (hereafter rabbit) is a small herbivore (around 1.1 kg) and a keystone prey for over 30 Mediterranean predators^49^.

**Extended Data Fig. 1:**
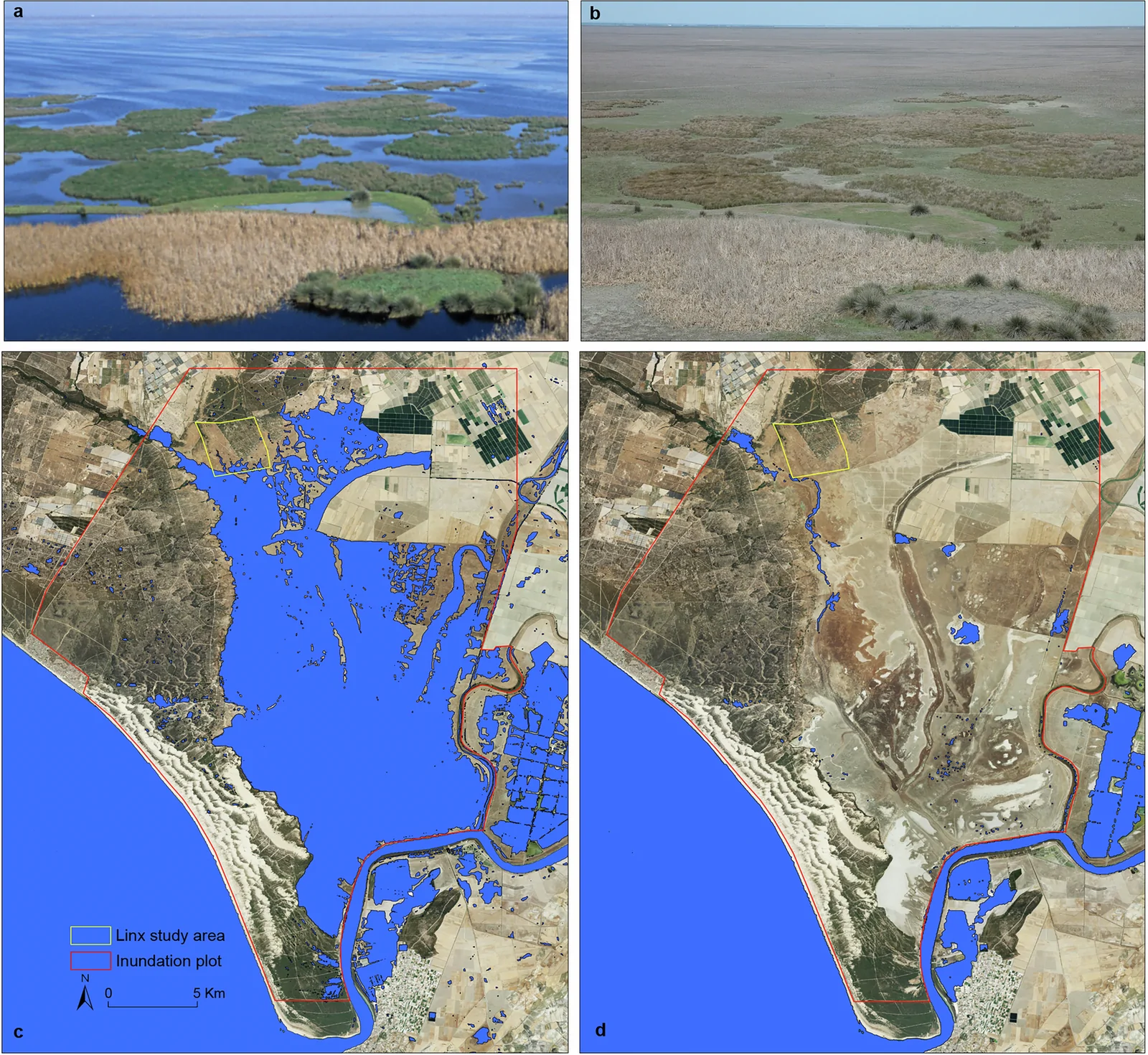
Drought conditions change radically the ecosystem of Doñana National Park (southwestern Spain). In a typical, “normal” year, autumn-winter rainfall causes extensive flooding of vast expanses of land of Doñana National Park (southwestern Spain) (images of left column, panels **a** and **c**). The extent of such seasonal marshland peaks in February-March and then surface water progressively declines due to increasing spring-summer evapotranspiration. In exceptional drought years, however, autumn-winter rainfall is extremely scarce and the marshes flood little if at all (images of right column, panels **b** and **d**). By February-March in a drought year, the majority of the park appears as brown-to-grey dry soil with scarce vegetation, in stark contrast with the luxuriant, green-blue looking ecosystem of normal years. Yellow polygon: intensive study plot in which we examined lynx predation on kites. Red polygon: plot used for the calculation of inundation rates (see Methods). Landsat inundation mask examples from 3 April 2010 (panel **c**) and 27 March 1993 (panel **d**); background basemap image from PNOA 2025, CC BY 4.0 (scne.es).

**Extended Data Fig. 2:**
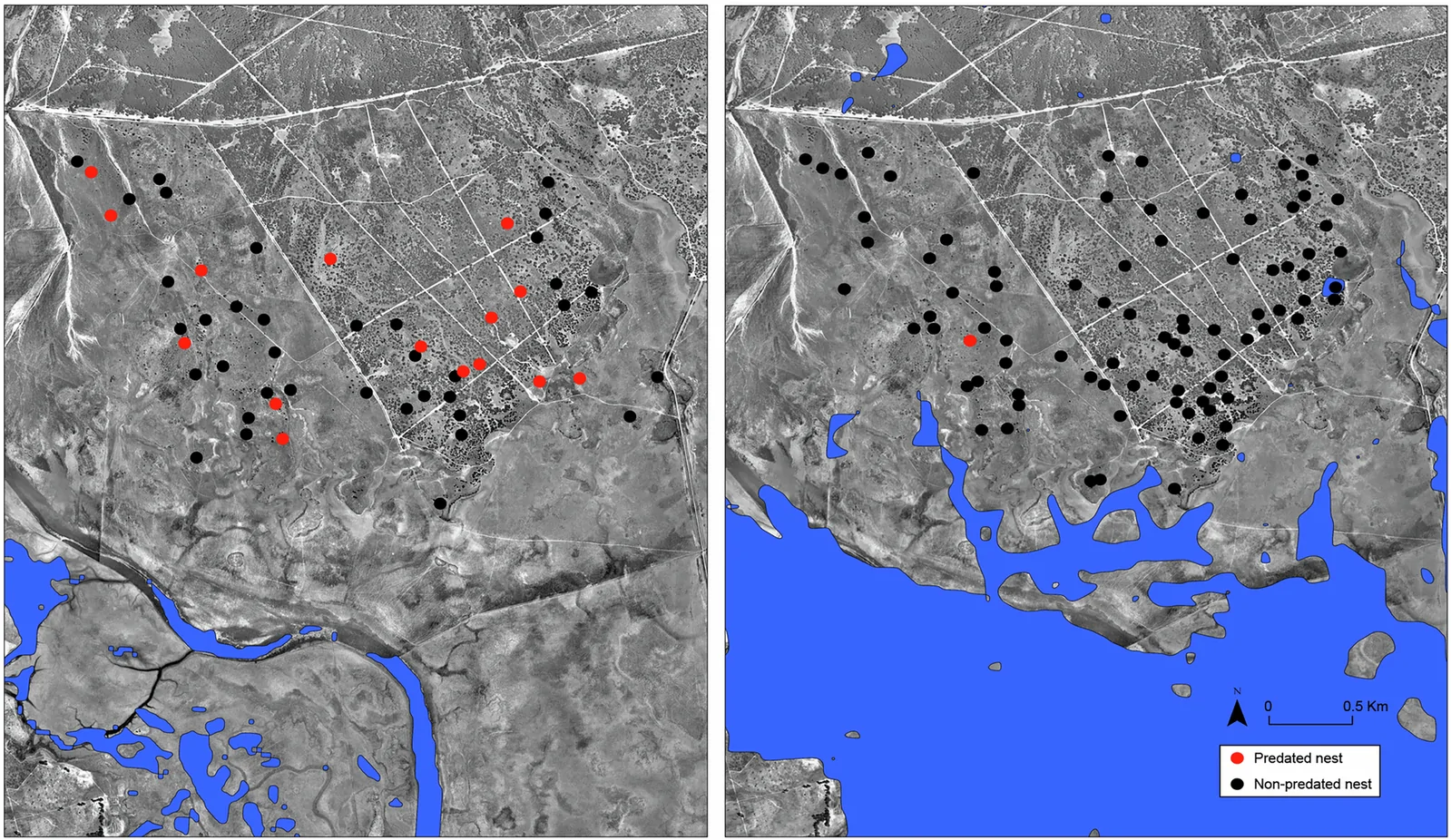
Examples of lynx predation on raptorial black kites under drought conditions (left panel) and under non-drought conditions with well-inundated wetlands (right panel). Black dots show kite nests that reached the stage of well-feathered nestlings and thus available for lynx predation (see Methods). Red dots indicate black kite nests predated by lynx. Blue patches indicate inundated areas, as recorded through Landsat thermic mapper (TM) and enhanced thermic mapper (ETM+) images. Landsat inundation layers have a pixel-size of 30 m, so they do not show the myriad smaller ponds (<900 m^2^) which would have been scattered throughout the terrestrial part of the study area in the right panel (the left panel would be unaffected by their inclusion). Thus, the images show a conservative view of the difference in inundation between drought and non-drought years. Landsat inundation mask examples from 21 March 2011 (right) and 30 March 2023 (left): background basemap image from Orto-SIGPAC 1997–2003, CC BY 4.0 (scne.es).

**Extended Data Fig. 3:**
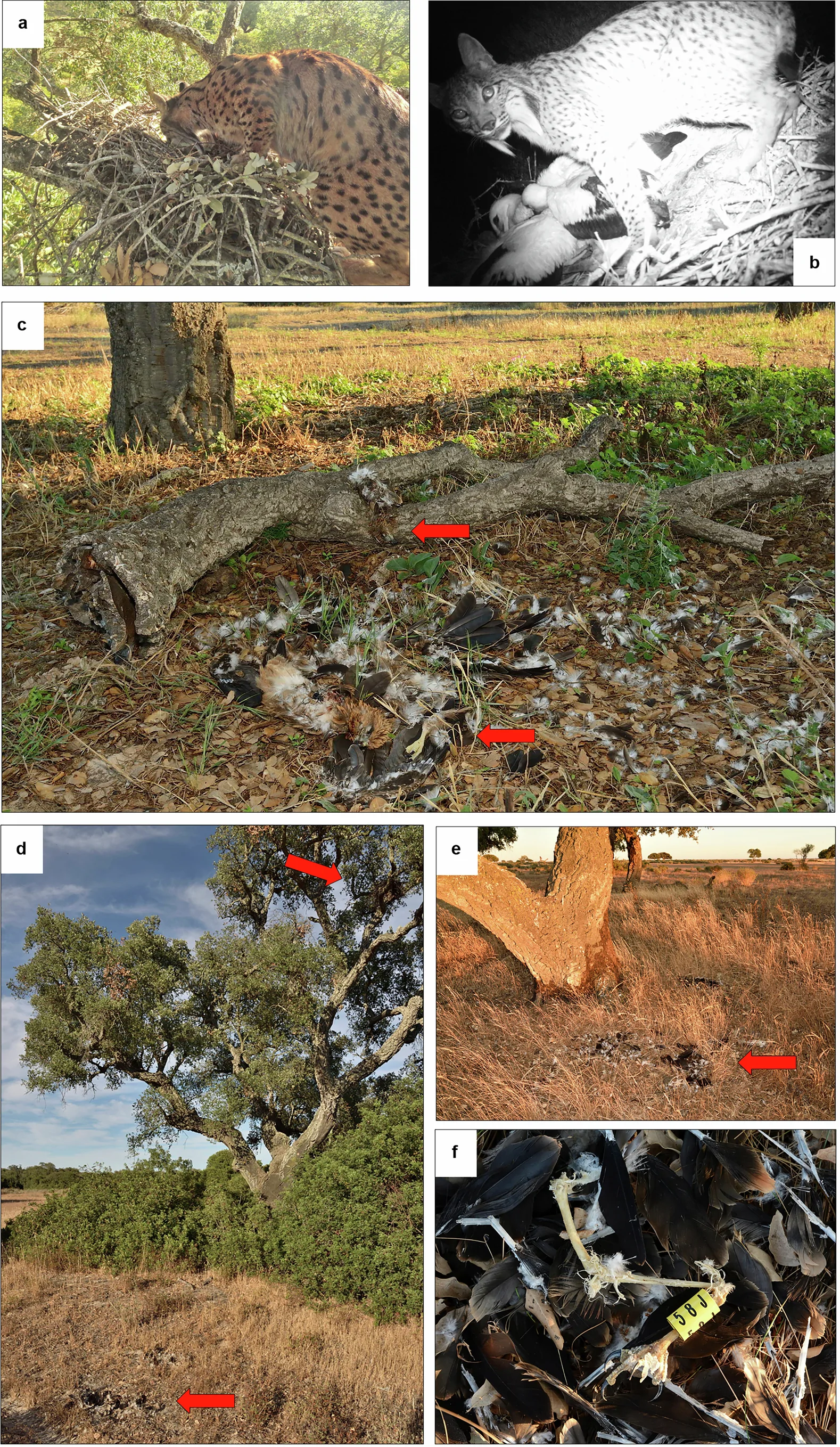
Lynx predation on avian nests on trees. Lynx are very adept tree climbers and, in years of rabbit scarcity, broaden their diet to include nestlings of large birds, such as raptors (panel **a**) or storks (**b**). (**c**) Dawn reveals the forensic signature of lynx predation on two kite fledglings consumed just below their nest (red arrows). (**d**) Plucking of a kite nestling (lower red arrow) transported on the ground from its nest 8 m up a cork oak (upper red arrow). (**e**) Nestling remains (red arrow) are still visible weeks after predation. (**f**) Ringed kite nestling consumed by lynx, showing the typical carnivore signature of chewed and severed feather sheets (Photo credits: panel a: F. Sergio and J. Chicano; panel b: J. Blas, S. Palacios and F. Torres; panel c-f: F. Sergio).

We monitored the predatory interaction between lynx and kites for three decades within a 10-km^2^ sector of Doñana National Park—a wetland in southern Spain that inundates seasonally, but is periodically affected by year-round drought (Extended Data Figs. 1 and 2) that severely curtails prey availability for most predators (for example, 4–5-fold reductions in prey^50^). Climate models forecast a substantial increase in drought frequency for this region over the next 30–100 years^51–53^, which makes this predator-rich ecosystem an ideal candidate to explore the interplay between climatic forcing and interactions among top predatory vertebrates. The study plot supported a relatively stable population of 4–6 adult lynxes (2–3 males and 2–3 females)^47^ and 105–223 kite pairs, representing 82–90% of the local raptor assemblage. These two species can therefore be considered the main diurnal (kite) and nocturnal (lynx) top predators of the ecosystem. In this area, lynx diet is dominated by rabbits (70–90% of the diet), but expands during periods of rabbit scarcity to include alternative prey, mainly in the form of waterbirds from the marshes, especially ducks^54,55^. During these prey troughs, lynx can also depredate tree nests of large birds such as raptors and storks (Extended Data Fig. 3). Similarly, kite diet is dominated by rabbits when these are abundant (up to 80% of the diet, with a mean of 58% before the current rabbit crash) and broadens when these are less available to include birds, fish, reptiles and crayfish mainly captured in the marshes^48^. Kites are capable of killing adult rabbits, which are usually dismembered and taken to the nest in two-to-three sequential deliveries.

In 2013, the local rabbit population started to decline severely (by over 70%) owing to a new variant of the rabbit haemorrhagic disease^56^. The virus originated from a breeding farm in France and spread rapidly from farm to farm, and to surrounding wild populations throughout the Iberian Peninsula^56^, thus representing a form of anthropogenic, global change impact. The crash in main rabbit prey and the predators’ dietary overlap over alternative marshland prey—which is in turn affected by drought—offered a unique opportunity to study how top predators interact under increasing competition imposed by a global change driver and multiple bouts of drought. Below, we use the term ‘intraguild predation’ interchangeably with ‘lynx predation on kites’.

**Fig. 2:**
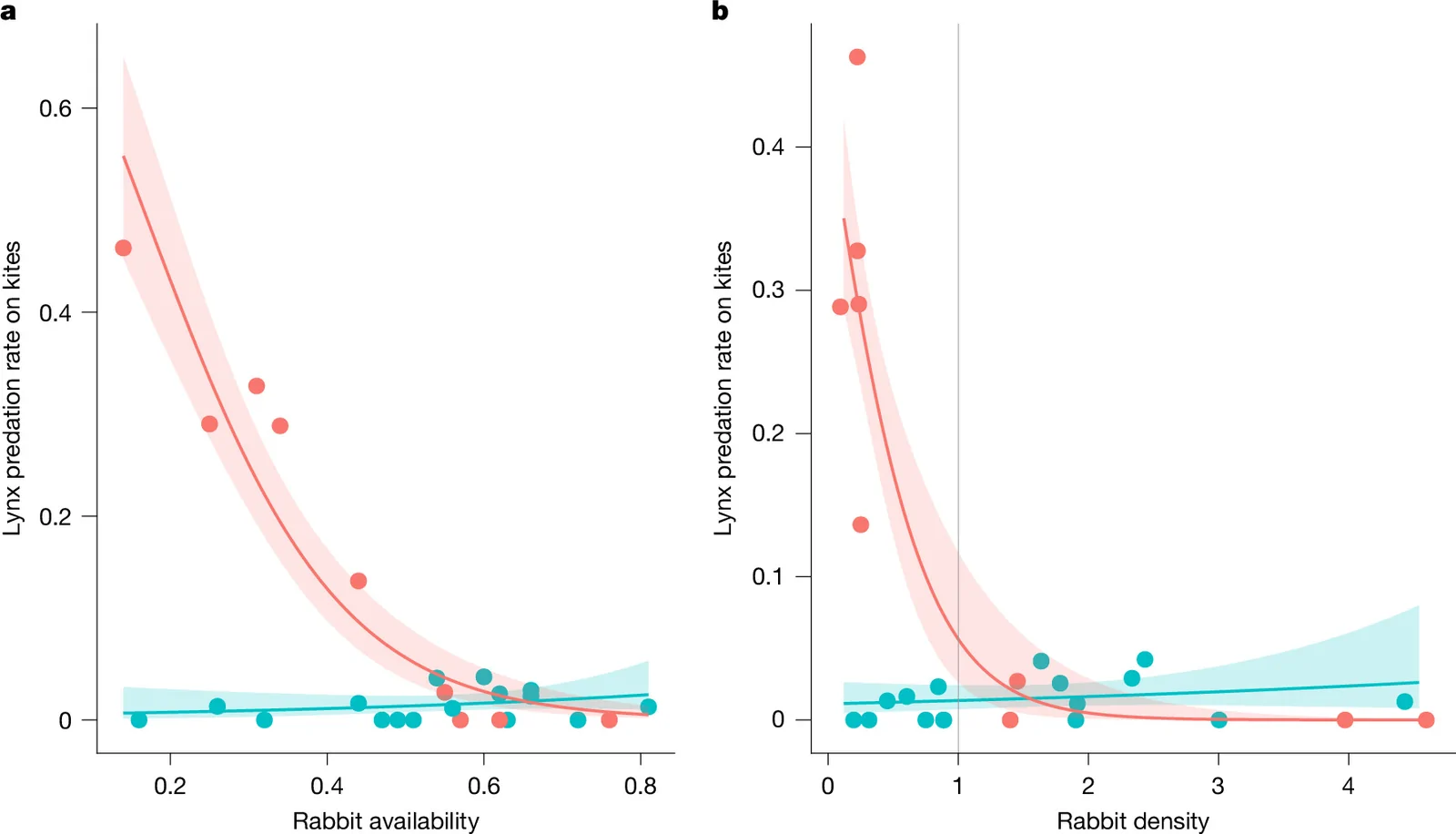
Intraguild predation by a mammalian carnivore (lynx) on raptorial black kites increased with declining density and availability of shared rabbit prey, but exclusively in drought years. Drought conditions are shown in red, whereas non-drought conditions are shown in turquoise. **a**, Rabbit availability was estimated as the proportion of rabbits in the black kite diet. **b**, Rabbit density was the annual, spring density of rabbits (individuals per hectare), as assessed by distance sampling along standardized survey-transects. Lines show the fit of a binomial generalized linear model (GLM) for proportional data with 95% confidence bands (**a**, *β*_interaction_ = −10.15 ± 2.00, *P* < 0.0001, *R*^2^ = 0.87; **b**, *β*_interaction_ = −2.69 ± 0.56, *P* < 0.0001, *R*^2^ = 0.86, *n* = 25 years of study). The thin grey line in **b** represents a density of one rabbit per hectare, which was ascertained as the minimum requirement for lynx subsistence and population stability^47^. Points were horizontally jittered to avoid overlap. Refer to Extended Data Table 1a,b for model details.

**Fig. 3:**
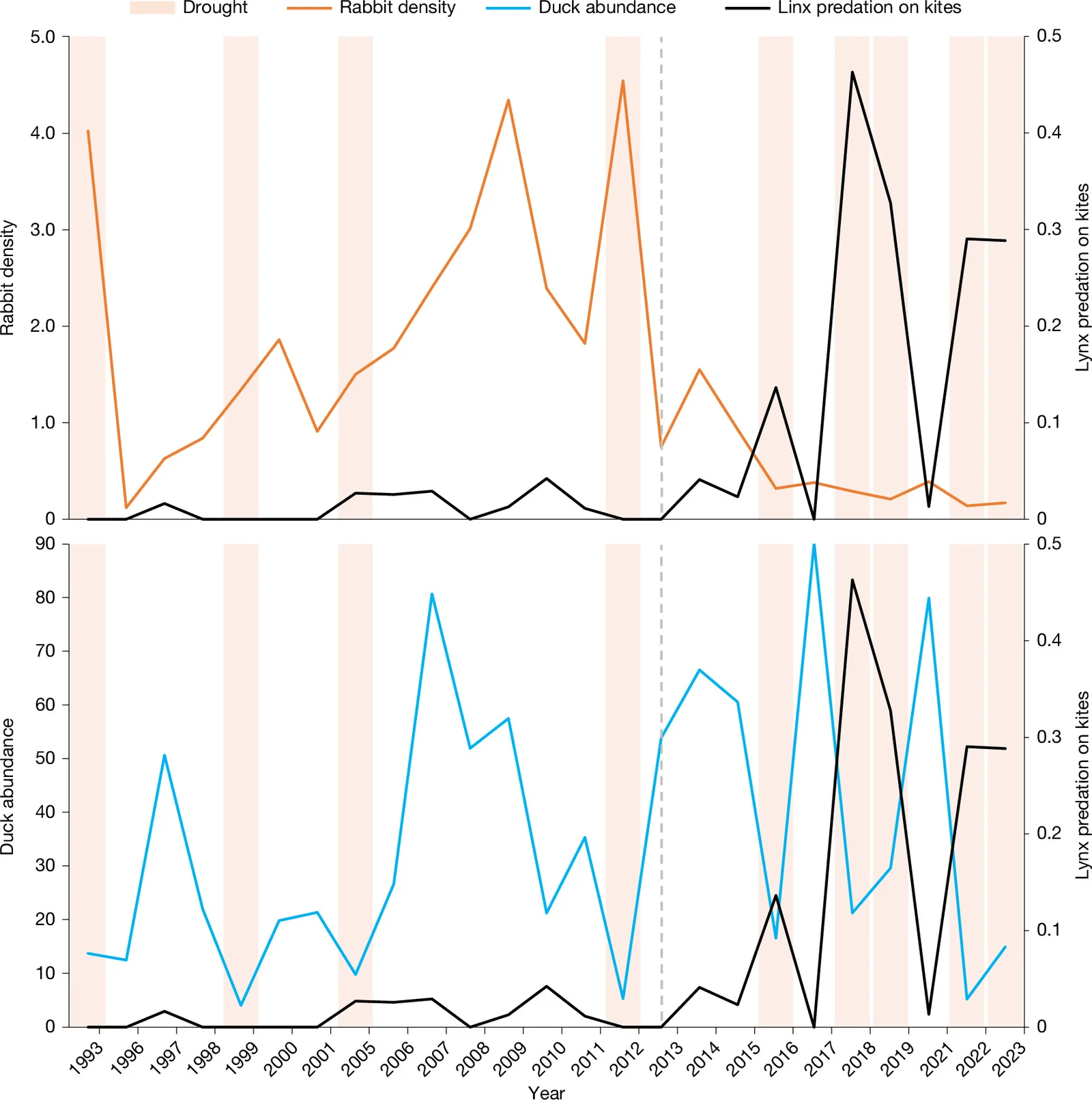
The crash in shared rabbit prey in 2013 caused peaks of intraguild predation by lynx on raptorial black kites, but only when drought conditions curtailed the availability of alternative lynx prey such as ducks. The density of rabbits (orange line, upper panel) suddenly dropped after 2013 (grey dashed line) as a result of an anthropogenically caused infectious disease. This led to bursts of lynx predation on kites (black line) during drought (orange shading), which levelled down alternative lynx prey such as ducks (blue line, lower panel). Rabbit density was the annual, spring density of rabbits (individuals per hectare), as assessed by distance sampling along standardized survey-transects conducted since 2005. For the years before 2005, rabbit density was estimated by a regression of rabbit density on rabbit occurrence in kite diet (Methods). Duck abundance was assessed by standardized aerial spring counts and is expressed in thousands of individuals (Methods).

Data from 1,624 kite nests cumulatively monitored over three decades showed that intraguild predation was strongly tied to the interaction between the availability of shared prey and drought (*R*^2^ = 0.87, Fig. 2). In particular, lynx and kites basically acted neutrally towards each other for two decades (Fig. 3): lynx predation on kite nestlings was occasional and destroyed only 1% of kite nests (13 of 904 nests between 1993–2012). When an emergent disease triggered the rabbit crash, lynx predation on kite nests increased exponentially with rabbit decline, but exclusively during years of drought (red points and lines in Fig. 2a,b; see also Extended Data Table 1a,b), when the availability of alternative prey such as ducks was extremely reduced (blue line in Fig. 3; see also Extended Data Table 1c). During non-drought years, lynx predation remained negligible regardless of the level of rabbit abundance (turquoise points and lines in Fig. 2a,b), as lynx probably compensated the rabbit scarcity with abundant alternative prey, such as waterbirds from the nearby well-inundated marshes and ponds^54,55^ (blue line of Fig. 3; see also Extended Data Fig. 2 and Extended Data Table 1c). Intraguild predation was therefore modulated by drought, which switched lynx predation on kites on and off. Notably, lynx predation took off in drought years but only when rabbit density dropped to less than one rabbit per hectare (grey line in Fig. 2b), which was ascertained as the minimum requirement for lynx subsistence and population stability^47^. At very low rabbit densities during drought, intraguild predation was severe, destroying up to 46% of kite nestlings close to fledgling and thus removing almost half of the local cohort (Figs. 2 and 3).

**Fig. 4:**
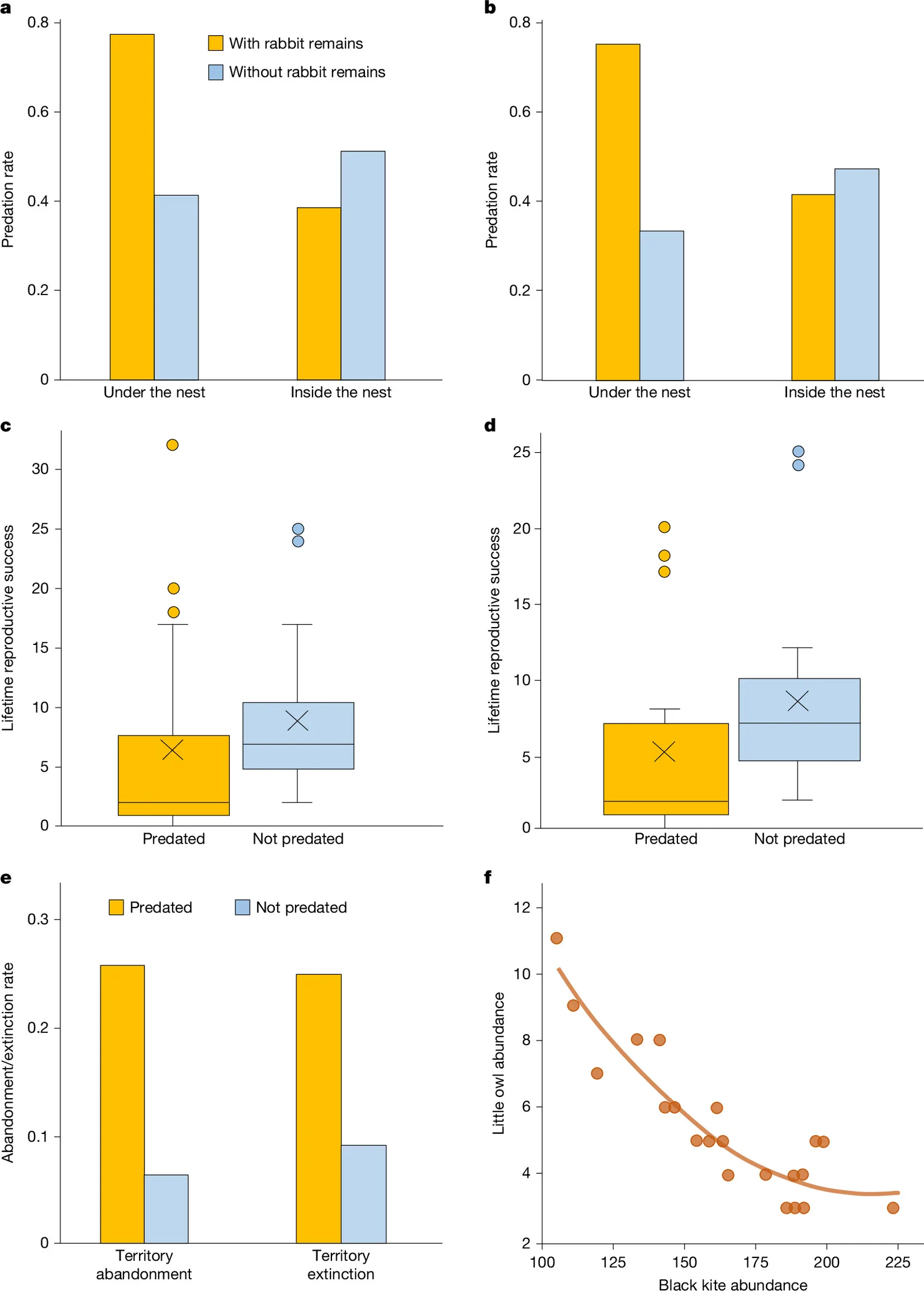
Intraguild predation by lynx on raptorial black kites preferentially targeted broods with rabbit remains under the raptor nest, depressed kite fitness and induced territory emigration and extinction, with potential cascading benefits for a lower-trophic-level insectivore. **a**,**b**, The frequency of lynx predation was higher for kite nests with rabbit remains on the ground than for those without, when analysed by both prey remains (**a**, *χ*^2^ = 7.96, *P* = 0.005, *n* = 108) or by nest (**b**, *χ*^2^ = 8.79, *P* = 0.003, *n* = 41). No such difference was evident for prey remains inside of the nest (**a**, *χ*^2^ = 0.87, *P* = 0.35, *n* = 354; **b**, *χ*^2^ = 0.14, *P* = 0.71, *n* = 75). **c**,**d**, Intraguild predation depressed the number of fledglings that kites generated throughout their life, regardless of including (**c**) or excluding (**d**) an outlier kite with a 28% higher lifetime reproductive success than any other individual (*β*_with-outlier_ = −0.33 ± 0.12, *P* = 0.03, *n* = 40; *β*_without-outlier_ = −0.51 ± 0.13, *P* < 0.0001, *n* = 39; Extended Data Table 1g,h). **e**, Emigration to other territories and territory extinction were more frequent after intraguild predation (*β*_emigration_ = −3.25 ± 1.32, *P* = 0.026, *n* = 62; *β*_extinction_ = 1.18 ± 0.40, *P* = 0.002, *n* = 216; Extended Data Table 1i,j). **f**, Finally, lowered kite density was associated with increased abundance of an insectivorous owl (*β* = 5.56 ± 1.91, *P* = 0.009, *n* = 19; Extended Data Table 1l), suggesting that the intraguild predation pressure had the potential to ripple down the food web. In each box plot, the centre line represents the median, the X symbol indicates the mean value, the length of the box extends through the interquartile range (IQR) and the whiskers extend to 1.5× IQR. All data points outside of this range were plotted individually. In **c** and **d**, the colour legend refers to the predation of the brood of an adult individual, not predation of the adult individual. Refer to Extended Data Table 1 for details on the models.

**Extended Data Fig. 4:**
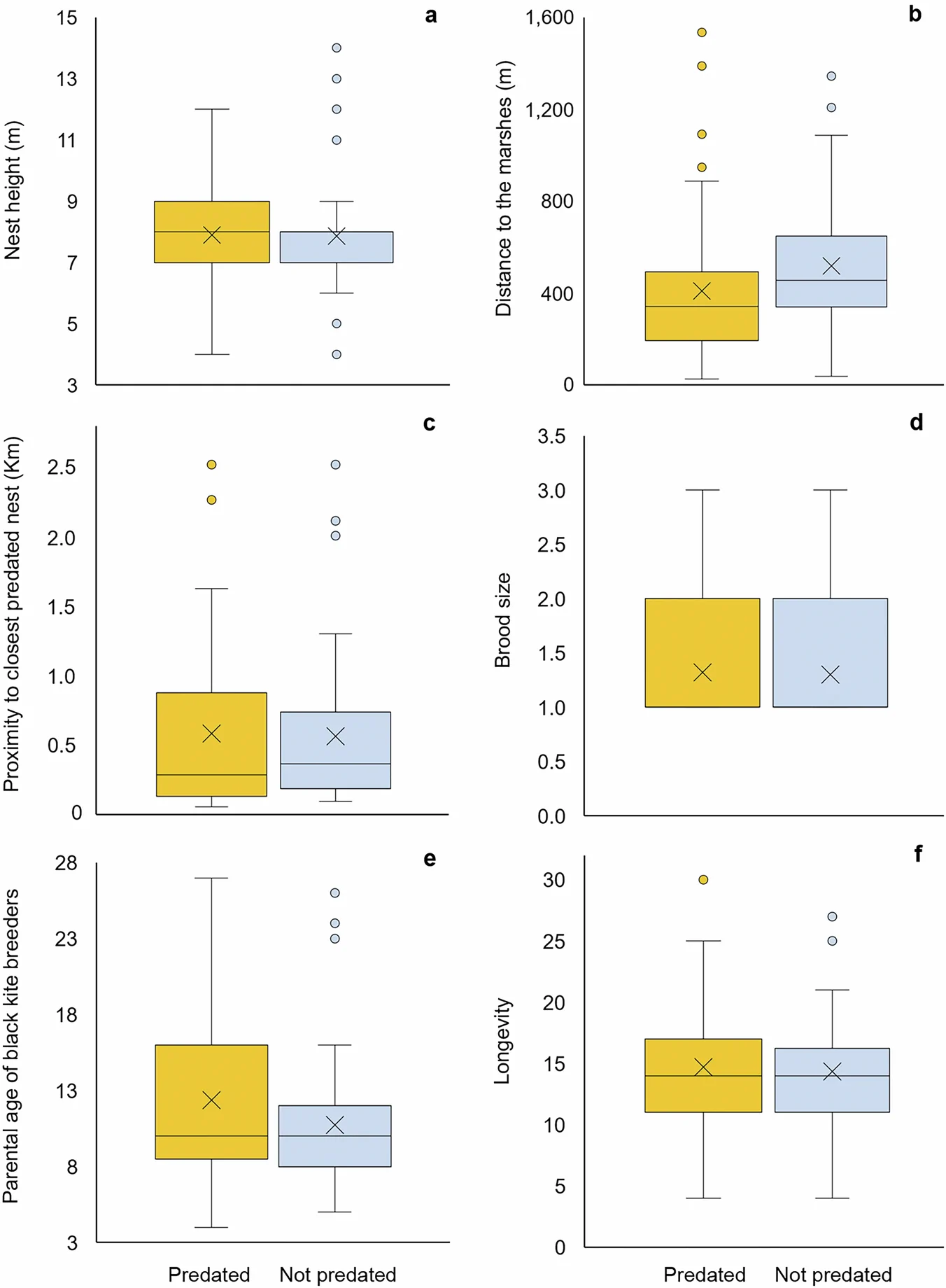
Intraguild predation by lynx on raptorial black kites did not target specific characteristics of kite nests or individuals. Black kites that suffered brood predation by lynx did not differ from non-predated individuals in terms of nest height (panel **a**), proximity to wetlands (**b**) distance to the closest lynx-predated nest (**c**), number of nestlings in the brood (**d**), parental age (**e**) and longevity (**f**) (β_nest height_ = −0.05 ± 0.15, P > 0.05, n = 216; β_proximity to marshes_ = −0.13 ± 0.15, P > 0.05, n = 216; β_proximity predated nest_ = 0.01 ± 0.14, n = 216, P > 0.05; β_brood size_ = −0.08 ± 0.13, n = 216, P > 0.05; β_age_ = 0.06 ± 0.04, P > 0.05, n = 82; β_longevity_ = 0.02 ± 0.08, P > 0.05, n = 40; Extended Data Table 1d–f). Symbols: in each boxplot, the center line represents the median, the “X” symbol the mean value, the length of the box extends through the interquartile range (IQR) and the whiskers extend to 1.5 × IQR. All data points outside this range are plotted individually. For panel (**f**), the color legend refers to the predation of the brood of an adult individual, not the predation of the adult individual. Model details can be found in Extended Data Table 1.

Kite nests predated by lynx did not differ from non-predated nests in terms of the nest height, proximity to the marshes, distance to other lynx-predated nests, number of chicks in the brood or the age of the parent kites (Extended Data Table 1d,e and Extended Data Fig. 4a–e). Thus, kite experience did not prevent predation, which was neither concentrated in specific areas nor induced by nest placement or content (for example, by attraction to more frequent begging for food by larger broods). Lynx are extremely good tree climbers: they predated nests up to 12 m high and patrolled 8 km of their territory, on average, daily^47^. Thus, all nests could be considered at risk and there was no apparent nest-placement strategy available to kites to avoid predation. Furthermore, it would presumably be easy for lynx on the ground to know that a kite nest contains a brood as well as its approximate age, because the area below nests progressively accumulates white excrements and prey remains—a form of public information that lynx probably exploited. In particular, compared with non-predated nests, nests predated by lynx had over three times more rabbit remains on the ground below (Fig. 4a,b; *χ*^2^ = 7.96, *P* = 0.005; *N* = 108 prey items; *χ*^2^ = 8.79, *P* = 0.003, *N* = 41 nests), while there was no such difference for the prey remains observed inside the nest (Fig. 4a,b; *χ*^2^ = 0.87, *P* = 0.35; *N* = 354 prey items; *χ*^2^ = 0.14, *P* = 0.71, *N* = 75 nests). One interpretation of these results is that lynx used the ground presence of rabbit remains to decide whether to climb and predate a nest, consistent with the idea of an intraguild killer identifying a competitor and acting to lower its food requirements during potential starvation years. An alternative hypothesis is that lynx learnt to associate rabbits on the ground with a larger food reward in kite nests; however, nests with rabbit remains that had fallen on the ground did not have larger broods (mean ± s.e.m. without rabbit remains: 1.62 ± 0.14 nestlings, *N* = 21 nests; with rabbit remains: 1.25 ± 0.10 nestlings, *N* = 20 nests; *t* = 1.83, *P* = 0.07) or a larger cumulative mass of the brood (without rabbit remains: 1,073.3 ± 105.4 g, *N* = 15 nests; with rabbit remains: 958.8 ± 97.4 g, *N* = 20 nests; *t* = 0.80, *P* = 0.43) than nests without such remains on the ground. If anything, the average food reward was lower for a lynx cueing on rabbit remains under the nest. We therefore find the first interpretation to be the most plausible, although further manipulative experiments will be required to confirm it.

Lynx predation affected kite fitness. Compared to control individuals of the same age, kite parents that suffered predation had similar longevities, but lower lifetime reproductive success (Fig. 4c,d, Extended Data Table 1f–h and Extended Data Fig. 4f). On average, intraguild predation depressed lifetime reproductive success by 27.7%. Kites were clearly not capable of compensating for the lost broods in subsequent years. This demonstrates the fitness consequences of intraguild predation on vertebrate predators and highlights the fragility of such low-fecundity species to new threats. At the same time, this fragility qualifies them as good sentinels of ecosystem pressures, including the indirect impacts of climate anomalies and an anthropogenically driven infectious disease described in this study.

Kites were not passive victims. Individuals that suffered lynx predation were four times more likely to emigrate to another territory the next year (Extended Data Table 1i and Fig. 4e), suggesting that the two predators were locked in an arms race of competitor removal and evasive counter-measures by the victim. Furthermore, the original abandoned territories were rarely taken over by other kites, probably because the remains of predated fledglings remained visible for over a month (Methods) and new kite recruits typically decide where to settle in the previous year, when they intensively prospect the performance of a limited number of territories^57,58^. As a consequence, territories that suffered predation were three times more likely to be vacant the next year (Fig. 3e and Extended Data Table 1j) and this probably contributed to the fact that kite population abundance declined with lynx predation pressure in the previous year (Extended Data Table 1k). In turn, kite abundance depressed the density of an insectivorous bird that is preyed on by kites, the little owl *Athene noctua* (Fig. 4f and Extended Data Table 1l). As a result, little owl density was positively related to lynx predation on kites in the previous year (Extended Data Table 1m), consistent with the idea that the intraguild predation signal had the potential to reach lower trophic levels. These results illustrate the indirect, complex mechanisms by which climate change can impose declines, redistributions and extinctions through its impact on biotic interactions, with potential repercussions on other components of the ecosystem.

Overall, global change in the form of an emergent disease heightened predator interactions, leading to strong bouts of predation never observed before and triggered by drought. Thus, a global change driver amplified the sensitivity to climatic pertubation of a biotic interaction, with cascading consequences for fitness, top-down forcing and lower-trophic-level species. This sequence of one anthropogenic driver that predisposed the system to a heightened impact by another pertubation will probably become more widespread as global change advances^17,18,59,60^. Our results show how this combined impact can generate unexpected synergies, such as the new interaction among disease, drought and intraguild predation for kites. The implication of these dynamics is that the first of the two interacting drivers may weaken the system for years in a cryptic manner that becomes visible only when the second driver comes into play (in our case drought), by which time management may be ineffective. These results confirm the importance of comprehensive management strategies tackling multiple anthropogenic threats, when this is feasible, as each one may amplify the impact of others^17,18^.

More specifically, drought switched intraguild predation on and off, thus acting as its key modulator and reshaping the strength and direction of the interaction, as if a new top predator had invaded the system. As a result, after decades of neutral relations, the top predatory carnivore started to feed at a higher trophic level and a previously abundant avian top predator became a declining mesopredator. This provides an exemplary illustration of how climate change can rapidly transition vertebrate predatory guilds to new, ‘no analogue’ assemblages by altering per-capita effects through interaction rewiring^10,22^, even without invasion by new players. The implications of these results are manifold. First, dietary plasticity and the capability to exert a new predatory interaction probably enabled the Iberian lynx—traditionally portrayed as a rabbit-dependent specialist—to persist in the face of extremely low rabbit densities and even during drought-driven scarcity of alternative prey. This confirms recent findings showing that some species may be better equipped with (unexpected) resilience mechanisms than previously thought^23–26^. It also supports the trait-based sensitivity hypothesis, by which plasticity in key traits, such as diet composition and mobility, may predispose or safeguard certain species from the impact of stressors^23^. In our case, all of the species involved seemed in one way or another to enact some stressor-buffering strategies: lynx expanded its diet and interaction modalities, kites relocated to avoid lynx impacts and the owls moved in to exploit predator-free space. Collectively, these dynamics reinforce the notion of mobility and diet plasticity as the key traits enabling resistance to stressors^23^. Second, species niches and functional roles should be seen as flexible and dynamic, as their nature, strength and direction may radically change in response to stressors with profound positive or negative fitness consequences, even across distant taxa with apparently separate niches (for example, diurnal versus nocturnal). Third, these dynamic changes can develop rapidly (2–3 years in our case). Fourth, when climatic perturbation reshuffles key interactions, even ‘weedy’, abundant species may become endangered in rapid, unexpected manners. In our case, a mobile generalist, usually considered a winner of climate adaptation (see, for example, refs. ^3,22^), was rapidly and unexpectedly limited by a specialist predator, frequently considered a loser.

This study offers important reflections on the role of top predators in ecosystems. First, it confirms their highly interactive role, but highlights how such a role can be fluid and conditioned by external factors: in our case, top-down forcing that was absent for decades became suddenly apparent, once activated by drought. Such context-dependency stresses the need to maintain the full diversity of predator assemblages to maintain their full functionality, which may become evident only over longer time scales. Second, our results lend proof to the proposed role of top predators as biotic multipliers^38,39^, that is, species capable of amplifying the impact of climate change on their communities. Lynx predation was driven by drought, but in turn exacerbated the impact of drought on kites, because it removed feathered nestlings that typically do not die anymore (survival > 97%, *N* = 70 GPS-tagged nestlings). Thus, the top predator amplified drought with potential cascading consequences for other members of the wider community, such as the insectivorous owl of this study.

As climate change progresses in concert with other anthropogenic stressors, identifying traits and processes that promote population persistence will be of primary importance. In this context, our results bring a mix of positive and negative implications. On the negative side, forecasting the biodiversity effects of climate change may be challenging^1,60^, given the interactive and unexpected dynamics observed even in our small trophic module of 3–4 species. On the positive side, certain ecological traits (dietary flexibility, mobility) and keystone roles (upper-trophic-level position, biotic multiplier) generated predictable direct and indirect outcomes. These could be exploited to devise proactive strategies that mitigate impacts on target species of interest. For example, in our system, a manager interested in maintaining the previous status quo of the raptor community with abundant kite populations could provide supplementary feeding to lynx in drought years, to dampen the spikes of predation on raptors. In the future, there is a high need for other process-based studies and meta-analyses that: (1) identify predictors of the impact of stressors on populations, species and assemblages; and (2) test the extent to which trait plasticity, niche breadth flexibility and ecological rewiring are sufficient to buffer populations from anthropogenic stressors over the longer-term. In conclusion, climate change is likely to reshape interactions, transform ecological roles and alter top-down forcing in predatory guilds, with likely cascading consequences for whole ecosystems. The key to forecasting the future impact of climate and global change may reside at the heart of ecology: understanding how ecological flexibility and interacting keystone species respond to interacting shocks.

## Methods

### Study area

The study was conducted in a 10-km^2^ plot in the north of Doñana National Park (Extended Data Figs. 1 and 2)—an iconic wetland of southwestern Spain that inundates seasonally, but is periodically affected by year-round drought that severely curtails prey availability for most predators (for example, 4–5-fold reductions in prey^50^). In a typical, normal year, autumn–winter rainfall causes progressive extensive flooding of vast expanses of land (Extended Data Fig. 1). The extent of such seasonal flooding peaks in February–March and then surface water progressively declines owing to increasing spring–summer evapotranspiration^61^. However, in drought years autumn–winter rainfall is extremely scarce and the marshes flood little if at all. In a drought year, by February–March the majority of the park still appears as brown–grey with scarce vegetation, in stark contrast with the luxuriant, green–blue-looking ecosystem of normal years (Extended Data Fig. 1).

**Extended Data Fig. 5:**
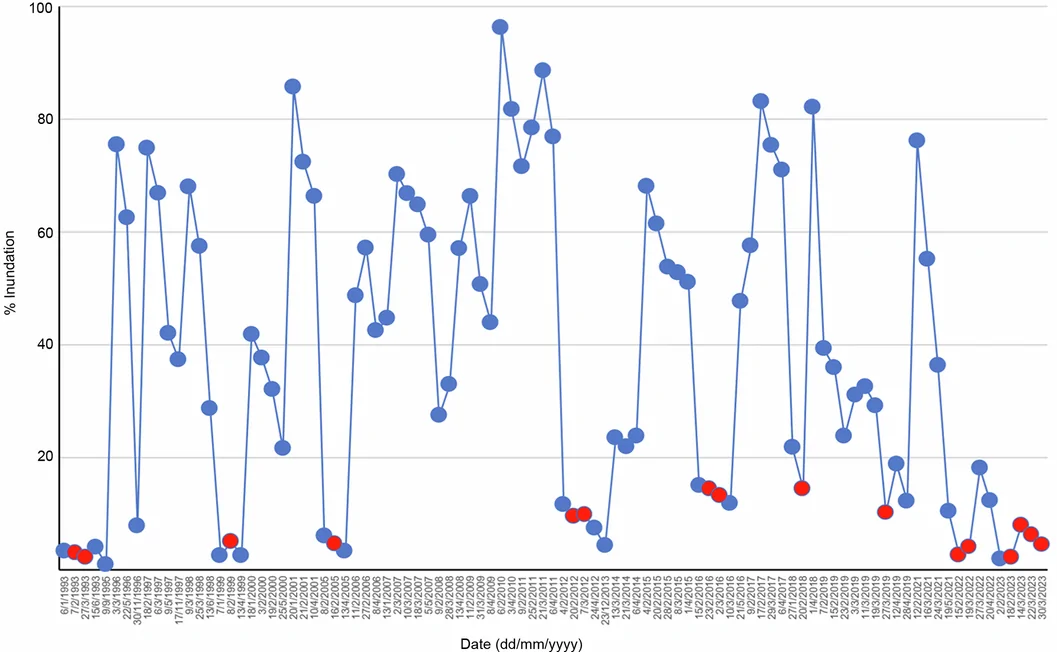
Percentage rate of inundation of the Doñana marshes based on available Landsat images for the years covered by this study. To avoid visual clogging, for each year we show the images available between 15 February and 30 March (see Methods) and the images available just before and just after this temporal window. Red points highlight years in which percentage inundation went below 20% (i.e. ≥ 80% of the marshes were dry) (years: 1993, 1999, 2005, 2012, 2016, 2018, 2019, 2022, 2023).

On the basis of the above and our field experience, here we classify drought conditions as years in which the inundated area fell below 20% of maximum flooding in February–March (that is, over 80% of the marshes were dry; see below). Flooding rates were calculated for an area of 684 km^2^ covering the Doñana marshes, inland ponds and lagoons (Extended Data Fig. 1). The area was chosen so as to characterize the large-scale conditions that apply to the whole ecosystem, because ducks, kites and other potential prey are highly mobile species and thus we expect them to respond not just to the local conditions of the lynx study plot, but to ecosystem-wide conditions. Flooding was assessed by accessing inundation masks provided by the Remote Sensing and Geographical Information System Laboratory (LAST-EBD) of Doñana Biological Station - CSIC; these are routinely used in scientific assessments of the ecological consequences of marsh flooding^62–64^. Inundation masks were generated by accessing cloud-free Landsat thermic mapper and enhanced thermic mapper scenes for the Doñana region. Images were radiometrically normalized to a reference image and transformed into reflectance values to produce final inundation masks at a 30 × 30-m spatial resolution, with each pixel classified as inundated for normalized reflectance values of under 0.186 (Cohen’s statistic of agreement: *κ* = 0.66, overall accuracy = 94%)^63^. Details of the method and its extensive ground-truthing can be found in ref. ^63^ (see also the protocol and code in https://github.com/LAST-EBD/Protocolo_v4). Usable Landsat images require cloud-free days and thus their dates change across years. To make inundation rates comparable across years, we linearly interpolated consecutive inundation masks so as to estimate the inundated area for the fixed dates of 15 February, 1 March, 15 March and 30 March every year. We then extracted the minimum inundated area reached on these fixed dates and classified a year as drought when such a level was lower than 20% of the maximum inundation recorded since 1975 (365.7 km^2^), that is, when over 80% of the marshes were dry. So defined, of the study years used in the current analysis, drought conditions occurred in 1993, 1999, 2005, 2012, 2016, 2018, 2019, 2022 and 2023. A plot of the real (non-interpolated) inundation rates based on actual Landsat images confirmed these years in which marsh inundation fell below 20% as troughs (red dots in Extended Data Fig. 5). The troughs in the abundance of ducks (waterbirds obligatorily dependent on inundation) also confirmed the above years as inundation troughs (Fig. 3). Finally, for 2013 there were too few Landsat images to enable proper interpolation and thus we estimated the minimum inundation in February–March using the regression equation ‘1.05 × (inundation on 15 April) – 1.2754” (*R*^2^ = 0.91)’. Inclusion or exclusion of this year did not change the results. More inundation data are available from the Copernicus program Sentinel-2, but they were not integrated because they have different resolutions and have only been available since 2017. It is possible that the inclusion of a richer time-series could have improved the robustness of the inundation mapping.

In recent times, climate anomalies (extreme temperatures or rain during key periods) have caused new patterns of drought. For example, in 2019 record temperatures in February–March precipitated a winter of moderate inundation into a sudden spring drought, whereas in 2018 extremely severe rainfall converted an initial spring drought into an extremely late and sudden flooding of the marshes. Such late rains and inundation are likely to aggravate the deleterious effects of drought, for example, because already-formed waterbirds’ nests and colonies will be washed out by the sudden rise in water levels (pers. obs.). The deleterious effects of late inundations (dry to wet reversals) have also been shown in other seasonal wetlands^65,66^. To more explicitly incorporate such anomalous dynamics, we also classified each year as climatically anomalous when: (1) its inundation levels fell below 20% of maximum flooding in February–March (as above); or (2) it had extremely severe drying conditions in early spring, which was estimated as an average daily maximum temperature for February–March that was higher than the 95th percentile of its values-distribution recorded since 1979 (21.6 °C); or (3) it experienced extreme, late rainfall, estimated as a cumulative rainfall between 15 February and 15 May higher than the 95th percentile of its values-distribution recorded since 1979 (270 mm). Hereafter we refer to these years as climate anomalies or anomalies. So defined, anomalous years coincided with the drought years listed above, but with the addition of 2011, given its copious spring rains. Data on daily temperature and rainfall came from a weather recording station located within Doñana National Park (courtesy of the Infraestructura Científico-Técnica Singular - Reserva Biológica de Doñana (ICTS-RBD) of Doñana Biological Station - CSIC).

In Doñana, spring temperatures have been increasing at a rate of over 0.5 °C per decade and climate models forecast a substantial increase in drought frequency and intensity for the general region over the near future^51–53^, which makes this predator-rich ecosystem an ideal and urgent candidate to explore the interplay between climatic forcing and interactions among top predatory vertebrates. The main vegetation of the study plot is Mediterranean scrubland and grassland with scattered cork oaks *Quercus suber*, bordered by open marshland (marisma) (Extended Data Figs. 2 and 3). During drought years, the open marshland converts into a dry steppe with scarce, short vegetation or dry soil.

#### Drought terminology

Here we hypothesized that lynx could be strongly disadvantaged when low availability of rabbits (the lynx’ main prey) is compounded with low availability of ducks (the main alternative prey). As ducks are waterbirds that are obviously dependent on water, our analyses are centered on the impact of very low surface water availability, and thus focus strongly on so-called hydrological drought. Such water availability is strongly driven but not completely determined by meteorology. For example, cumulative rainfall between 1 October and 1 March explains 74% of the among-year variation in the percentage of the marshes inundated on 1 March (*β* = 0.49 ± 0.05, *t* = 9.72, *P* < 0.0001). Thus, discriminating between hydrological and meteorological drought can be complex and beyond the scope of this paper, whose objective is to examine the wildlife consequences, not the causes of drought. For simplicity, here we use the generic term drought to indicate hydrological drought, estimated as very low surface water availability (that is, years in which more than 80% of the wetlands are dry). In our field experience, these are years characterized by high aridity and very low prey availability for all predators in the ecosystem. We acknowledge that, as with any extreme event, other classifications could be possible. However, models based on alternative continuous estimates that we could think of (including inundated area on key dates or cumulative winter rainfall) gave the same conceptual results (for example, highly significant interactions between these estimates and rabbit abundance), but much worse model fit (over 10 AICc-units increments in the Akaike Information Criterion corrected for small sample size). Throughout the paper, the generic term drought could be used interchangeably with hydrological drought or very low surface water availability.

### Study system

The study focused on an intraguild predation module comprising a mammalian carnivore (that is, the Iberian lynx) and a raptor (that is, the black kite), which compete for shared rabbit prey. Community modules of this type are considered ideal model-systems to gain the necessary mechanistic understanding of interactions that are simultaneously vulnerable to climate and key determinants of community processes, function and structure—an essential prerequisite for predicting community responses to climate change^2^.

The Iberian lynx is a medium-sized solitary felid (body mass 9–13 kg, longevity up to 11 years^67^), mainly active at night and twilight^68^ and considered as a rabbit specialist^47,54,55^ capable of limiting or suppressing other carnivore mesopredators^69–72^. The home range of each male usually coincides with the home range of one female, although the ranges of multiple individuals can overlap to some degree^47,73^. The black kite is a medium-sized, diurnal, long-lived, opportunistic raptor (mass 0.8–1 kg, wingspan of around 1.5 m, longevity up to 30 years^74,75^) that preys heavily on rabbits when these are abundant^48,76^. Kites are well capable of killing adult rabbits (pers. obs.), which are usually dismembered and transported to the nest in 2–3 sequential deliveries. They usually defend a small territory of 50–200 m around the nest and hunt over much larger, undefended foraging areas of 18–52 km^2^ (ref. ^77^). The European rabbit is a small herbivore (about 1.1 kg) originally endemic to the Iberian Peninsula and a keystone prey for over 30 vertebrate predators in the Mediterranean^49,78^.

Our study plot supported a relatively stable population of 4–6 adult lynxes (2–3 males and 2–3 females, corresponding to an average density of 0.77 lynx per km^2^)^47^ and 105–223 kite pairs, representing 82–90 % of the local raptor assemblage. Thus, by virtue of their role and abundance, these two species can be considered the main diurnal (kite) and nocturnal (lynx) top predators of the ecosystem. Although lynx dietary data are not available for Doñana for the recent past, previous work in this area showed that lynx diet is dominated by rabbits (70–99% of the diet) but expands during periods of rabbit scarcity to include alternative prey, mainly in the form of waterbirds from the marshes, predominantly ducks^47,54,55^. During such prey troughs, lynx can also depredate tree nests of large birds, such as raptors and storks (Extended Data Fig. 3). Similarly, kite diet is dominated by rabbits when these are abundant (up to 80% of the diet, mean = 58% before the current rabbit crash) and broadens when these are less available to include birds, fish, reptiles and crayfish mainly captured in the marshes^48^. In 2013, the local rabbit population started to decline severely (by over 70%) owing to a new variant of the rabbit haemorrhagic disease^56^. The virus originated from a breeding farm in France in 2011 and spread rapidly from farm to farm and to surrounding wild populations throughout the Iberian Peninsula^56^, thus representing a form of anthropogenic, global change impact. The crash in main rabbit prey and the predators’ dietary overlap over alternative marshland prey, in turn affected by drought, offered a unique opportunity to study how top predators interact under increasing competition imposed by a global change driver and multiple bouts of drought.

### Field procedures

Kites were censused in the pre-incubation period by looking at territorial displays, transfers of nest materials and the presence of birds perched in or near a nest on multiple days. Whenever possible, nests were checked every 10–15 days and climbed when the nestlings were 40–45 days old to record the number of reared young and ring them (nestlings fledge when 48–50 days old). During each nest visit, we recorded all prey remains observed inside the nest or on the ground under the nest. The nestlings of this population have been ringed since the 1980s with a white or yellow plastic ring engraved with a black, three-character alphanumeric code, which can be read by spotting-scopes from a distance without disturbing the birds^58^ (Fig. 1b). Marked territory holders were searched intensively by visiting territories every 3–5 days throughout the breeding cycle. A marked individual was assigned to a specific territory when it was observed there in more than three separate visits. This allowed us to record the longevity and lifetime reproductive success of several individuals monitored throughout their lives^79^.

Several factors made lynx predation on kite broods highly distinctive. Lynx systematically killed the chicks in the nest and then transported them to the ground below the nest, where they were plucked, or partially plucked, and consumed (Extended Data Fig. 3). Pluckings by mammalian carnivores are easy to separate from raptor pluckings, because carnivores chew and cut off many feathers, while raptors mostly extract them without severing them (Extended Data Fig. 3f). The only other mammalian carnivore capable of climbing trees and depredating nests in the Doñana region is the common genet (*Genetta genetta*; body mass of around 2 kg). However, this species usually plucks and devours the nestlings within the nest or up the tree, and multiple evaluations by radio-tracking and intensive track-surveys through different decades have shown that genets are fully excluded from our study plot by the larger lynx^47,69,70^. Finally, lynx predation was concentrated on well-feathered nestlings (above 25–30 days of age). No predation was ever detected on younger nestlings in more than 13,000 h of photo-trapping at kite nests, or at hundreds of nests checked on pines, on whose trunks the claw-signs of lynx-climbing are easy to detect. Therefore, the presence of feathered nestlings plucked on the ground by a mammalian carnivore within about 10 m of the nesting tree represented the typical signature of lynx predation (Extended Data Fig. 3c–e). Furthermore, because lynx predation targeted large nestlings with much plumage and many long feathers, their remains were easy to spot and remained evident for long periods of at least four weeks (based on plucked nestlings from 28 nests checked every week; Extended Data Fig. 2e), which is much longer than the typical interval between our nest visits. Thus, the assessment of lynx predation was unlikely to suffer from detectability issues.

Rabbit abundance for our study plot was available since 2005 as part of standardized surveys of this species by the ‘Natural Processes Monitoring Team’ (ESPN, ICTS-RBD) of Doñana Biological Station -CSIC. Surveys were conducted each spring along a fixed 9.91 km transect driven slowly after dusk, when rabbits are most active^80^, using a 4 × 4 vehicle equipped with a handheld 100 watt spotlight. Each survey was performed by a team of at least two observers positioned on the roof of the vehicle at a height of at least 3 m above the ground to enhance visibility and improve sampling accuracy^81^. Rabbits were recorded by direct visual detection and confirmed with binoculars whenever necessary. The perpendicular distance from each rabbit to the transect line was measured using a laser telemeter (Geovid, Leica, Solms, Germany). The maximum width of the observation strip was 180 m, whereas the median detection distance was 25 m from the observers. Surveys were never conducted during adverse weather conditions capable of compromising visibility and data reliability, such as dense fog, rain, or strong winds. The rabbit counts of the surveys were then converted to rabbit densities using distance sampling analysis, following the methodology outlined by Buckland and co-workers^82^. This approach relies on a detection function, denoted as *g*(*x*) for linear transects, which is derived from distance data and used to estimate the probability of detection. To identify the model that best fitted the survey data, four models were evaluated: half-normal with cosine and hermite polynomial expansions, hazard rate with cosine and simple polynomial expansions, negative exponential with cosine expansion, and uniform with cosine expansion. In accordance with Buckland et al.^82^, the data were truncated by removing the 5% farthest observations to improve model robustness. The model with the lowest AIC value was then considered the best-fitting option.

Ducks (family Anatidae) are the main alternative prey for lynx during periods of rabbit scarcity^54,55^. To test whether the availability of ducks declined during drought, we accessed data from standardized aerial censuses of waterbirds, which are conducted monthly by experienced observers from the Natural Processes Monitoring Team (ESPN, ICTS-RBD) of Doñana Biological Station - CSIC using a light aircraft (for example, CESSNA high-wing)^83^. We extracted data for the National Park and averaged the monthly counts of ducks over the period February–May. We pooled data from all duck species (13 species), as we do not expect lynx to select specific duck taxa. Note that these censuses do not cover all ducks and waterbird species (for example, they miss secretive species that maintain cover even when flown over by the aircraft; refer to ref. ^83^ for details), but we believe that they provide coarse but reliable estimates of ducks’ availability for the lynx of our study. In 1997 the aerial surveys were only conducted in January. Therefore, for this year we estimated ducks’ abundance by the regression equation: “mean ducks’ abundance in February–May = 0.1944 × (ducks’ abundance in January) + 2761.4” (*R*^2^ = 0.69). Inclusion or exclusion of this year in the analyses did not change the results.

Kites in Doñana can reach very high densities (up to over 10 pairs per km^2^)^84^ and, even when their diet is dominated by rabbits, they will still feed on a large variety of alternative prey (more than 250 prey species in Doñana). Species of this kind, involved in large networks of interactions, are likely to affect other taxa^85^. Thus, to test the potential for lynx predation to benefit lower trophic levels through kite limitation, we surveyed the abundance of a lower-trophic level insectivore, the little owl (body mass of around 170 g). This small owl is preyed upon by kites (26 individuals recorded among kite nest remains) and we have directly witnessed kites attempting to capture little owls on three occasions (one of them successful). Little owl territories were censused by listening to territorial calls at dusk, night and dawn during ad hoc surveys conducted every 10–15 days throughout April–June, and by visiting all trees in the area in May–June. This owl is semi-diurnal and territorial individuals are easy to see perched near the nest during the breeding period. This, coupled with our almost daily presence in the plot at dawn and dusk while working on kites from March to July, made the little owl population basically under constant survey.

### Statistical analyses

**Extended Data Fig. 6:**
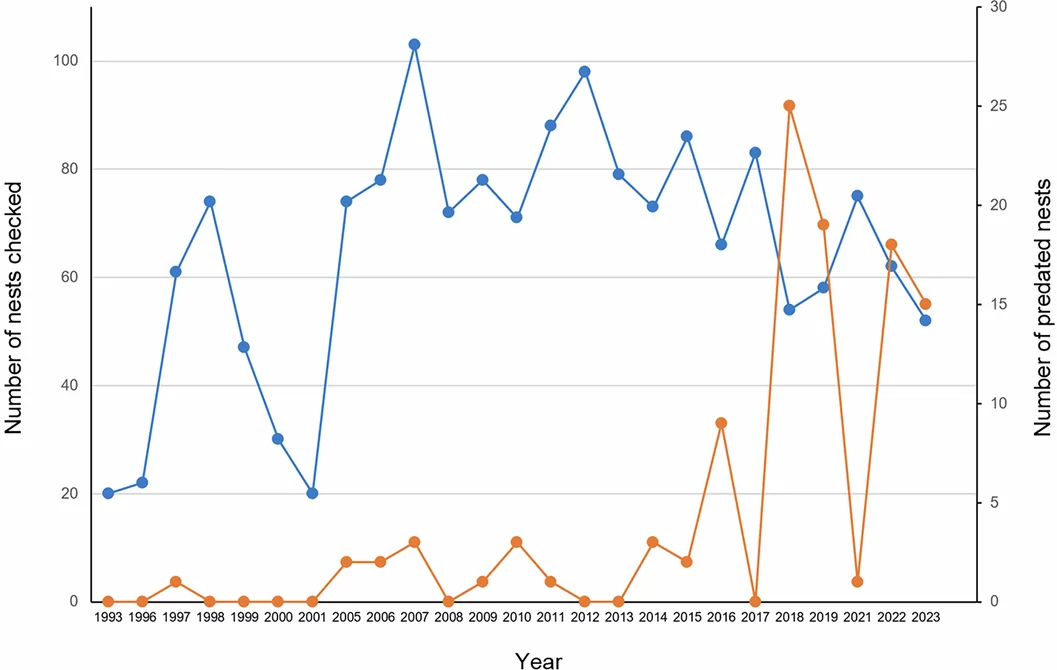
Total number of checked nests and predated nests for each of the 25 years of study.

We used a GLM^86^ with binomial errors and a logit link function to test whether annual lynx predation rate was related to rabbit availability, drought, or their additive or interactive effect. We also re-ran the same models substituting drought with climate anomalies. These models were systematically equivalent to those based on drought (Extended Data Table 2) and thus, to avoid redundancy, throughout the rest of the paper we refer exclusively to drought. The number of nests checked for predation (predated and not; see below) was added to the model as an offset (weight), so as to control for sample size differences among years and to estimate predation as an annual proportion. As lynx started to predate kite nestlings only when these were older and could predate them until they fledged (see Field procedures above), we expressed annual predation rate as the number of nests predated by lynx divided by the number of nests that (1) reached the stage of well-feathered nestlings and (2) were checked again when nestlings were already capable of flying. This allowed us to exclude all those nests that were not actually available for lynx to predate (for example, because they failed before reaching the stage targeted by lynx), or that could have been predated after our last nest check. This avoided artefacts caused by classifying as non-predated those broods that could have been predated if they had reached the appropriate age, or if they had been checked for the last time too early before fledging. To ensure adequate sampling, we only considered for analysis those years in which predation rates could be ascertained at a minimum of 20 nests. So filtered, predation rates were available for 25 years, based on 1,624 nests cumulatively checked during three decades (1993–2023) (*N* = 64.9 checked nests per year on average, with a range of 20–103 before 2013 and 52–86 after 2013; Extended Data Fig. 6).

Data on rabbit density were only available since 2005. For the preceding years, rabbit density was back-estimated by a regression between rabbit density and the percentage of rabbits by mass in the diet of black kites in the study area. We tested linear, quadratic and exponential relations, and found that the best fit (where AICc is over two units fewer than the next best model) was given by the exponential equation: rabbit density = 0.045928 × exp(5.90978 × rabbit incidence in kite diet) (*N* = 19 years, *R*^2^ = 0.81). To reinforce the solidity of the results, we re-ran the models of lynx predation rate with rabbit abundance estimated both as rabbit density as well as rabbit incidence in kite diet. Finally, we used a GLM^86^ with gaussian errors to confirm that ducks’ abundance declined in drought years. Although we have no data on annual lynx diet through the whole three decades of study, we cannot think of any ecological reason by which ducks should have stopped being the major alternative prey for lynx, as reported in the 1980s and 1990s^47,54,55^.

We hypothesized that certain characteristics of kite nests and broods could make them more susceptible to lynx predation. In particular, we hypothesized that: (1) lower nests could be easier for lynx to detect and to climb; (2) proximity to the marshes could affect the likelihood of predation, because of the higher availability of rabbits^47^ and alternative marshland prey; (3) larger kite broods could be at higher risk of detection and predation, because they could be associated with louder and more frequent begging for food, more excrements and prey remains on the ground below the nest, and a higher food reward for the predator; and (4) proximity to other nests predated by lynx could increase predation likelihood, as predation could be concentrated in core areas of lynx ranges, or in restricted areas used by an individual lynx during a bout of food scarcity. To test these ideas, for each of 108 nests predated by lynx through the years, we randomly selected a kite nest that successfully fledged young in the same year. We then used a binomial generalized linear mixed model (GLMM)^87^ with year as a random factor to test whether predated and non-predated nests differed in terms of nest height, brood size and distance to the marshes or to the nearest predated nest.

As lynxes are rabbit specialists, we further hypothesized that a lynx could be more likely to predate a brood if it had found rabbit remains on the ground under a kite nest. Destroying the brood of a kite pair that preys on rabbits could lower the prey requirements of a potential competitor, indirectly freeing rabbit-resources for lynx. We speculated that this could add an extra motivation on top of the nutritional benefits of consuming the kite nestlings. To test this idea, we used a *χ*^2^ test to compare the presence–absence of rabbits between the diet of kite pairs predated or not by lynx, using the same sample of the previous analysis. We conducted two separate analyses for the incidence of rabbits among prey remains on the ground under the nest and among prey remains recorded inside the nest. We also conducted these analyses twice, using an individual prey item or an individual nest as the sample unit. In many cases, no prey items were observed under the nest, but this was because thick vegetation impeded adequate detection (for example, nesting trees surrounded by large bramble thickets or tall grass and shrubs). Thus, to avoid inadvertent biases and make the test more conservative, we restrained the analysis to nests with sufficient ground visibility and where at least one prey item had been recorded under the nest. Note that all of the predated and non-predated nests of this analysis contained well-grown chicks of similar age at the time of diet assessment, thus discounting biases caused by comparing nests at different stages of chick development. To test the alternative hypothesis that lynx could have simply learnt to associate rabbit remains on the ground with a higher food reward in the nest, we used a *t*-test to compare the number of chicks in the nest and their cumulative body mass between nests with or without rabbit remains on the ground.

To test whether older kites were somehow better capable of avoiding lynx predation (for example, by safer nest placement), for each nest held by a known-age parent and predated by lynx in year X, we randomly selected a non-predated nest held by a known-age parent that successfully fledged young in year X (that is, was not predated) (*N* = 82 individuals). The range of parental ages available for analysis was 4–27 years old. We then compared the parental age of predated and non-predated nests by means of a binomial GLMM with year and the identity of each pair of matched parents as a random factor, so as to enforce a matched comparison.

To examine the potential fitness cost of lynx predation, we compared the longevity and lifetime reproductive success between individuals that suffered or not brood predation by lynx. Here, for each individual of age A in year X and that suffered lynx predation in year X, we chose an individual of age A in year X and that successfully fledged young in year X (*N* = 40 individuals). Whenever no individual of the same age was available in year X, we used an individual of age A + 1 or A – 1 and alternated between age increments and reductions in subsequent selections, so as to maintain a 0 net age-difference between predated and non-predated individuals. This allowed us to control for age and year when testing the impact of brood loss to lynx. Whenever fledgling production could not be ascertained (for example, for safety or logistical reasons), we estimated it as the mean number of young fledged by that individual in the four years before and after the interruption and converted it to an integer number. Longevity and lifetime reproductive success were compared between individuals that suffered or not lynx predation by a Poisson GLMM with the identity of each pair of matched individuals as a random factor, so as to enforce a matched comparison.

We investigated whether kites responded to lynx predation by emigrating to a new territory the following year (breeding dispersal). To this end, for each marked kite parent that suffered nest predation by lynx in year X and still alive in year X + 1, we chose a non-predated individual that successfully raised young in year X and was also still alive in year X + 1 (*N* = 62 individuals). The territory emigration rate of predated and non-predated individuals was compared by means of a binomial GLMM with the identity of each pair of matched individuals as a random factor. We also examined whether predated territories had a higher likelihood of remaining vacant (go extinct) the next year. Here, for each year, we randomly selected a number of non-predated nests equal to the number of predated nests and compared their territory extinction rates by means of a binomial GLMM with year as a random factor.

Finally, to test whether lynx predation rate could limit kite abundance and whether this could in turn favour lower-trophic level species, we related annual kite abundance to the previous year’s lynx predation rate and then the annual abundance of the insectivorous little owl to the abundance of kites and to intraguild predation in the previous year by means of Gaussian GLMs.

In all GLMs and GLMMs, an explanatory variable was considered important when it caused an increase of more than two AICc-units upon its removal from the maximal model, a significant increase in model deviance as determined by a likelihood ratio test, and when the 95% confidence intervals of its parameter estimates did not overlap zero^87^. Throughout, we assessed collinearity through VIF values, which were always <1.5 (ref. ^87^). Model assumptions were checked by looking at overdispersion, QQ plots, histograms of residuals, and plots of standardized and normalized residuals against fitted values and against explanatory variables. *R*^2^ for GLMMs were calculated according to ref. ^88^. All models were implemented in R v.3.6.2 (ref. ^89^); all tests were two-tailed, statistical significance was set to *α* < 0.05, and all means are given ±1 s.e.m.

### Reporting summary

Further information on research design is available in the Nature Portfolio Reporting Summary linked to this article.

## Online content

Any methods, additional references, Nature Portfolio reporting summaries, source data, extended data, supplementary information, acknowledgements, peer review information; details of author contributions and competing interests; and statements of data and code availability are available at https://doi.org/10.1038/s41586-026-11194-z.

## Supplementary information

Reporting Summary

Peer Review File

## Figures and Tables

**Extended Data Table 1:**
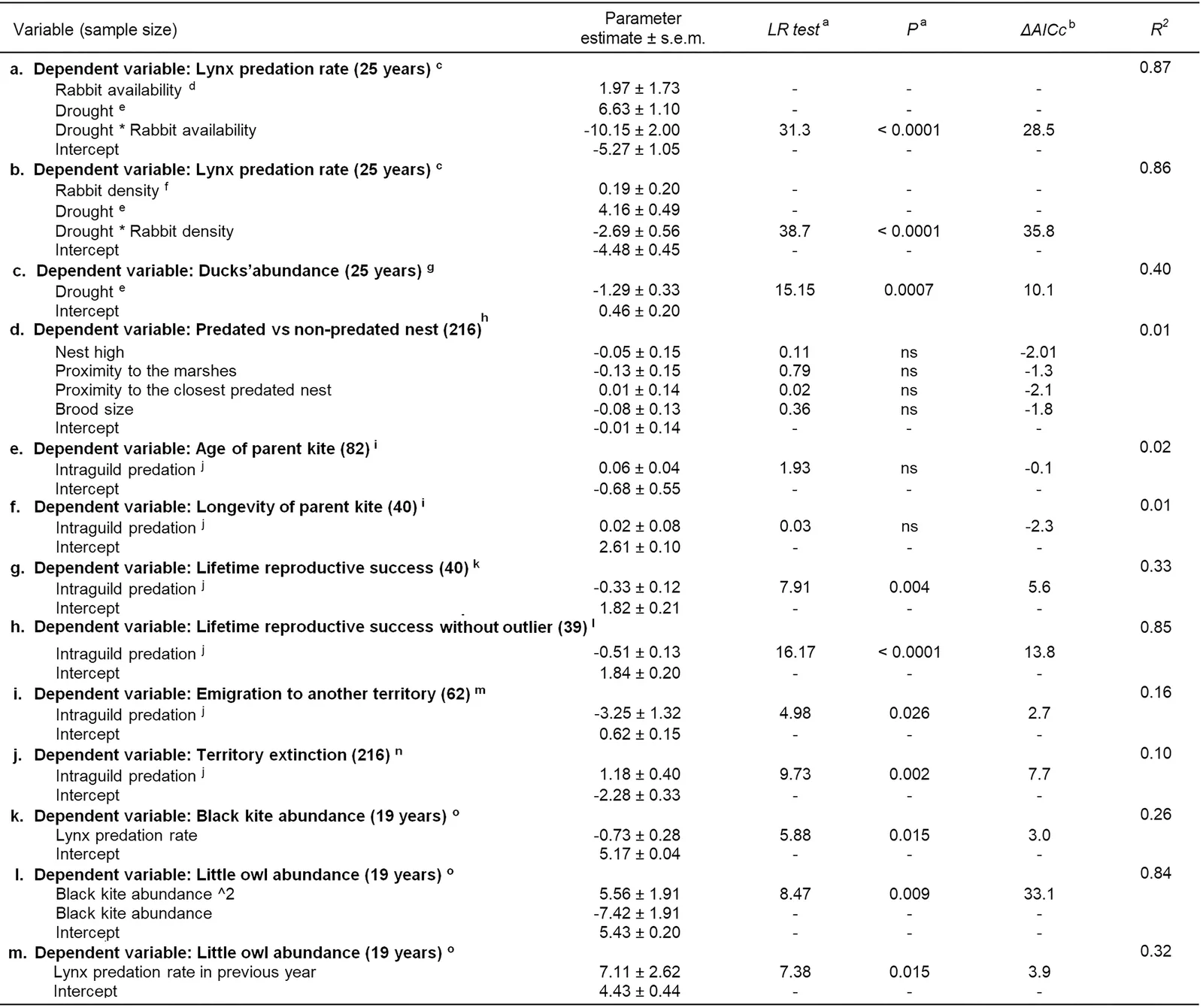
Models investigating the determinants of intraguild predation by lynx on black kites, their consequences for kite fitness, abundance, territory emigration and extinction, and their potential to cascade to lower trophic levels

^a^ Likelihood ratio test of the change in deviance upon removal of the variable from the model. We place the symbol “-” when P-values cannot be calculated (main effect of a variable already included in an interaction) or when they are not of interest (significance of the intercept).

^b^ ΔAICc is the change in AICc that would derive by the removal of the variable from the model. Negative values imply that the model improved if the variable was removed, while positive values imply that the model worsened if the variable was removed. We place the symbol “-” when AIC values cannot be calculated (main effect of a variable already included in an interaction) or when they are not of interest (significance of the intercept).

^c^ GLM with binomial errors and a logit link function for proportional data. Lynx predation rate was expressed as the number of kite broods predated by lynx divided by the number of broods that reached the stage of well-feathered nestlings (NBWF) and thus available for lynx predation (see Methods). NBWF was fitted to the GLM as a “weight”.

^d^ Rabbit availability was estimated as the proportion of rabbits in the diet of kites.

^e^ Categorical variable with two levels: 1 = year with normal (non-drought) conditions; 2 = year of drought.

^f^ Rabbit density was estimated by distance sampling along standardized survey-transects (see Methods).

^g^ GLM with gaussian errors. The abundance of ducks was estimated as the mean number of duck individuals counted during monthly aerial spring surveys (see Methods).

^h^ GLMM with binomial errors and a logit link function discriminating between predated nests and non-predated nests (see Methods). Year was fitted as a random term.

^i^ GLMM with Gaussian errors and an identity link function. The identity of each pair of matched parents (individual that suffered or not brood predation) was fitted as a random term (see Methods).

^j^ Factor variable discriminating between parental kites that suffered brood predation (coded as 1) and individuals that successfully raised fledglings (coded as 0).

^k^ GLMM with Poisson errors and a logarithmic link function. The identity of each pair of matched parents (individual that suffered or not brood predation) was fitted as a random term (see Methods). Lifetime reproductive success was the cumulative number of fledglings generated throughout life.

^l^ Same model as previous model “g” above, but after excluding an outlier individual with a value of lifetime reproductive success 20% higher than the next highest value.

^m^ GLMM with binomial errors and a logit link function discriminating between individuals that emigrated to another territory the following year or not. The identity of each pair of matched parents (individual that suffered or not brood predation) was fitted as a random term (see Methods).

^n^ GLMM with binomial errors and a logit link function discriminating between territories that remained vacant or not the following year (see Methods). Year was fitted as a random term.

^o^ GLM with Gaussian errors and an identity link function.

**Extended Data Table 2:**
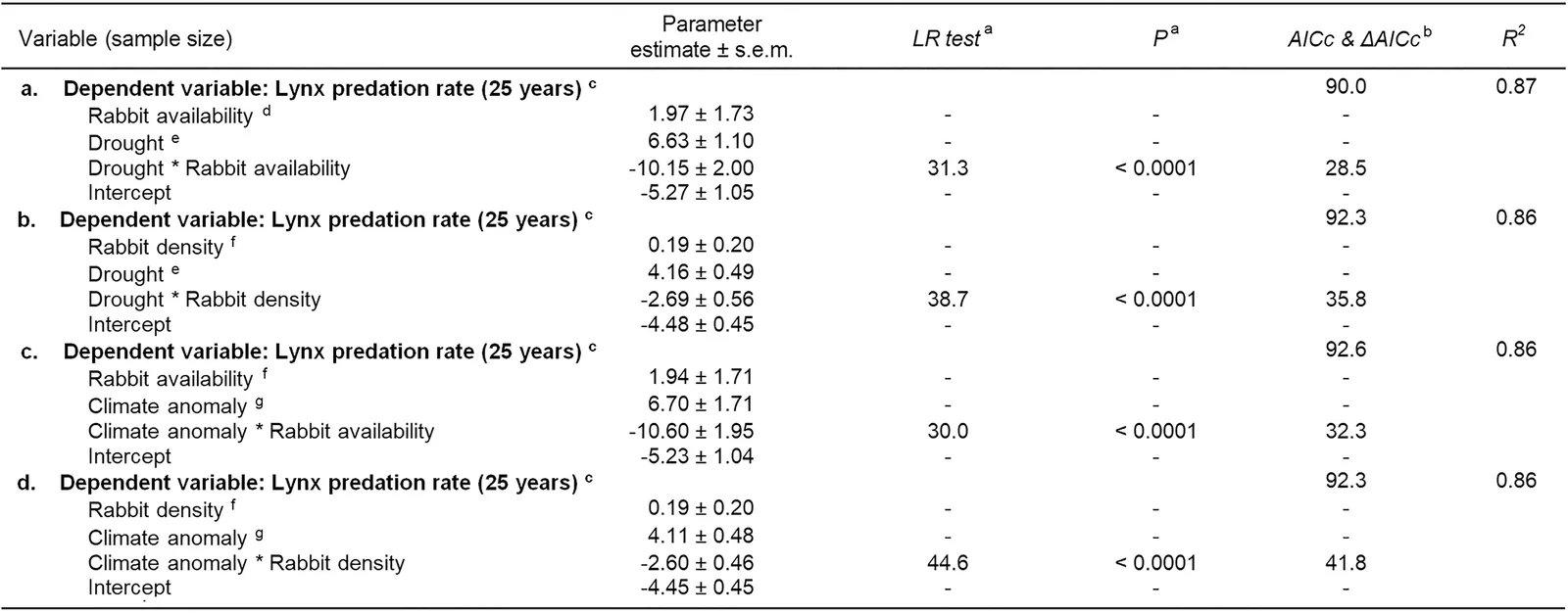
Models investigating the predation rate by lynx on black kites in relation to rabbit density, drought and climate anomalies (see Methods)

^a^ Likelihood ratio test of the change in deviance upon removal of the variable from the model. We place the symbol “-” when P-values cannot be calculated (main effect of a variable already included in an interaction) or when they are not of interest (significance of the intercept).

^b^ The first line of each model reports the AICc of the whole model, while the line of each explanatory variable reports the change in AICc-units that would derive by the removal of the variable from the model (ΔAICc). Negative values imply that the model improved if the variable was removed, while positive values imply that the model worsened if the variable was removed. We place the symbol “-” when AIC values cannot be calculated (main effect of a variable already included in an interaction) or when they are not of interest (significance of the intercept).

^c^ GLM with binomial errors and a logit link function for proportional data. Lynx predation rate was expressed as the number of kite broods predated by lynx divided by the number of broods that reached the stage of well-feathered nestlings (NBWF) and thus available for lynx predation (see Methods). NBWF was fitted to the GLM as a “weight”.

^d^ Rabbit availability was estimated as the proportion of rabbits in the diet of kites.

^e^ Categorical variable with two levels: 1 = year with normal (non-drought) conditions; 2 = year of drought.

^f^ Rabbit density was estimated by distance sampling along standardized survey-transects (see Methods).

^g^ A year was classified as a “climate anomaly” if it was classified as drought, or experienced extreme temperatures, or extreme rainfall in spring (see Methods).

## Data Availability

The data generated in this study are available at https://doi.org/10.20350/digitalCSIC/28994.
