# Supplementary material for: Drought and disease trigger novel interactions among top predators
Source: Nature. 2026 Oct 21;659(8139):121–7. doi: 10.1038/s41586-026-11194-z (PMC13736497; doi:10.1038/s41586-026-11194-z)
Supplement: Supplementary file 1 [file 41586_2026_11194_MOESM1_ESM.pdf]

nature portfolio

Corresponding author(s): Fabrizio Sergio

Last updated by author(s): 09/01/2026

Reporting Summary

Nature Portfolio wishes to improve the reproducibility of the work that we publish. This form provides structure for consistency and transparency in reporting. For further information on Nature Portfolio policies, see our Editorial Policies and the Editorial Policy Checklist.

Statistics

For all statistical analyses, confirm that the following items are present in the figure legend, table legend, main text, or Methods section.

n/a	Confirmed	
☐	☒	The exact sample size (*n*) for each experimental group/condition, given as a discrete number and unit of measurement
☐	☒	A statement on whether measurements were taken from distinct samples or whether the same sample was measured repeatedly
☐	☒	The statistical test(s) used AND whether they are one- or two-sided<br>*Only common tests should be described solely by name; describe more complex techniques in the Methods section.*
☐	☒	A description of all covariates tested
☐	☒	A description of any assumptions or corrections, such as tests of normality and adjustment for multiple comparisons
☐	☒	A full description of the statistical parameters including central tendency (e.g. means) or other basic estimates (e.g. regression coefficient) AND variation (e.g. standard deviation) or associated estimates of uncertainty (e.g. confidence intervals)
☐	☒	For null hypothesis testing, the test statistic (e.g. *F*, *t*, *r*) with confidence intervals, effect sizes, degrees of freedom and *P* value noted<br>*Give P values as exact values whenever suitable.*
☒	☐	For Bayesian analysis, information on the choice of priors and Markov chain Monte Carlo settings
☒	☐	For hierarchical and complex designs, identification of the appropriate level for tests and full reporting of outcomes
☐	☒	Estimates of effect sizes (e.g. Cohen's *d*, Pearson's *r*), indicating how they were calculated

Our web collection on statistics for biologists contains articles on many of the points above.

Software and code

Policy information about availability of computer code

Data collection	n/a
Data analysis	R.3.6.2

For manuscripts utilizing custom algorithms or software that are central to the research but not yet described in published literature, software must be made available to editors and reviewers. We strongly encourage code deposition in a community repository (e.g. GitHub). See the Nature Portfolio guidelines for submitting code & software for further information.

Data

Policy information about availability of data

All manuscripts must include a data availability statement. This statement should provide the following information, where applicable:

- Accession codes, unique identifiers, or web links for publicly available datasets
- A description of any restrictions on data availability
- For clinical datasets or third party data, please ensure that the statement adheres to our policy

The datasets generated and analysed during the study will be available in a public repository upon publication

Research involving human participants, their data, or biological material

Policy information about studies with human participants or human data. See also policy information about sex, gender (identity/presentation), and sexual orientation and race, ethnicity and racism.

Reporting on sex and gender	n/a
Reporting on race, ethnicity, or other socially relevant groupings	n/a
Population characteristics	n/a
Recruitment	n/a
Ethics oversight	n/a

Note that full information on the approval of the study protocol must also be provided in the manuscript.

Field-specific reporting

Please select the one below that is the best fit for your research. If you are not sure, read the appropriate sections before making your selection.

☐ Life sciences ☐ Behavioural & social sciences ☒ Ecological, evolutionary & environmental sciences

For a reference copy of the document with all sections, see nature.com/documents/nr-reporting-summary-flat.pdf

Ecological, evolutionary & environmental sciences study design

All studies must disclose on these points even when the disclosure is negative.

Study description	Analysis of intraguild predation between a mammalian carnivore and a raptorial bird in relation to climate change
Research sample	Black kite population of Doñana National Park. This is a unique population where all ages of a very long-lived bird can be sampled, allowing unique insight into how life history traits can vary with age from recently born individuals to 27 years old ones
Sampling strategy	The sample is based on over 1600 nests monitored over three decades, which allowed to investigate the determinants and fitness consequences of intraguild predation. This is a very large sample size for studies on raptorial birds
Data collection	Data on nest monitoring and intraguild predation were collected as part of a long-term demographic study of this population. Field data were collected by all co-authors
Timing and spatial scale	Data were collected between 1993 and 2024 in a 10 km2 plot located in Doñana National Park (southwestern Spain)
Data exclusions	Years with less than 20 monitored nests were excluded, because less than 20 nests was considered inadequate to estimate the rate of rare intraguild predation events. An analysis is shown with and without an outlier datapoint
Reproducibility	n/a
Randomization	n/a
Blinding	n/a

Did the study involve field work? ☒ Yes ☐ No

Field work, collection and transport

Field conditions	Fieldwork was implemented in Doñana National Park (south-western Spain), during the period February-July. During spells of hot weather, fieldwork was confined to early morning and later afternoon, usually with temperatures of 10-27 degrees C and no rain
Location	Doñana National Park (36°56′51″N 6°21′31″O). Elevation = 1-33 m a.s.l
Access & import/export	All fieldwork complied with the regulations issued in the permits emitted by Doñana National Park, which include instructions on prohibited fieldwork activitities, as also discussed in ad hoc reunions with the Park Authority. All fieldwork activities followed the bioethical procedures established in the relevant bio-ethical permits

Disturbance	Nests were accessed and checked quickly. Nest checks usually lasted less than 5 minutes, while processing of nestlings for ringing usually involved visits of 20-30 minutes, as outinely conducted for decades with no adverse effects on the population. All field methodologies have been approved multiple times by professional bioethics committees and the impact of al research activities has been quantitatively evaluated by comparison with control individuals (e.g. Sergio, F. et al. 2015. No effect of satellite tagging on survival, recruitment, longevity, productivity and social dominance of a raptor, and the provisioning and condition of its offspring. J. Appl. Ecol. 52, 1665–1675).

Reporting for specific materials, systems and methods

We require information from authors about some types of materials, experimental systems and methods used in many studies. Here, indicate whether each material, system or method listed is relevant to your study. If you are not sure if a list item applies to your research, read the appropriate section before selecting a response.

Materials & experimental systems

n/a	Involved in the study
☒	☐ Antibodies
☒	☐ Eukaryotic cell lines
☒	☐ Palaeontology and archaeology
☐	☒ Animals and other organisms
☒	☐ Clinical data
☒	☐ Dual use research of concern
☒	☐ Plants

Methods

n/a	Involved in the study
☒	☐ ChIP-seq
☒	☐ Flow cytometry
☒	☐ MRI-based neuroimaging

Animals and other research organisms

Policy information about studies involving animals; ARRIVE guidelines recommended for reporting animal research, and Sex and Gender in Research

Laboratory animals	n/a
Wild animals	Nestlings Black kites (Milvus migrans) were routinely ringed and immediately returned to their nest as part of a long-term demographic study of the population. No animals were killed
Reporting on sex	n/a
Field-collected samples	The study did not involve laboratory samples collected in the field.
Ethics oversight	The study was conducted in strict accordance with the national and European laws concerning the use of animals for scientific purposes (protocols EBD-11/09 and EBD-11/25 approved by the Animal Care and Bio-Ethics Sub-Committee of the CSIC)

Note that full information on the approval of the study protocol must also be provided in the manuscript.

Plants

Seed stocks	n/a
Novel plant genotypes	n/a
Authentication	n/a
