# Supplementary material for: Drought and disease trigger novel interactions among top predators
Source: Nature. 2026 Oct 21;659(8139):121–7. doi: 10.1038/s41586-026-11194-z (PMC13736497; doi:10.1038/s41586-026-11194-z)
Supplement: Supplementary file 2 [file 41586_2026_11194_MOESM2_ESM.pdf]

nature portfolio

Peer Review File

Drought and disease trigger novel interactions among top predators

Corresponding Author: Dr Fabrizio Sergio

Any redactions in this file are there to maintain patient confidentiality, the confidentiality of unpublished data, or to remove third-party material.

This file contains all reviewer reports in order by version, followed by all author rebuttals in order by version.

Version 0:

Reviewer comments:

Referee #1

(Remarks to the Author)
GENERAL COMMENTS

Overview & Summary
This study is based on detailed monitoring of black kite nests over 30 years in southwestern Spain (n = 1662 total nests with individual level marking and monitoring). This exceptionally long-term research program has enabled the authors to document a series of major ecological changes over time.

The study describes shifts in ecological interactions arising from an interaction between two major human perturbations, which have reshaped the ecology of Doñana National Park (PND). PND is a globally significant protected area near the Strait of Gibraltar that hosts a unique fauna with Eurasian and African elements. Around 2012, a new variant of rabbit hemorrhagic disease (RHD) spilled out from a rabbit farm in France, leading to severe rabbit declines in PND. Rabbits are the near-exclusive prey for the globally threatened Iberian lynx, historically a strict rabbit specialist. At least 16 other species eat rabbit in PND, 8 of which (wildcat, red fox, Egyptian mongoose, imperial and booted eagle, marsh harrier, red and black kite) prey heavily on rabbits (≥15% of diet; Villafuerte & Moreno 1997 Acta Theriol.). When rabbits declined, the PND lynx population did not crash, as one might have expected based on the textbook-famous population cycles of Canada lynx and snowshoe hare. Lynx instead broadened their prey base, including frequent depredation of juvenile black kites, which had previously an exceedingly rare occurrence. This significantly reduced chick survival, prompted many adults to abandon their territories, and contributed to a decline in kite population density. Kite decline, in turn, appears to have released the little owl (a predator of invertebrates) from kite predation and a consequent increase in owl numbers.

The authors contextualize this study as an example of intraguild predation (IGP, which is a combination of competition and predation where potential competitors eat each other: Polis et al. 1989 AREES) that emerged as a consequence of human activity and propagated to species at lower trophic levels (little owls). Ecologists' interest in IGP dates to the late 1980s, with work showing that IGP is commonplace in nature but was not accounted for in theoretical models. Investigators continue to study IGP, which is important in diverse ecological contexts. IGP is central, for example, to the interactions between apex and meso carnivores (e.g., Prugh & Sivy 2020 Ecol. Lett., Prugh et al. 2023 Science). While there is nothing unusual about IGP, this study adds a provocative proposition: Lynx predation was strongly associated with the frequency and quantity of rabbit remains occurring on the ground under kite nests. The authors interpret this as evidence of lynx 'punishing' kites for competition.

Strengths
Such long-term studies of wild populations are extremely rare and inordinately valuable – dynamics such as those documented here can only be captured via long-term study, making this a great example of why it is important to sustain and fund long-term ecological research. The chain of events described is totally captivating, and it is also instructive about the kinds of dynamics that accompany major environmental changes caused by human activity. The story is compelling for a general audience. Non-ecologists and non-scientists are fascinated by complex chains of species interactions and unexpected animal behaviors, as evidenced by the fact such papers are often covered in popular media.

The methods reported are sound. I am convinced by the fieldwork methods as described in the ms for (a) the long-term kite census of individually banded birds; (b) the authors' approach to inferring lynx-on-kite predation events; and (c) the distance sampling approach used to quantify rabbit densities. The value of the intensive long-term study of the kite population (and in PND, an unusually well stuied system) cannot be stressed enough. I am persuaded by the general approach to statistical modeling, which entailed fitting generalized linear models and mixed-effects models; that is the same general approach I would have used.

Weaknesses
One weakness of the current submission is that while the natural history is unambiguously cool, the explanation of its scientific significance is unsatisfying. In in the Summary Statement (abstract), the authors use ecology jargon in ways that will (a) be confusing to non-ecologists and (b) unintentionally makes the study seem narrower and less significant than it might be. Moreover, the concluding paragraph, leading with: "Forecasting the biodiversity effect of climate change will be challenging given the complex, interactive, context-dependent, unexpected and novel dynamics observed in even in our small trophic module" is downright depressing. It implies that no major advance has been made in this study, as we are already all-too-well aware that ecology is complicated and that forecasting ecological dynamics is tricky. However, I think the authors are overlooking a theme that unifies their results with a larger literature, which might help them contextualize the results in a more convincing and productive way.

A bigger issue is that the manuscript does not (yet) clear my bar for rigor of inference and interpretation. While I find no problem with the data or the general analytical strategy, I have multiple concerns about the authors' decisions about which data to show, how to show those data, and how much can be inferred or insinuated based on the data presented. These concerns vary in seriousness. Some are minor (e.g., the authors use a number of terms and phrases in the text that I think are poorly chosen or not empirically justified); some are medium (e.g., the authors appear to selectively cite evidence from prior work in ways that are maximally convenient for their interpretation); and some are potentially serious but impossible for me to evaluate (e.g., the analyses presented, while none of them 'wrong', do not directly or maximally test the inferred chain of events). My biggest concern is that there are a lot of relevant data that are not presented and potential corroborating analyses that appear not to have been conducted, and some of the decisions about how to present the data are counterintuitive, which leaves many questions unanswered. There is plenty of room in the manuscript and extended data for more information and analysis, so concerns about brevity and word/figure limits are not a valid excuse for omitting these. Not all of my concerns will necessarily require additions to the manuscript; in some cases, they may only require clarification (but in such cases, I would like to see supporting information or justification at least in the response letter).

Altogether, this set of factors leaves me with the uneasy feeling that there may be more to this story than the current presentation reveals – or perhaps some inconvenient issues that the authors wish to avoid having to explain. This hunch may be wrong; and even if it isn't wrong, the authors may be able to clarify all issues in the revision and responses. I organize my comments by number below, as some are long.

SPECIFIC QUESTIONS & CONCERNS

(1) Motivation and significance statements
Question for the authors: What is the potentially generalizable advance in our understanding of how interacting anthropogenic stressors influence ecological communities?

The ms articulates one very good general motivation for this study, namely: The most critical effects of climate change will not be predictable based solely on the weather and species' physiological tolerance. Instead, effects will be mediated by species interactions, which makes them harder to predict (L32-47). From that idea, however, the authors proceed directly into IGP (L42), and thence to successively narrower motivations: the limited understanding of IGP, and the small number of IGP studies that involve "killings among taxonomically distant predators" (unclear why this matters). The synopsis of 'study implications' on L24-26 states: "These results provide the first demonstration of a climate-induced intraguild predation impact that amplifies multiple global change drivers in vertebrate communities." This is the jargon noted above that, ironically, makes the study seem narrow despite the fancy-sounding words (one theory: the profundity of any study is inversely proportional to the number of priority claims and puffed-up words.)

But I suspect this was merely a miscalculation by the authors, and that more can be done to link this study tangibly to a broader literature about behavioral / trophic plasticity and its implications. Specifically: Drought is one common source of food limitation, but there are others, including other types of extreme weather that projected to become more frequent and/or intense with climate change (heat, cold, flood, storm) along with other kinds of human impact (overhunting, environmental pollution, land-use effects, introduction of nonnative species and pathogens). A more inclusive framework might be the tendency of stressors (and their interactions) to induce extreme dietary plasticity and strong direct and indirect effects.
Example A: Major tropical cyclone causes food limitation, spatial redistribution, and shifts in ungulate and carnivore communities (Walker et al. 2023 Nature; ttps://doi.org/10.1038/s41586-023-06722-0).
Example B: Depletion of native prey intensifies predation on livestock by carnivores, which in turn leads to retaliation killings by pastoralists (Woodroffe et al., 2000-2005: e.g. https://doi.org/10.1111/j.1469-1795.2000.tb00241.x). In these and other examples, a primary impact of human activities is depletion of prey abundance, which has alternative potential outcomes: The predator population might (a) collapse or fall into conflict with humans (as many observers have feared / assumed), or (b) exhibit resilience via previously undocumented behavioral plasticity and food-web rewiring (brightening the prognosis?).

Recent work using new technology and/or clever fieldwork suggests that the latter scenario is common, whether in response

to largely natural or highly anthropogenic perturbations.
Example C: Collapse of Sitka deer population in Alaska caused wolf (putative deer specialists) to switch to sea otter, a totally unexpected prey (Roeffler et al. PNAS: https://doi.org/10.1073/pnas.2209037120).
Example D: Pumasin Patagonia (putative deer specialists) become penguin specialists to subsist in a new national park (Serota et al. 2025 PRSB: https://doi.org/10.1098/rspb.2025.2172).
Example E: Jaguars in Costa Rica (putative large-mammal specialists) are heavily subsidized by sea turtles (https://doi.org/10.4013/nbc.2017.121.01).

These phenomena connect to fundamental problems in ecology and conservation: (a) forecasting impacts on food-web structure, community stability, and species-of-concern; (b) distinguishing between realized vs. fundamental niche breadth; (c) whether such plasticity is a sufficient to buffer against human activity; (d) frequency and extent of food-web rewiring. The authors vaguely allude to this idea on line 177-179 but then revert back to IGP-specific considerations, including the argument that "IGP will require much more consideration in climate change research and assessments" – an unlikely prospect. The focus on lynx is also fortuitous in this regard, because it can be set up against the famous Canadian lynx-hare dynamics, which suggests an outcome different from that in this study.

This framework can be incorporated more effectively by adding a new paragraph in between the first and third of the current Main Text, as a bridge between the key idea that "biotic interactions modulate climate effects" and the more specific issue that "IGP is poorly understood". Both of those are still relevant, but IGP then becomes a specific manifestation of the more general problem of (a) shifts in niche breadth in response to anthropogenic stressors and (b) the population / community consequences of those shifts. Later in the article (and in the Summary Paragraph), this framework can also be used to more effortlessly and convincingly explain the how this unique long-term study enriches our growing understanding of an emerging shift in our understanding of what resilience can look like and what ecological byproducts it produces. The same framework also suggests targets for future work (points a-d in the prior paragraph), which are not clearly articulated in the current submission, but should be. A paragraph along these lines would be a fitting replacement for the rather generic, discouraging, and in my opinion outdated views offered in the current concluding paragraph. It would also fit well with a shift towards more scientific neutral language about the biodiversity and climate "crises," which I recommend below.

(2) THE 'PUNISHMENT' INTERPRETATION
[2a] Plausibility of this intepretation
The authors strain to interpret the 'rabbit-remains under nests' result as evidence that lynx are 'punishing' kites for eating rabbits. This is stated in the Summary Paragraph (L20), Main Text (L132), and Methods (L647). The wording used ("consistent with") subtly telegraphs that the authors are aware that this interpretation is a stretch; conspicuously, this particular interpretation is also left out of the conclusions, probably for the same reason. Yet, the way it is presented elsewhere, without any mention of plausible alternative interpretations, will give casual readers the impression that it has been conclusively shown.

As far as I know, the authors' interpretation is technically possible -- it certainly makes ecological sense to kill off a competitor during lean years, and one could fabricate evolutionary just-so stories about how lynx might have gained this tendency. But unlike when apex carnivores kill mesocarnivores that scavenge their kills (interference competition, a direct interaction), this idea requires lynx to cognitively process that the presence of rabbit remains under a nest == a competitor (exploitation competition, an indirect interaction and a more abstract concept). I don't know if lynx are capable of that; but I do know that even university students often fail grasp the distinction between interference and exploitation competition. Stranger things are true, but the burden of proof is to show it experimentally; one cannot assume it.

The plausible (probable) alternative hypothesis that are that rabbit bits / scents are simply strong cues that put lynx into hunting mode – either because rabbit smells like food and lynx want to find it, or because lynx have learned to associate rabbit on the ground with food in the tree. That would be a sufficient explanation and does not entail any association between kites and competition. I see no basis in the paper for excluding this hypothesis in favor of the one offered in the ms, and without knowing more about lynx brains, I would default to the simpler interpretation.

I think that the authors are justified in briefly introducing their idea in the latter portion of the main text, but they should very clearly label it as a hypothesis to avoid any possibility of readers misinterpreting it, and they should pair it with some alternative hypothesis (such as I suggest above). Expressing it as a 'result' in the Summary Paragraph and text (L136: "novel insight on the mechanisms and motivations driving IGP") is not defensible, even with the authors' subtle verbal qualification ("consistent with").

[2b] Presentation of these results
As a separate but related issue, I find the data presentation in Fig. 3a,b,e non-intuitive, confusing, and poorly explained. While writing the 'overview' above, I found that it was actually hard to put the result (as currently shown) into words. For starters, what exactly are those bars? For panel (a), where the rabbit variable is a continuous proportion, my instinct is that bars should be Mean (± SE) [or boxplots] % of rabbit prey across nests in each category. Even better (and possibly complementarily), the analysis would be a logistic regression with probability of lynx predation on the Y-axis as functions of % rabbit prey on the ground / in the nest, visualized with curves fit and with 'rugs' on the top and bottom X-axes to show the distribution of data points. This might require 2 panels, which would be fine. The authors might have some statistical argument against performing logistic regression given the nature of their data; that might be a reason not to include it in the manuscript, but I would still want to see it in a response letter.

Panels (3b) and (e) are more clearly contingency table analyses with pairs of binary categorical variables. Even in those

panels, it is much more intuitive to have '% of nests depredated' on the Y axis, bar colors corresponding to +/- Rabbit, and bar groups for 'Ground' and 'Nest'. This would more clearly reflect the goal, which is to test the predictors lynx predation (dependent variable is conventionally on the Y-axis).

For all data figures (main text and ED), I'd like to see essential statistics in the figure captions, regardless of whether they are also in the text or not. Being able to see them in the same glance as the figures is really very helpful for reader comprehension. ED Table 1 has full model results for most of the models, which is good (it does not include the chi-squared tests for Fig. 3 which are only in the text: L128-131). Still, please add them in the captions (or the panels, if that is allowable and convenient). This is a very small redundancy in the scheme of things and it is more than justified by how much easier it makes it to understand the results, especially for casual readers. For chi-squared tests in Fig. 3, it is easy to include the full stats in the caption; for GLM results, report parameter estimates and P-values (or even just P-values) and include callouts to ED Table 1.

The statement of these results on L130-131 states "there was no such difference for prey remains observed inside the nest" but then states what appears to be statistical evidence that there are differences (P = 0.035; P = 0.056). Based on the chi-sq values reported, these P-values must be wrong (for a 2x2 contingency table, I get P = 0.75 and 0.56, respectively[?]). I assume that these are just typos in the P-values, because the chi-squared values match the figures, but you should re-check everything.

Finally: Red/orange and green is a poor color combination for colorblind readers. Even for those of us who see color, it is not pretty.

(3) UNANSWERED QUESTIONS AND 'MISSING' DATA/ANALYSES
Given the complexity of ecological dynamics under investigation (as the authors are the first to point out, L204), the manuscript is remarkably brief and sparing with display items in both the main-text (1 set of photos, 2 data figures) and extended data (1 map, 1 more set of photos, 1 data figure, 1 statistical table). In my view, the ratio of complexity to information content is very high. There would be no problem with presenting just 3 data figures, 1 table, and 200 lines of manuscript text if that were sufficient to establish the validity of the findings beyond reasonable doubt. However, I am left with a number of reasonable doubts and questions about issues that I think call for additional data and/or sensitivity analyses and/or discussion in the text. I do not think that any of them is unfair; they are all relevant to the reader's ability to gauge the validity of the inferences. The authors may not be able to address all of them, but they certainly can shed light on many of them.

[3a] Waterfowl data and temporal dimension. Time plays a critical role in the authors' account, which is effectively divided into 'pre-RHD' (before 2013) and 'post-RHD' (2013 onwards) intervals. The claim is that lynx-kite predation occurred only after RHD, and then only in drought years when rabbit densities dropped to very low levels. This is ostensibly because in non-drought years, lynx were able to prey on ducks and geese (L110), whereas in drought years waterfowl were unavailable, leading to kite predation. This is based on studies conducted from 1973 to 1984, showing that mallards were the commonest prey after rabbits in the 1970s (Delibes 1980), and that consumption of ducks and geese (and birds in general) increased after a "severe drought" in 1982-83 (Beltrán & Delibes 1991). Notably, despite this severe drought, waterfowl were still clearly available to lynx. Beltrán & Delibes noted that the graylag goose population had increased from 3000 to 85000 between 1973 and 1984, list land-use change as a contributing factor, and – remarkably – seem to have anticipated the events in this study: "The lynx is capable of occasionally shifting its predation to suitable alternative prey so that in case of need (e.g., if rabbits become excessively scarce for a short period after an RHD epidemic)…"

Why this matters: The assumed role of waterfowl in lynx diets – and their lower availability during drought -- is crucial to the interpretation about drought effects because otherwise it is unclear why lynx did not prey on kites in non-drought years when rabbit abundance was as low or even lower than in the drought years. Yet no information is presented about waterfowl abundance, and the data cited are from ~50 years ago. Beltrán & Delibes suggest that waterfowl are actually more accessible during drought years, because they are weakened and spend more time in pastures, and because lynx are better able to access the wetland area (contrary to the interpretation implied in the study). de Felipe et al 2024 GCB present extensive data on waterbird abundance in Guadalquivir Marshes and show a sustained and dramatic decline of both herbivores (gray geese) and pre-Saharan dabbling granivores (mallard) from 1985 to 2021, which is attributed to "deterioration of the Doñana National Park natural marshes" and is not obviously consistent with the idea that waterbird availability waxes and wanes with drought. The availability of these data make the lack of any discussion of waterfowl abundance puzzling. Data on lynx movements, if available, would also be helpful for evaluating the plausibility of inferred role of waterfowl. As would any concrete evidence, from scat or observations, that lynx are preying on waterfowl in wet post-RHD years but not in dry post-RHD years.

Further, given these temporal trends and the key temporal dimensions of this work (the pre/post RHD epidemic, the inter-annual variability in drought), it seems necessary to see the data presented through time: What are the trends in rabbit density, waterfowl abundance, and drought across the study period (or from whenever the relevant data or representative proxies are available). There is no doubt that lynx predation on kites has become more frequent, but the authors present a nuanced interpretation, and temporally explicit trends over time are critical supporting evidence for that interpretation…or they may complicate the interpretation, requiring it to be revised.

[3b] Categorization of 'drought' and temporal dimension (again). The authors categorize drought "as years in which inundation levels went below 20% of maximum flooding in February-March" (L489). Categorizing a potentially continuous variable is always a questionable decision, as information is lost and arbitrary criteria are required. In Fig. 2 we are shown

seemingly very compelling trends. These are driven by 5 drought years when rabbit abundance was low; all other drought and nondrought years alike had low kite predation, regardless of rabbit abundance. Which years these were is not specified.

Why it matters: While the R2 of these fits is high, these are still relatively small numbers of drought years. The numbers are passable given that these represent entire years of a 25+ years study. The worrying possibility is that these very tight trends are strongly influenced by the (arbitrary) criterion for classifying drought years (which again, are not show to be tied to the putative mechanism of alternative prey availability for lynx). A more convincing analysis, to me, would be to show that these trends are robust to analysis either by the abundance of waterbird prey OR by a quantitative version of the drought variable – e.g., actual % inundation instead of a threshold, or by rainfall. And again, showing trends in by year would be a highly informative complement. One possibility suggested by language and data in de Felipe et al. 2024 GCB, and by other information elsewhere (some mentioned below) is that data reflect a more general breakdown of the historical status quo in this ecosystem, influenced also by land-use change and agricultural water diversion (mentioned in the ms) in addition to RHD and climate change and perhaps other factors, which is not the interpretation the authors have proposed.

[3c] Causal inference on the relationship between lynx predation and kite decline, and kite decline and owl increase (and temporal dimension, again). The study concludes not just a set of pairwise interactions, but a dynamic chain of interlinked (interlynxed?) events that implies a temporal sequence: First, rabbit crash; then lynx (sporadically) depredate kites; then kites decline; then little owls are released. The authors' interpretation suggests that we should see high rabbit abundance prior to 2013, followed by a decline of rabbit abundance, with intermittent droughts leading to both further reductions in rabbit abundance and reductions in waterfowl occupancy and consequent spikes in kite predation, followed by increased little owl abundance. And we should see this whether drought is categorized by threshold or shown as a continuous inundation or rainfall variable. As with the data on lynx-kite predation ~ rabbit abundance, a correlation is shown between kite abundance and owl abundance (Fig. 3f) that looks very convincing. But again, the temporal trend in this correlation is important.

The statistical analyses (ED Table 1) test each response as a function of the putative proximal variable. For owls, this is black kite abundance, and strong support is found for a quadratic decreasing relationship (R2=0.84). But the interpretation supposes that the trends in little owls are ultimately a function of IGP by lynx: IGP is shown to predict kite variables, and kite abundance is shown to predict owls, but does IGP predict little owl abundance, as the interpretation suggests? The answer is not obvious, because the explanatory power of lynx predation on black kite abundance is relatively low (R2 = 0.26).

In this regard, there is reason to wonder about the influence of factors not mentioned in the text. Land use and water diversion are obliquely mentioned in the text. Another factor is not addressed, which is recent work led by the lead author showing that a chemical spill in the late 1990s led to elevated levels of lead, arsenic, and other toxic metals, with very severe effects on kite reproductive success as of the early 2000s. Although that was a long time ago, it is also emphasized that kites are long-lived species (30 years). So while many of the analyses in this study are effectively controlled by comparisons between black kites in the contemporary ecosystem (presumably exposed to similar environmental effects), the kite population declines mentioned in the text could plausibly relate to bioaccumulation of toxins, rather than lynx predation alone, in which case that might partially also explain the trend in little owls (rather than lynx IGP). By the authors' interpretation, we should see kite abundance fluctuating up and down through time (not just decreasing monotonically), and little owls should fluctuate in concert (not just monotonically increasing).

Finally under this point, data on kite diets other than rabbits would be good to know. Is it possible that black kite abundance is also regulated bottom-up by the availability of prey, rather that purely lynx IGP as implied by the analysis in ED Table 1? Such data would be even more valuable given that kite diets are described inconsistently. In three separate places, using almost identical language, it is said that black kite "specializes on rabbits where these are abundant" (L75, L510, L524). This is based on two studies from Matas Gordas (I am not familiar with the location, but it is either inside or just north of PND, based on references 38, 61) and a third study from garbage dumps around Madrid (Blanco 2007, ref. 62, where the extent of rabbit predation does not indicate a high degree of specialization, i.e., 9-23% by number, 16-40% by biomass). The text emphasizes the specialization on rabbits by citing a figure of "up to 80% of the diet", which is the maximum observed in ref. 38 but struck me as an instance of selective citation that implies a considerably higher frequency of rabbits in the diet than was typically observed in refs. 38, 61, and 62 together. Oddly, kites are then described later in the Methods (L587-588) as "aggressive generalists, feeding on more than 250 prey species. Super-generalists of this kind…" These statements are not absolutely incompatible, but they paint very different implications. This is relevant in part to the plausibility of the 'punishment' hypothesis and in part to the repeated assertion that lynx and black kite are actual competitors (which entails not just prey overlap but also a negative effect on population growth rate of one species on the other).

[3d] Supporting inference based on other species. The same study by Sergio et al. 2024 on environmental contamination notes that the red kite population in Doñana "is currently in steep decline for unknown causes". Red kites eat rabbits just as frequently as black kites, if not more so (Veiga & Hiraldo, Holarctic Ecolog 13:67-71). Red kites are much less abundant than black kites, and I don't know their local distribution, but by the logic of this study, lynx should interact similarly with both red and black kites. Is there evidence that they do?

[3e] Absolute quantitative summary/descriptive data. Data in the study are dominated by proportions. There is no problem with proportional data, but would still be helpful and complementary to be informed about the actual absolute numbers and frequencies of events. These can be partially pieced together by stray numbers in the text and on the figure axes; for example, on L633, reference is made to 108 total kite predation events. But one can only piece together a fragmentary picture. Presenting these more systematically to complement proportional data would further add to the transparency and readers' ability to be confident in the interpretation offered. Adopting the suggestion to show data (perhaps in the ED) as time series would be one way to do this, as would stipulating them in the text.

absolute numerical data & temporally explicit data presentation:

[3u] Upshot: By my count, you have 6 available ED figures and one or two main-text figures, and the text, where you can expand on the set of points raised above in various ways
(4) WORD CHOICES
A number of words and phrases are employed that I think should be revised.
— e.g., L. 17 - "peaceful coexistence": Peaceful is a human construct that seems inapplicable (1% of which were killed by lynx during the period of 'peace'). "Neutral" would be better, as is the description on line 104 of how the species "basically ignored each other")
— e.g., L. 20 - "punishment" — see general comment above.
— e.g., L20, L68, L500, and throughout: "Lynx and kites compete for shared prey": what is the evidence for competition, besides shared prey (which is not sufficient basis alone to assume competition). For example, if kites are not eating enough rabbits to have a resulting negative effect on lynx population growth rate, or vice versa, then they are not actually competing event though they share pray. You could say "likely compete" or "potential competitor" or similar.
— e.g., L. 33, L189 - "climate and biodiversity crisis": The word 'crisis' is a subjective characterization (cf. "disaster", "catastrophe", "cataclysm") and dubiously appropriate for a peer-reviewed primary research article about lynx—kite IGP. It is established that the climate is warming, but there is not a scientific consensus, and indeed much debate, as to whether this is appropriately described using terms such as 'crisis' Similarly, it is established that rates of biodiversity loss and population extirpation are accelerating and higher than plausible estimates of the background extinction rate, but here there is even less consensus as to the appropriateness of terms such as 'crisis'. The subjective use of such terms is criticized as politically motivated exaggeration by people on both the far right of the political spectrum (mainly with respect to climate change) and people on the far left (mainly with respect to biodiversity loss, when in tension with human livelihoods). This is one of the factors that is contributing to the erosion of scientists' credibility and the loss of public trust in science.
— e.g., L168 - "worrying": for the same reasons as "crisis". Worrisomeness is not an objectively measurable quantity.
— e.g., L1, and in dozens of other places throughout - "climate change": the word 'change' implies that the droughts in this study are attributable to climate change per se, as opposed to merely being anomalous years; it is certainly plausible, indeed likely, that it is influenced by human GHG emissions, but that not known with certainty. It is appropriate to refer to 'climate' throughout; and it is appropriate to say what models forecst(although note that climate model forecasts vary a lot in confidence and precision, depending on both the specific model and the geography). It is also even appropriate to describe concerns about the impact of climate change on IGP as a motivation for the study. But attributing the drought and ensuing interaction dynamics to climate change, per se, goes too far, notwithstanding the suggestive climatic trends briefly mentioned in the Methods.

(5) PECIFIC MINOR COMMENTS

L1 – Title: Remove the word 'change' or reword with a better alternative (e.g., drought [most precise] / extreme weather event / climate anomaly. Beyond that, I think the word 'disease' must also be in the title. Ultimately, as you show, these are not effects of climate effect per se, but rather an interaction between climate and a rabbit disease, with both being necessary and neither one sufficient to trigger the dynamics.

L25-28 — I suggest a rewrite of these two sentences that summarizes the significance of the contribution without relying on so much jargon. See larger comment above about this.

L102: What are the 1662 nests? Nest*Year combinations? (N = 105-223 kite pairs in the study area)

L115: Are these percentages and cohort stats for a particular year or years? Which ones?

L142: First demonstration of the fitness consequences of IGP on vertebrate predators — are you sure about that?

L145: "climate change" again.

L161: Heightened predator competition — this seems like speculation.

L162: Strong bouts of lethal predation "never observed before" — 1% of kite nests were predated before rabbit declines (earlier in the ms)

L171: "Comprehensive strategies tackling multiple anthropogenic threats". This might sound reasonable at first glance, but on scrutiny, it is a vague and impractical recommendation; it states that all anthropogenic threats must be "tackled", which is unrealistic as a management objective

L186: Avoid using "evolve" to avoid confusion between evolution and behavioral plasticity

L69 and subject heading on L499: Change 'model system' = Drosophila, C. elegans to 'study stem' or swell studied system'. For the subheading on L499 up might change to "Trophic modualg

L502: "that compete.."

Referee #2

(Remarks to the Author)
The manuscript “Climate change precipitates carnivore predation on raptors” investigates the effects of two serial stressors (disease and drought) on a food web in Doñana National Park. It shows that after disease reduces a common prey (rabbits), during drought lynx prey on black kites.

Although this area is definitely less well investigated, I do not agree with the claim that we know virtually nothing about interactions among vertebrate top predators under climate change, and a quick search brings up a variety of papers on apparent competition, predator-prey and population dynamics (e.g., https://link.springer.com/article/10.1007/s00442-017-4017-y). Perhaps this might be better phrased, as “direct interactions” which I do agree with, as intraguild predation is more usually noted as a one-off phenomenon in studies and does warrant more focus.

I also do not find the interpretation of the results, “a punishment arms-race” to be particularly compelling, and without further mechanistic details, feel that there are a wide range of potential explanations (see specific points below). Although this is certainly one potential explanation, I feel that the authors do not offer any alternatives which is slightly misleading.

I found the language was sometimes a little unscientific and distracting (e.g., peaceful coexistence, when simply coexistence would suffice, or punishment of the intraguild competitor). These instances tend to anthropomorphises the interacting species and, in my opinion, reduces the clarity of the narrative. It would be better to use only well-defined and common ecological descriptors of the interactions to keep consistency with the wider literature. Along these lines some of the sentences are very long and winding and could be more succinctly written, for example in the abstract the third sentence is 56 words and 6 lines long, and could be split into 2-3 sentences.

The methods appear robust, however, based on the definitive statements made in the conclusions about “punishment” of competitors, I think there should be more detailed information on the diets of both the predators in order to drive towards some more complete mechanistic understanding. To me, there are quite a few confounding variables in the field study, which means that it is not possible to be confident in the proposed explanation for the observed patterns.

Specific points:

Abstract:
Line 15-20: This sentence is very long. Consider splitting into 2-3 sentences.
Line 18: I would use the Latin binomials to be more specific. Also, you define some taxa but not all. For example you don’t say that the predatory raptor is a black kite, or what the insectivorous bird is on line 23.
Line 24: I would always be very careful about this. It is highly likely that there is already evidence of this to date, and that it is not the focus of the paper. I would rephrase to not say “first” but instead focus on the novelty. I.e., “These results demonstrate a rarely observed climate-induced intraguild predation effect that…”
Line 26: This is a little vague and is not necessarily specific to what you found here – try to specifically link the changes in food webs you observed and make a more specific statement that still speaks to this wider point.

Main text:
Line 32-33: Why are they recognised?
Line 38-39: I don’t agree that this is the case, there is a lot of information around the impacts of climate change on species interactions: see this review from 2008 https://onlinelibrary.wiley.com/doi/full/10.1111/j.1461-0248.2008.01250.x
Line 47-50: Very long sentence
Line 75: Why is the longevity of the lynx also not provided? Why is it not relevant?
Line 75: Is it specialisation if they are abundant? I would rephrase this as it is a little contradictory – if they specialised on rabbits this would not just be when they were abundant.
Line 78: How representative is Doñana of the wider ecosystems? I.e., how generalisable are these results to lynx and kites elsewhere on the Iberian peninsula? Especially considering it is a wetland?
Line 108: This is very non-specific – try not to use phrasing like “ignored each other” it is confusing and misrepresents the structure of ecosystems (e.g., the presence of these taxa will affect behaviour and other aspects, so they do not ignore one another if they are not directly interacting).
Line 122: Use spaces between values and SI units
Line 132: Or is it because these nests are more conspicuous, or that there will be more chicks on that nest (requiring more food) and therefore more energetically efficient. I am not convinced that the evidence you present points towards the fact that this is predation to reduce competition.

Methods:

Line 546: Although you know that lynx predate nests based on the described methodology you do not know what else they are eating, and as a result do not really understand the drivers of these predation events. Without diet data for lynx, it is my opinion that there can be very restricted and quite suppositional interpretation of these data. Faecal meta-barcoding, hard parts analysis or other information on lynx trophic interactions during drought and non-drought periods are needed for the conclusions made at the end of the manuscript.
Line 587: The densities of other prey taxa also need to be monitored to truly understand what is going on in this system, especially if the kites are super generalists.
Line 626: But this higher availability of rabbit prey would also mean higher numbers of rabbits under the nests of the kites though? This is a confound with the findings, as although there are more rabbits for the lynx there are also more likely to be

more rabbits for the kites and therefore have more rabbit remains under the nest.
Line 645: Is there ontogenetic shift in the feeding of kite nestlings as they grow. As to my mind, you can't separate out the fact that larger nestlings will be eating more (and also maybe more rabbits) thus forming a better meal for a lynx, from the fact that the lynx might want to "punish" that competitor.

Figure 2: What is "normal" I would replace this with "non-drought period"
Extended Data Figure 1: The fact that there are far fewer nests also in drought periods is not made clear in the main text. This is an important factor to consider when thinking about interactions. There may be less rabbits, but also less of everything else, and therefore there is an overarching increase

Referee #3

(Remarks to the Author)
This was an excellent and important study showing the dual affects of disease and drought on intraguild predation, with cascading consequences for individual fitness, population dynamics, and species interactions. Using three decades of monitoring data, the authors showed that lynx increased intraguild predation (IGP) on kites during drought periods following a disease-driven population decline in their shared prey, European rabbits. The increase in intraguild predation led to reduced lifetime reproductive success and population decline among kites. In turn, it also led to increased predation by kites on little owls. Such results provide important insight into how anthropogenic impacts cascade through food webs.

This was a well-written and well-designed study for which I find little to critique. In accordance with Nature review practices, I confirm that the statistics are sound and that error bars and CIs are appropriately defined in the figure captions. I am excited for its publication so that I may cite it and share it with my lab members and collaborators.

My only critique is that the mechanism for which the increased IGP during drought is interpreted is that alternative prey sources are scarce during drought, forcing lynx to prey on kites. The corollary during non-drought years was that "lynx likely compensated the rabbit scarcity with abundant alternative prey" (Lines109-110). However, no data or support is provided for this interpretation, yet the explanation for why IGP would increase during droughts hinges on this. Can the authors provide any data or analysis demonstrating drought's effects on alternative prey availability?

Sincerely,
Briana Abrahms

Referee #4

(Remarks to the Author)
Overall assessment:

The study is a compelling illustration of how one stressor can amplify the effects on another, with cascading effects throughout an ecological community. The study thoroughly assesses both demographic and community responses among predators to the combined effects of drought and disease on rabbit populations.

One potential concern is the small spatial extent of the study. Given the 10 km2 study plot (just over 3 x 3 km), is it possible that the effects observed here are quite limited with respect to the entire population of kites, owls, etc.? It would be helpful to know some more information about the distribution of the kite nests within the greater population and if they are particularly concentrated in this study plot for some reason.

A suggestion: An illustrative representation of all observed indirect responses to the drought-disease interaction would be very valuable (rabbit declines, increased lynx predation on kites, decreased kite abundance and reproductive success, increased owls?, decreased alternative prey?) – a sort of graphical depiction of all the results. This would allow the reader to clearly see the main conclusions, and it could also be used in a graphical abstract.

Specific comments:

Line 117 (and elsewhere, e.g. Fig 3): I'm not sure I would call these control nests, as that could imply an experiment. Referring to these as "unpredated" nests seems sufficient and more accurate to me.

Lines 132-137: I agree that one explanation for lynx predating on nests with more rabbit remains is an attempt to reduce competition. A more likely explanation I think would be simply that it is a stronger indicator of an active nests that could be predated. I would be careful not to over-interpret the 'mindset' of the lynx. It would actually be very valuable to know what other prey sources were available during these periods – were the lynx consuming the only reliable prey left, or specifically selecting for their competitors? Any information about abundances of alternative prey should be included.

Line 147: Did kites move to another territory in this same study area or out of the study area entirely? If they left the study area and could no longer be monitored, this could change the inferences about lifetime fitness (perhaps animals that left found better conditions).

Line 156: This makes it sound like higher kite abundance led to lower owls, so that after drought years, owls would have actually increased. It would be interesting to see how owl abundance changed in or right after drought years to fully capture the trophic cascade. Lines 158-160 seem to imply that the trophic cascade decreased owls, so some clarification here would be helpful.

Lines 161-172: While I agree that more work in this area is much needed, this paragraph seems to ignore a fairly rich literature on multiple stressor effects and synergistic effects. Some framing within this literature would better situate this study within what is already known. Some citations provide below.

Fig. 2: I understand the argument that the amount of rabbits in the kite diet is a proxy for availability, but it seems to me that density is a much better one. I would have the x-axis in panel A read “Proportion in kite diet” to more accurately depict the data. I might also change the y-axes to say “Lynx predation rate on kites”, as the reader may not read the legend carefully and the plot alone looks like it would be referring to predation rate on rabbits. Finally, it seems here that there are some drought years with high rabbit abundance. Were those years pre-disease? It could be useful to color the dots by if they were pre- or post- diseases to show the relationship between disease and rabbit decline. One minor stylistic point: I would recommend not using the default ggplot aesthetics.

Fig 3. In general I would prefer more descriptive axes – e.g. in A) percentage of rabbits as kite prey items as y-axis, in-fig legend as predated by lynx vs. not predated by lynx. This would really help the figures to be more easily digestible. Also, what is the difference between “predated” and “suffered intraguild predation” (panels A,B,E vs C,D)?

Extended Data Figure 1, Line 938: Remove “of” after “myriad”.

Multiple stressor papers to consider:

Geary, W. L., D. G. Nimmo, T. S. Doherty, E. G. Ritchie, and A. I. T. Tulloch. 2019. Threat webs: Reframing the co-occurrence and interactions of threats to biodiversity. Journal of Applied Ecology 56:1992–1997.
Hale, S., D. G. Nimmo, R. Cooke, G. Holland, S. James, M. Stevens, N. D. Bondi, R. Woods, M. Castle, K. Campbell, K. Senior, S. Cassidy, R. Duffy, B. Holmes, and J. G. White. 2016. Fire and climatic extremes shape mammal distributions in a fire-prone landscape. Diversity and Distributions 22:1127–1138.
Munns, Jr., Wayne. 2006. Assessing Risks to Wildlife Populations from Multiple Stressors: Overview of the Problem and Research Needs. Ecology and Society 11:23.
Orr, J. A., R. D. Vinebrooke, M. C. Jackson, K. J. Kroeker, R. L. Kordas, C. Mantyka-Pringle, P. J. V. den Brink, F. D. Laender, R. Stoks, M. Holmstrup, C. D. Matthaei, W. A. Monk, M. R. Penk, S. Leuzinger, R. B. Schäfer, and J. J. Piggott. 2020. Towards a unified study of multiple stressors: divisions and common goals across research disciplines. Proceedings of the Royal Society B 287:20200421.
Rojas, I. M., M. K. Jennings, E. Conlisk, A. D. Syphard, J. Mikesell, A. M. Kinoshita, K. West, D. Stow, E. Storey, M. E. D. Guzman, D. Foote, A. Warneke, A. Pairis, S. Ryan, L. E. Flint, A. L. Flint, and R. L. Lewison. 2022. A landscape-scale framework to identify refugia from multiple stressors. Conservation Biology 36:e13834.
Schmitz, O. J., and G. C. Trussell. 2016. Multiple stressors, state-dependence and predation risk—foraging trade-offs: toward a modern concept of trait-mediated indirect effects in communities and ecosystems. Current Opinion in Behavioral Sciences 12:6–11.
Simmons, B. I., P. S. A. Blyth, J. L. Blanchard, T. Clegg, E. Delmas, A. Garnier, C. A. Griffiths, U. Jacob, F. Pennekamp, O. L. Petchey, T. Poisot, T. J. Webb, and A. P. Beckerman. 2021. Refocusing multiple stressor research around the targets and scales of ecological impacts. Nature Ecology & Evolution 5:1478–1489.

Version 1:

Reviewer comments:

Referee #1

(Remarks to the Author)
I appreciate the depth and thoughtfulness of authors' response letter and laughed at the "several weeks of work" required to respond to my original comments. If it makes the authors feel any better, it was at least a solid week for me, too. I'll try to do this one in an hour.

I want to especially highlight my appreciation for the responses (to me and other reviewers) that expressed principled disagreement. There is no need to apologize or be tentative about that (and I will not be tentative about continuing to disagree on a few points, below). Overall, I find the responses largely convincing with respect to the primary concerns articulated by me and the other three reviewers.

Title. I have read and considered the authors' responses to my concern about "climate change" in the title. I still disagree, rather strenuously, and as expressed prevoiusly, I think it is critically important for the scientific community to be disciplined about this. The study is about a chain of events precipitated by an interaction of drought and epidemic disease. Climate change is associated with droughts (along with essentially every other kind of weather phenomenon) but they are not the same thing. I am not suggesting that the authors cannot mention climate change or discuss it in relation to their study. I am not taking a position on whether droughts in this ecosystem are the result of anthropogenic climate change or not. My

position is that the current title of the paper -- "Climate change precipitates carnivore predation on raptors" (a declarative statement of cause and effect) -- misrepresents the contents of the paper and the scientific advance it presents. It is imprecise (climate change is many things, including droughts, deluges, typhoons, record high temperatures, record low temperatures, etc.) and simultaneously narrow (it omits the critical role of disease). The authors' response to my previous comment -- "we really don't see how drought could not increase in the future when the temperature is locally increasing by 0.5/decade" -- acknowledges the assumption being embedded. So I think it is a poor choice even leaving aside concerns about how nonscientists and the media are likely to misinterpret it.

Ultimately I do not understand the authors' rationale for clinging to this formulation. Phrases like "extreme weather" and "drought" will catch just as many eyes without crossing into this gray area:

"Disruptive influence of drought and disease on interactions among top carnivores/predators"

To me, that is an even more compelling title that also more fully and accurately tells the reader what the study about. In that formulation, and many others, climate change is only just below the surface and will be unmistakeable to your primary audience. Reporters will ask you about whether this has anything to do with climate change, and you can say whatever you believe best serves the public interest. And nobody will be able to accuse you of having been slippery with words or misleadingly pushing a "climate change agenda".

The first sentence of the summary paragraph is OK with me (but substitute 'increasingly' for 'growingly'). But for all of the same reasons, I would change at least the 2nd mention of "climate change" in the summary paragraph to "extreme weather events" or "climatic perturbations" or similar.

23. "In the future, synergistic effects of multiple..."

24. "keystone species" -- this to me implies that all top predators are keystone species, which is simply not true. I don't see the need for this term, which opens up a whole box of worms. The word keystone appears three times in the summary paragraph alone. See comments above about climate change. I continue to sense that the authors are trying to 'sell' the study by using terms that they consider flashy, and I continue to advise them to temper that instinct. The study is plenty flashy enough; it doesn't need any extra dressing.

See also "virtually nothing" (line 37; --> "little"), "urgent" (line 51; --> "There is a need"), "iconic" (line 54 --> "One common interaction among predators"), etc. If the authors could just tone it down a bit, throughout, the whole thing will come across more convincingly. We all make up our own minds whether a study is interesting and important or not; insisting too breathlessly on these things only tends to make us suspicious.

This advice extends the "competitor reduction" interpretation -- the authors have done an adequate job of flagging this issue (a concern identified by 3 of the 4 original reviewers), but I think they could still push it even less, with stronger net effect. If it were my paper in review at Nature, I would introduce it like this:

"One interpretation of these results is that lynx are systematically removing competitors during potential starvation years. We find this interpretation plausible -- and in fact probable -- for the following reasons 1, 2, 3 ... An alternative hypothesis is A, but that wouldn't explain B or C. Another possibility is X, but X doesn't explain Y or Z. [i.e., acknowledge and proceed to convincingly rebut the lines of argument brought by Reviewers 1, 2, 4]. Ultimately, we find the first interpretation to be the most plausible, although additional manipulative experiments will be required to confirm it." I advise doing it that way.

When I read something like that -- OR LIKE THE WAY YOU EXPLAINED IT TO ME IN THE RESPONSE LETTER -- where the force is carried in the impeccable logic rather than the 'hard sell', I think "These authors are not trying to beat me with their sexy interpretation, they are presenting the evidence and counterarguments, and the evidence and arguments makes sense. I respect the calmly confident and incisive way in which they weigh evidence, giving no impression of having a 'pet hypothesis' but simply judging propositions in light of the data. I believe them."

Conversely, the hard sell always puts me in fight mode: "I feel my arm being twisted, and I don't like that. I'm going to search for problems in the story." And you can see evidence of this in my previous review. I went and tracked down all those old references and papers about Doñana because I felt you were trying to sell me a half-baked cake; I don't like paying full fee for a half-baked cake, and I don't know anybody who does (and anyone who does buy such a cake soon learns that it was not well enough baked)

My recommendation on this next revision: Take a big step back, let the manuscript sit for a few days, and go through it and simply try to find all the places where somebody like me might argue that you are overselling -- and cut and trim and deflate any such instances you find. I know that I'll be citing your study, but I'll feel better if you follow not just the letter but the spirit of what I'm trying to communicate about this.

37-39 - This sentence, which at least two reviewers flagged for concern because of "virtually nothing" (and for me, invoking "climate change" as the only relevant driver -- you seem to want to hide the disease element?) -- anyway, the sentence doesn't seem necessary. You could cut it, the segue would be smooth, the focus still general, and you would dive down into IGP starting on Line 54, and you would not have to repeat so many iterations of saying that this or that is unknown: "We know virutally nothing" (12, 37), "precious little knowledge" (60), "virtually no research" (61), "An even less known" (68), "completely uknown" (75)

40-54 I like the new second paragraph...

76-78 ... But I don't see the need for this paragraph. This has all been said. There is no need for a synopsis at this stage of the ms -- and no need to say yet again that "we lack key knowledge", a point made at least 6 times already according to my list above.

79-116 I would break up this extremely long paragraph

Figure 3 is another very helpful addition. I would like it even better if you could show a shaded AUC with rainfall (not just drought) across the years. You don't have a spare vertical axis for this, but one is not really needed -- the relative trends in rainfall (peaks, valleys) will tell us what we need to know about rainfall even without precise numbers

Extended Data 5 and 6 are both nice. You say in the response letter that you want to keep the former, so by all means keep the former.

On the whole, I think that the authors have done their due dilligence in addressing the most salient concerns, and I am convinced by the core of the story. My two main lingering concerns are laid out in great detail above and mainly focus on presentation. The 'competitor retailiation" argument is -- I think we all agree -- not ironclad but at least as consistent with the available evidence as anything the reviewers have raised. The authors can still do more to build the case gently, and therefore more convincingly, but at this point the difference is more stylistic than substantive (although presentation is important!) One can always dream up more data that would be nice to have, but those do not seem strictly essential to support the core conclusions of the work.

I hope that this second review, while puny in comparison with my first, will nonetheless be helpful in further improving your impressive study. I don't think this one will require quite so many weeks of work.

[redacted]

Referee #2

(Remarks to the Author)
Reviewing the revised manuscript, it is clear the authors have done a fantastic job in revising the manuscript in response to 4 challenging reviews. Many of the points I raised were satisfactorily answered, and I think the reframing of the manuscript that has been completed in response to my (Reviewer 2) and Reviewer 1's comments really improves the work and means that it feels much more rounded as opposed to using the data to tell a specific story. Saying this, I still do not feel that the conclusions can be robustly supported without dietary data (and other contextual data that has not been collected), and for this reason I do not believe that it meets the high bar that is set for publication in Nature. Without these data, it is still not clear what the mechanisms are for these changes and thus the generalisability to other ecosystems is not possible. Although this, in my opinion, precludes the publication in a journal such as Nature, it would make a fantastic contribution to somewhere like Ecology Letters, Global Change Biology or other journals with a strictly ecological focus, where the reader can assess the generalisability themselves and make more informed interpretations. Although I appreciate this might be disappointing for the authors considering the efforts that went into revising the manuscript, this work will not be wasted and I look forward to seeing the work published in the near future.

Referee #3

(Remarks to the Author)
The authors have fully addressed my main concern. I appreciate them adding data on duck abundance in the revised Figure 3. This now much better completes the picture and supports the authors' interpretation.

Referee #4

(Remarks to the Author)
I appreciate the authors' careful consideration of my comments, as well as those of the other reviewers. I am largely satisfied by the revisions. While I remain somewhat skeptical of the competitor removal argument, the authors have made a strong case and have much experience on the ground observing the animals, so I find their explanation sufficient. Figure 3a is a very compelling illustration of the multiple stressor effect. Overall, I think this paper is a great contribution in showing how multiple stressors can rewire ecological interactions.

Referee #5

(Remarks to the Author)
This study is a very interesting example on detailed ecological interaction between species and how multi-disciplinary approaches/data can provide additional understanding of observed behavior. I found the reading interesting and authors' arguments about the importance are convincing. However, there are still some improvements and clarifications necessary to make this study sound for Nature publication. Here are my main critics:
1) Terminology and expressions are often less scientific and express often subjective "feelings" rather than facts based on data and analyses. I do totally understand that some statements are based on long field experience, which are very

important. However, I would suggest to underline those observations/statements more objectively (please compare detailed comments).
2) The presentation of spatial data (Extended Data Figure 1 and 2) should be improved. That will make more professional impression and provide additional transparency about used data.
3) Additional Extended Data Figures showing the distribution of number of monitored kite nests over time would also be of advantage as well as a Figure on available inundation maps and interpolation.
4) Additional Extended Data Figure presenting meteorological and hydrological parameters (e.g. using data from meteorological and hydrological stations from the wetlands measurements network)
5) In regards to inundation data/information, there are definitely some issues which has to be followed up to avoid criticism and jeopardize the interpretation and outcome of this study.
General comments on surface water maps, their assessment and importance:
I totally understand the focus and narrative of this study. The used information about flooding is simply an additional input to quantify whether a year could be called as a drought year or not. Therefore, the focus should not be on the flood mapping approach. Therefore, used inundation maps and provided explanation shall raise as less questions as possible. However, because "drought" seems to be a major factor in the narrative and explanation of observed increase of kite predatory by lynx due to absence of surface water and ducks, I would recommend to provide more transparency. Furthermore, I would like to provide some suggestions and recommendation to the authors, which could be taken into account to improve that part and make it less vulnerable to critic. Since this manuscript is under evaluation for a Nature publication, I would expect that all aspects are at highest scientific and presentation level. First of all, the water mapping methodology which is presented in https://doi.org/10.3390/rs8090775 is very enough and can be considered as accurate surface water classification for the study area. Especially, because studies focusing on a local environment produce mostly more accurate results in comparison to approaches mapping surface water on larger scales (nation, continental, global). This could be a good argument why the authors are using "in house" inundation maps. However, the approach was published ten years ago in 2016. Since then, there was a big progress in satellite remote sensing data, using cloud computing and AI to fully exploit multi-sensor data and new additional open-source datasets (e.g. from Copernicus program Sentinel-2 would be the most relevant dataset). In this way, spatial resolution as well as temporal frequency can be increased tremendously. I believe that both aspects are important for presented study. Therefore, I see a lot of potential critics in that direction as this progress is fully neglected. To avoid this, I recommend that the authors transparently present more details on inundation maps and discuss existing draw backs and why those are probably irrelevant for this study analyses. I highly recommend to involve an experienced remote sensing data scientist and GIS expert to spend time and help improving that part and additional Figures and this way avoid unnecessary and avoidable critics in that direction which could compromise your major interpretation. In the study from 2016 https://doi.org/10.3390/rs8090775 the authors of inundation maps already mentioned: "The method is being updated for Landsat 8 OLI and Sentinel-2 images in order to extend the time series of flooding masks and hydroperiods." If that was the case, that should be presented here as well or at least discussed why you do not use additional data from Landsat 8 and Sentinel-2.
Detailed comments with reference to lines (L) in revised manuscript. Original text or terms which I refer to are placed in „…text…"
L 12: „we know virtually nothing" and L 37-39: "For example, we know virtually nothing about how climate change affects direct interactions among vertebrate top predators, such as intraguild predation"
This is a crucial statement, which I would have expected to be referenced or underlined with a review. Otherwise, I hope you are aware of the impact such statement can have and can hold your argumentation.
L 95 "…over coming years"
expression is quite vague. Please use exact future temporal interval you are referring to from cited references. Usually, climate change scenarios provide specific intervals.
L 116: It would be interesting to see the distribution of 1662 kite nest over the three decades as a Figure (at least in Extended Data). That would help to get a better feeling on the background data. For example, how many nests were monitored and how many destroyed kite nests were observed for each year? In L 775-776 you do provide averages, but a full transparency in data presentation is necessary.
L 118: The numbering and mentioning of Figures are not correct. The very first time a Figure is mentioned is Figure 2 in L 118. First time Figure 1 is mentioned is L 683. Please correct accordingly.
L123-124: "During normal non-drought years…"
The term "normal" is somehow subjective. Shouldn't it be based on long term climate measurements? I can imagine that climate scientist might ask for long term measurements and statistic on which you consider a year as "normal". E.g. certain annual precipitation +/- StdDev? Certain distribution of monthly precipitation over the year?
L 239: "…mix of good and bad news" sounds very unscientific to me.
L 592-593: "…which inundation levels…"
The term "level" refers to height of surface water. Please use inundation area instead. Also in L 607.
L 601: "Global accuracy…"
this term is quite unusual and misleading. Usually, in remote sensing the term "overall accuracy" (taken into account over and underestimation) is more common to express what you probably mean by "global accuracy"
L 602-603: GitHub link does not work. I could not see the protocol, nor the code or flood masks.
L 606-608: "We then extracted the minimum inundation level reached on these fixed dates and classified a year as drought when such level was lower than 20 % of the maximum inundation."
Here, I would like to raise two issues. First, could you please clarify if 20% of maximum inundation are referring to maximum inundation of the same hydrological year or to long-term maximum inundation observed over the entire study period?
Secondly, I would recommend that the authors critically discuss whether they would really like to use the term "drought". If that is the case, more information and more background on drought would be necessary because the term "drought" in scientific sense is complex and defined differently depending on the discipline (e.g. hydrological drought, meteorological drought, agricultural drought). Since the kite predatory seems to depend on absence of surface water causing a reduction of

ducks, I personally would feel more comfortable to use the term “surface water availability” instead of drought. Especially, as I assume that the absence of surface water in that wetland is not ONLY affected by changing precipitation regime but is a combination with water management and overuse for agriculture. I believe to cover all that aspects will go beyond the objectives of this study. Although, I believe that this study would highly benefit from a transparent presentation of meteorological and hydrological data. For example, one Figure showing monthly temperature, total precipitation as well as river discharge or river level. That would present climatological variability of study region which is an important background information to understand the climate variability context.
L 627: “15 May” is that correct? Before you used 15 March. If 15 May is correct, I wonder why you use this date to estimate spring precipitation? Its neither meteorological nor astronomical spring period. Could you please explain?
L 629-630: “So defined, anomalous years coincided with the drought years listed above, but with the addition of 2011, given its copious spring rains”
It is unclear to me why you add 2011 if there was more rain than usual? Or is it a translation mistake?
L 776: “(N = 64.9 checked nests/year on average, range = 20-103 and 52-86 after 2013)” –
> missing the range before 2013
L 777: “Data on rabbit density were only available since 2005. For the preceding years, rabbit density was back-estimated by a regression…”
This HAS to be also indicated in Figure 3!
L 949, L 1227, L 552: should be MSS (Multispectral Scanner System) not only “MS”.
L 599-600: Landsat MSS has a spatial resolution of 60m!
L 604-606: “To make inundation rates comparable across years, we linearly interpolated consecutive inundation masks so as to estimate the inundated area for the fixed dates of 15 February, 1 March, 15 March and 30 March every year.”
This is a crucial moment which you need to prepare as transparent as possible. As you mentioned, usability of Landsat is depending on cloud coverage. Landsat frequency is 16 days for used data. The results of linear interpolation will highly depend on “when” and how many scenes were available. According to Figure 5 from https://doi.org/10.3390/rs8090775 the number of flood masks can be less than 10/year. A figure spanning the three decades showing the time series of derived inundation area and interpolation curve would be very helpful to see that the used flooding data does not introduce some bias because of scarcity of Landsat scenes during critical season. Additionally, a plot of river level or discharge which hopefully will show similar temporal variability with your interpolation would strengthen your argumentation and show that the approach does not neglect surface water variability.

L 1063: Figure 3: Why is there a drop of rabbits in early nighties?
Ext. Fig. 1 and Ext. Fig. 2: Basic cartographic information is missing: scale bar, coordinates, what is used as background image, which month or year are the flood masks from?
Ext. Fig. 2: Would it be possible to present all nests within the study area? This example still can be remained as a detailed zoom-in view. If the number of locations is too large and too dense, some aggregation method could improve the visibility. Why is that important? I assume, the locations of nests, their spatial distribution and density of affected and none-affected nests would be of high interest for the reader.

Additional information for surface water, e.g. to compare and get some additional insight on surface water in your study area from Landsat archive (1984-2021), have a look here:

https://global-surface-water.appspot.com/map
https://www.nature.com/articles/nature20584
To have a daily surface water time series with coarser resolution (250 m, 2003-2024) you can have a look here:
https://www.nature.com/articles/s41597-024-03328-7

Version 2:

Reviewer comments:

Referee #5

(Remarks to the Author)
Dear Dr Sergio and co-authors,

Thank you for your detailed replies and revisions. Here are my comments for this round of review.

In general, that version is much improved! My main concerns about terminology and with few statements “overselling” were taken care off. Especially thanks to reviewer#1’s comments and very well explained concerns and authors revisions in this version.

Comment 8. REVIEWER 5 from last round:
Thank you for detailed explanation and arguments. I see your point and I can understand where you coming from with your explanations. I personally am okay with that. I also agree that spatial resolution of 10 m vs 30 m would not introduce much

difference in that case. However, temporal resolution is much more important for your case study and how many cloud free images you have over your study period. Anyways, I cannot judge whether the inclusion of denser time series would in fact have increase the number of cloud-free images and thus the number of water mask contributing to more stable and robust results. At the end, it is your decision and judgement based on your field experience and observation of flood dynamics and what additional mapping efforts are practicable and which additional outcome of water mask time series might contribute for any kind of improvement. My goal and responsibility as a reviewer were to underline the existing opportunities and I also did that because it was mentioned in the water mapping study from 2016 that the “in-house” methods would be modified for Landsat-8/Sentinel-2.

Best wished and good luck with upcoming rounds,
Igor Klein

Open Access This Peer Review File is licensed under a Creative Commons Attribution 4.0 International License, which permits use, sharing, adaptation, distribution and reproduction in any medium or format, as long as you give appropriate credit to the original author(s) and the source, provide a link to the Creative Commons license, and indicate if changes were made.
In cases where reviewers are anonymous, credit should be given to 'Anonymous Referee' and the source.
The images or other third party material in this Peer Review File are included in the article’s Creative Commons license, unless indicated otherwise in a credit line to the material. If material is not included in the article’s Creative Commons license and your intended use is not permitted by statutory regulation or exceeds the permitted use, you will need to obtain permission directly from the copyright holder.
To view a copy of this license, visit https://creativecommons.org/licenses/by/4.0/

Dear Dr. Armstrong,

Thank you very much for the attentive comments by yourself and the Reviewers on our manuscript titled "Climate change precipitates carnivore predation on raptors". We are glad that the Editor and Reviewers made several appreciative and constructive comments about our work and suggested ways in which we can improve it. We have done our best to implement all the suggested changes and we believe that the manuscript has gained much clarity and solidity from following the Reviewers' suggestions.

To make things easier for the Editor and Reviewers, we have written *in italic* the comment of the Reviewers preceded by "REVIEWER" and in standard-text, our response preceded by "AUTHORS". Please note that:

- we have shortened a few comments by the Reviewers where these simply summarized the study;
- for some comments by the Reviewers and our answers, we have underlined some portions of the text in order to draw attention to relevant aspects of it.

COMMENTS BY THE EDITOR (Dr. Anna Armstrong):

1. **EDITOR**: *Your manuscript, "Climate change precipitates carnivore predation on raptors", has now been seen by four referees, whose comments are attached below. While they find your work of potential interest, as do we, they have raised important concerns that in our view need to be addressed before we can consider publication in Nature.*

Should further work allow you to address these criticisms, and provide compelling evidence for the combined effects of disease and drought on intraguild predation, we would be happy to consider a revised manuscript (unless something similar has been accepted at Nature or appeared elsewhere in the meantime).

AUTHORS: We have attentively studied all the Reviewer's comments and we believe we have added all the requested analyses, figures, conceptual framework and methodological details. We have accelerated the revision as much as we could, given the Editor's last sentence in parenthesis.

2. **EDITOR**: *As part of the revision we will need you to provide full details of how you derived inundation from the Landsat data. We will also need you to provide the code. And we will need you to make all the inundation maps available through the Data Availability Statement. If the paper goes back to review, we will add a new referee to assess how the inundation maps were made.*

AUTHORS: The code and data are already public (see below). We were already giving for granted that we would place all the inundation shapefiles in a public repository if the paper is published. Please note that we did not derive the inundation masks from the Landsat data by ourselves. We used the inundation masks prepared as GIS-shapefiles by remote sensing professionals of the GIS and remote sensing unit of our institute. The method has been already detailed, extensively ground-validated, refereed, published on the journal "Remote Sensing" and used many times. See for example:

- Díaz-Delgado, R., Aragonés, D., Afán, I., & Bustamante, J. (2016). Long-term monitoring of the flooding regime and hydroperiod of Doñana marshes with Landsat time series (1974-2014). Remote Sensing 8. https://doi.org/10.3390/rs8090775.

- Bustamante, J., Aragonés, D., & Afán, I. (2016). Effect of protection level in the hydroperiod of water bodies on Doñana's aeolian sands. Remote Sensing 8. https://doi.org/10.3390/rs8100867.

In re-reading the manuscript, we realized that, effectively, we had not given enough details about the inundation data. In the current version, we have added more detail and referred to the papers that established its methodology (lines 593-603). Specifically, about the Landsat data, we have added the following text: "Flooding was assessed by accessing inundation masks for the Guadalquivir marshes provided by the "Remote Sensing and Geographical Information System Laboratory" (LAST-EBD) of Doñana Biological Station-CSIC and routinely employed in scientific assessments of the ecological consequences of marsh flooding (e.g. Díaz-Delgado et al. 2016, Bustamante et al. 2016). Inundation masks were generated by accessing cloud-free Landsat MSS, TM and ETM+ scenes for the Doñana region. Images were radiometrically normalized to a reference image and transformed into reflectance values to produce final inundation masks based on pixels of 30 x 30 m, which were classified as inundated for values of normalized reflectance < 0.186 (Kappa = 0.66, global accuracy = 94%; Díaz-Delgado et al. 2016). Details of the method and its extensive ground-truthing can be found in Díaz-Delgado et al. 2016 (see also protocol and code in: https://github.com/LAST-EBD/Protocolo_v4*).*"

COMMENTS BY REVIEWER 1:

We appreciate the time and energy generously dedicated by the Reviewer to improve our manuscript. We perceived interest and intent to improve the manuscript, which is not given. We believe the manuscript has been improved by several of the points raised. We have disagreed on a few points and on some interpretations of past studies conducted in Doñana. We hope that our comments are expressed with the kindness that we intended to convey. Written text can be misleading in this kind of context, and it would not be our intention to appear defiant or confrontational.

1. **REVIEWER 1**: *This study is based on detailed monitoring of black kite nests over 30 years in southwestern Spain (n = 1662 total nests with individual level marking and monitoring). This <u>exceptionally long-term research program</u> has enabled the authors to document a series of major ecological changes over time.*
The study describes shifts in ecological interactions arising from an interaction between two major human perturbations… [the Reviewer provided a comprehensive summary of our study].
## Strengths:
Such long-term studies of wild populations are <u>extremely rare and inordinately valuable</u> – dynamics such as those documented here can only be captured via long-term study, making this a <u>great example of why it is important to sustain and fund long-term ecological research</u>. The chain of events described is <u>totally captivating</u>, and it is also instructive about the kinds of dynamics that accompany major environmental changes caused by human activity. The story is compelling for a general audience. Non-ecologists and non-scientists are fascinated by complex chains of species interactions and unexpected animal behaviors, as evidenced by the fact such papers are often covered in popular media.
The methods reported are sound. I am convinced by the fieldwork methods as described in the ms for (a) the long-term kite census of individually banded birds; (b) the authors' approach to inferring lynx-on-kite predation events; and (c) the distance sampling approach used to quantify rabbit densities. <u>The value of the intensive long-term study of the kite population</u> (and in PND, an unusually well studied

system) cannot be stressed enough. I am persuaded by the general approach to statistical modeling, which entailed fitting generalized linear models and mixed-effects models; that is the same general approach I would have used.

AUTHORS: We are extremely grateful for the kind words by the Reviewer and feel very honoured that she/he appreciated the study. We spend 5-6 months/year living within the park, renouncing to our families for long periods and under physically taxing fieldwork conditions. Obtaining continuous financial support for decades and finding students ready to accept the physical difficulties is becoming progressively more difficult, in line with the well-demonstrated global declines in fieldwork studies. Because of the sacrifices involved, the appreciation of the Reviewer for this kind of studies means a lot to us.

2. **REVIEWER 1**: *## Weaknesses*

One weakness of the current submission is that while the natural history is unambiguously cool, the explanation of its scientific significance is unsatisfying. In in the Summary Statement (abstract), the authors use ecology jargon in ways that will (a) be confusing to non-ecologists and (b) unintentionally makes the study seem narrower and less significant than it might be. Moreover, the concluding paragraph, leading with: "Forecasting the biodiversity effect of climate change will be challenging given the complex, interactive, context-dependent, unexpected and novel dynamics observed in even in our small trophic module" is downright depressing. It implies that no major advance has been made in this study, as we are already all-too-well aware that ecology is complicated and that forecasting ecological dynamics is tricky. However, I think the authors are overlooking a theme that unifies their results with a larger literature, which might help them contextualize the results in a more convincing and productive way.

AUTHORS: We understand and agree with the concerns expressed by the Reviewer. We have followed all the above suggestions, which are expressed in more detail below, together with our more specific answers.

3. **REVIEWER 1**: *A bigger issue is that the manuscript does not (yet) clear my bar for rigor of inference and interpretation. While I find no problem with the data or the general analytical strategy, I have multiple concerns about the authors' decisions about which data to show, how to show those data, and how much can be inferred or insinuated based on the data presented. These concerns vary in seriousness. Some are minor (e.g., the authors use a number of terms and phrases in the text that I think are poorly chosen or not empirically justified); some are medium (e.g., the authors appear to selectively cite evidence from prior work in ways that are maximally convenient for their interpretation); and some are potentially serious but impossible for me to evaluate (e.g., the analyses presented, while none of them 'wrong', do not directly or maximally test the inferred chain of events). My biggest concern is that there are a lot of relevant data that are not presented and potential corroborating analyses that appear not to have been conducted, and some of the decisions about how to present the data are counterintuitive, which leaves many questions unanswered. There is plenty of room in the manuscript and extended data for more information and analysis, so concerns about brevity and word/figure limits are not a valid excuse for omitting these. Not all of my concerns will necessarily require additions to the manuscript; in some cases, they may only require clarification (but in such cases, I would like to see supporting information or justification at least in the response letter).*

Altogether, this set of factors leaves me with the uneasy feeling that there may be more to this story than the current presentation reveals – or perhaps some inconvenient issues that the authors wish to avoid having to explain. This hunch may be wrong; and even if it isn't wrong, the authors may be able to clarify all issues in the revision and responses. I organize my comments by number below, as some are long.

AUTHORS: We understand the point of view of the Reviewer (see specific point-by-point comments and answers below). We are sorry that the Reviewer felt as if we were possibly trying to avoid some "inconvenient issues". We agree that some aspects could have been

developed in more convincing ways and we hope that we have satisfied all the issues raised (see below).

4. **REVIEWER 1**: *Motivation and significance statements Question for the authors: What is the potentially generalizable advance in our understanding of how interacting anthropogenic stressors influence ecological communities?*

The ms articulates one very good general motivation for this study, namely: The most critical effects of climate change will not be predictable based solely on the weather and species' physiological tolerance. Instead, effects will be mediated by species interactions, which makes them harder to predict (L32-47). From that idea, however, the authors proceed directly into IGP (L42), and thence to successively narrower motivations: the limited understanding of IGP, and the small number of IGP studies that involve "killings among taxonomically distant predators" (unclear why this matters). The synopsis of 'study implications' on L24-26 states: "These results provide the first demonstration of a climate-induced intraguild predation impact that amplifies multiple global change drivers in vertebrate communities." This is the jargon noted above that, ironically, makes the study seem narrow despite the fancy-sounding words (one theory: the profundity of any study is inversely proportional to the number of priority claims and puffed-up words.)

But I suspect this was merely a miscalculation by the authors, and that more can be done to link this study tangibly to a broader literature about behavioral / trophic plasticity and its implications. Specifically: Drought is one common source of food limitation, but there are others, including other types of extreme weather that projected to become more frequent and/or intense with climate change (heat, cold, flood, storm) along with other kinds of human impact (overhunting, environmental pollution, land-use effects, introduction of nonnative species and pathogens). A more inclusive framework might be the tendency of stressors (and their interactions) to induce extreme dietary plasticity and strong direct and indirect effects. Example A: Major tropical cyclone causes food limitation, spatial redistribution, and shifts in ungulate and carnivore communities (Walker et al. 2023 Nature; ttps://doi.org/10.1038/s41586-023-06722-0).

Example B: Depletion of native prey intensifies predation on livestock by carnivores, which in turn leads to retaliation killings by pastoralists (Woodroffe et al., 2000-2005: e.g. https://doi.org/10.1111/j.1469-1795.2000.tb00241.x). In these and other examples, a primary impact of human activities is depletion of prey abundance, which has alternative potential outcomes: The predator population might (a) collapse or fall into conflict with humans (as many observers have feared / assumed), or (b) exhibit resilience via previously undocumented behavioral plasticity and food-web rewiring (brightening the prognosis?).

Recent work using new technology and/or clever fieldwork suggests that the latter scenario is common, whether in response to largely natural or highly anthropogenic perturbations. Example C: Collapse of Sitka deer population in Alaska caused wolf (putative deer specialists) to switch to sea otter, a totally unexpected prey (Roeffler et al. PNAS: https://doi.org/10.1073/pnas.2209037120).

Example D: Pumas in Patagonia (putative deer specialists) become penguin specialists to subsist in a new national park (Serota et al. 2025 PRSB: https://doi.org/10.1098/rspb.2025.2172). Example E: Jaguars in Costa Rica (putative large-mammal specialists) are heavily subsidized by sea turtles (https://doi.org/10.4013/nbc.2017.121.01).

These phenomena connect to fundamental problems in ecology and conservation: (a) forecasting impacts on food-web structure, community stability, and species-of-concern; (b) distinguishing between realized vs. fundamental niche breadth; (c) whether such plasticity is a sufficient to buffer against human activity; (d) frequency and extent of food-web rewiring. The authors vaguely allude to this idea on line 177-179 but then revert back to IGP-specific considerations, including the argument that "IGP will require much more consideration in climate change research and assessments" – an unlikely prospect. The focus on lynx is also fortuitous in this regard, because it can be set up against the famous Canadian lynx-hare dynamics, which suggests an outcome different from that in this study.

This framework can be incorporated more effectively by adding a new paragraph in between the first and third of the current Main Text, as a bridge between the key idea that "biotic interactions modulate climate effects" and the more specific issue that "IGP is poorly understood". Both of those

are still relevant, but IGP then becomes a specific manifestation of the more general problem of (a) shifts in niche breadth in response to anthropogenic stressors and (b) the population / community consequences of those shifts. Later in the article (and in the Summary Paragraph), this framework can also be used to more effortlessly and convincingly explain the how this unique long-term study enriches our growing understanding of an emerging shift in our understanding of what resilience can look like and what ecological byproducts it produces. The same framework also suggests targets for future work (points a-d in the prior paragraph), which are not clearly articulated in the current submission, but should be. A paragraph along these lines would be a fitting replacement for the rather generic, discouraging, and in my opinion outdated views offered in the current concluding paragraph. It would also fit well with a shift towards more scientific neutral language about the biodiversity and climate "crises," which I recommend below.

AUTHORS: We thank the Reviewer for proposing ways in which we can increase the generality (and optimism) of our findings. We have looked in detail at the examples provided. Example A *(*Walker et al. 2023 Nature; ttps://doi.org/10.1038/s41586-023-06722-0) seemed particularly aligned with the Reviewer's requests and we have done our best to follow the conceptual approach of such example. In particular:

a. we have now fully incorporated the framework, concepts and examples suggested by the Reviewer;
b. we have added the requested third paragraph (lines 40-54). Its last sentence tries to connect it to what follows about intraguild predation, in order to maintain a coherent flow of arguments.
c. We reported the implications of the results according to this newly-incorporated framework in lines 206-219 of the discussion and in lines 22-23 and 25-27 of the summary, as requested by the Reviewer.
d. We completely rewrote and restructured the concluding paragraph of the discussion (lines 237-255), which now also includes the "targets for future work" and management implications, as requested.
e. In so doing, we believe we have deleted all the sentences with "jargon" not liked by the Reviewer.

Two important points:

- First, to be sincere, we did not see our original text as so "bleak" and discouraging, it was not our intention. We had proposed a focus on ecological role (keystone species that act as "biotic multipliers") as a tool to forecast future impacts of global change. This was based on previous, (in our view) constructive propositions (Zarnetske et al. 2012. Biotic multipliers of climate change. Science; Urban et al. 2017. Searching for biotic multipliers of climate change. Integrative and Comparative Biology) and seemed particularly adequate in the context of this study on keystone predators and given its results. The Reviewer proposed a focus on phenotypic traits and plasticity. Both seem important to us and one does not exclude the other, so we have tried to integrate them both in the current version, but given more emphasis to the one proposed by the Reviewer.
- Second, we shortened and simplified but retained some sentences on the unpredictability of our system that the Reviewer saw as discouraging. We suddenly witnessed interactions that were virtually absent or anecdotal for some 30 years and we believe that this is a striking, important aspect of the study. We see it as very ecologically

interesting rather than discouraging and we believe that the incorporation of the Reviewer's proposed framework now also highlights its positive aspects.

- The current text now integrates our previous views with the new framework-concepts proposed by the Reviewer (some 70-80 % of the new text). We hope that this new version has satisfied the Reviewer's needs and we express our gratitude for pushing us to improve the generality and optimism of our findings.

5. **REVIEWER 1**: *THE 'PUNISHMENT' INTERPRETATION*

Plausibility of this interpretation

The authors strain to interpret the 'rabbit-remains under nests' result as evidence that lynx are 'punishing' kites for eating rabbits. This is stated in the Summary Paragraph (L20), Main Text (L132), and Methods (L647). The wording used ("consistent with") subtly telegraphs that the authors are aware that this interpretation is a stretch; conspicuously, this particular interpretation is also left out of the conclusions, probably for the same reason. Yet, the way it is presented elsewhere, without any mention of plausible alternative interpretations, will give casual readers the impression that it has been conclusively shown.

As far as I know, the authors' interpretation is technically possible -- it certainly makes ecological sense to kill off a competitor during lean years, and one could fabricate evolutionary just-so stories about how lynx might have gained this tendency. But unlike when apex carnivores kill mesocarnivores that scavenge their kills (interference competition, a direct interaction), this idea requires lynx to cognitively process that the presence of rabbit remains under a nest == a competitor (exploitation competition, an indirect interaction and a more abstract concept). I don't know if lynx are capable of that; but I do know that even university students often fail grasp the distinction between interference and exploitation competition. Stranger things are true, but the burden of proof is to show it experimentally; one cannot assume it.

The plausible (probable) alternative hypothesis that are that rabbit bits / scents are simply strong cues that put lynx into hunting mode – either because rabbit smells like food and lynx want to find it, or because lynx have learned to associate rabbit on the ground with food in the tree. That would be a sufficient explanation and does not entail any association between kites and competition. I see no basis in the paper for excluding this hypothesis in favor of the one offered in the ms, and without knowing more about lynx brains, I would default to the simpler interpretation.

I think that the authors are justified in briefly introducing their idea in the latter portion of the main text, but they should very clearly label it as a hypothesis to avoid any possibility of readers misinterpreting it, and they should pair it with some alternative hypothesis (such as I suggest above). Expressing it as a 'result' in the Summary Paragraph and text (L136: "novel insight on the mechanisms and motivations driving IGP") is not defensible, even with the authors' subtle verbal qualification ("consistent with")…

AUTHORS: We appreciate the Reviewer's criticism. Similar issues with the "punishment" interpretation have also been raised by Reviewer 2 and 4. We try to clarify all the raised issues and how we dealt with them in the following bullet point list:

a. We used careful wording like "consistent" and "suggested the idea of…" because we are convinced that the proposed explanation of competitor removal is the most likely one (see reasons below), but we are well aware that proper experimentation would be needed to prove the competitor removal hypothesis in a fully convincing manner, hece the cautious original wording. For the future, we are planning *ad hoc* experiments (placing experimental rabbits under kite and control nests during drought). However, this will require proper bioethical approval and proper permits by the Park Authority, which may or may not be issued.
b. the "punishment" wording was clearly unfortunate. We wrote it from the "point of view" of the kite victim. Kites that consumed rabbits during drought received a penalty (higher probability of lynx predation) i.e. a "punishment" in terms of offspring production. We agree that this term causes confusion (e.g. in terms of lynx cognition),

so we have substituted it with "competitor removal" (see reasoning below) and have conveyed more caution and the need for proper experimentation both in the summary and when reporting the interpretation of these results (lines 148-159).

c. While waiting for proper experiments, with the currently correlative evidence we are left with the intriguing pattern of higher occurrence of rabbits under (but not inside) the kite nests predated by lynx. The Reviewer criticizes the idea of competitor removal as an explanation on the basis of various arguments, listed below together with our doubts about them:

    c1. first argument: while interactions over carrion allow direct identification of a competitor (there is food and there is a competitor trying to access it), it would be cognitively challenging for lynx to identify a competitor by the fact that they see rabbit remains under the nest. We find this argument a bit puzzling. In our case, lynx sees consumed remains of its favoured prey under an active nest (there is food and there is a competitor that has already accessed it, i.e. the kite parents and nestlings). The direct link with exploitation competition seems as easy or stronger than the interaction over carrion, which the Reviewer finds acceptable. In our view, what better proof that a species is a competitor than finding evidence that it has already consumed your favoured prey? This does not seem to us to require exceptional cognitive capabilities of "abstraction". From a broader point of view, there is an extensive literature on intraguild predation by carnivores. Studies and reviews show that killings preferentially target and exclude other carnivores of similar size, diet and taxonomic family (Palomares & Caro 1999. Am. Nat. https://doi.org/10.1086/501033, Donadio & Buskirk. 2006. Am. Nat.: https://doi.org/10.1086/501033), which is systematically interpreted as the exclusion of species with a similar niche, especially with a similar diet. The link with diet is sometimes explicitly demonstrated (review in Donadio & Buskirk 2006. Am. Nat.). We doubt that most carnivores need first to have direct cognitive evidence (e.g. interaction on carrion) to identify who are the competitor species to kill or exclude. In our specific study system, kites consume rabbits and their remains are often under their nests. In our view, it is difficult to think that lynxes are not aware that kites would remove valuable prey once rabbits populations have crashed. We find it more difficult to believe the opposite, from a lynx cognitive point of view.

    c2. Second argument: there are simpler explanations: lynx find rabbit remains and enter into some sort of hunting mode. If finding a dead rabbit activates a "hunting mode" that has nothing to do with competitor removal, the simplest and most biologically logical explanation is that, by finding a dead rabbit, lynx start to look for other rabbits or other ground-prey. But then, why jump up a tree to reach a nest 6-12 m high? Our idea of a lynx entering into hunting mode is starting to stare around and move around <u>on the ground</u>.

    c3. third argument: lynx have learnt to associate rabbits on the ground with food in the tree. This explanation is also problematic. As we already explained in the manuscript, an active kite nest with well feathered young must be extremely easy for a hungry lynx to detect as a source of food, without any need of rabbit cues. Kite nestlings eject their excrements out of the nest many times/day; these fall on the ground below the nest and have a pungent smell even perceivable by humans. If the objective of lynx was simply to detect kite nests as a food source by associative cues, they would have learnt to associate kite droppings

and any sort of prey remains on the ground (not just rabbits) with food up the tree.

c4. Still regarding the potential associative cues, we have provided new tests (lines 151-158) that show that there is no difference between nests with or without rabbits on the ground in the number of kite chicks in the nest and their cumulative mass (i.e. no difference in the potential food reward for lynx, which could drive the development of potential associative cues). If anything, the food reward was on average lower for nests with rabbits on the ground (lines 151-158).

c5. Reviewers #2 and #4 have suggested other explanations, but they also seem not very convincing to us, especially after the addition of the new analyses described above (see Answers 16 to Reviewer #2 and Answer 5 to Reviewer #4, on pages 20 and 24 of this letter, respectively).

c6. In the current version, we have added text on alternative explanations and their tests. We don't think that adding all the lengthy explanations reported above would be useful for the manuscript.

d. Overall, we find it difficult to think of plausible explanations other than competitor removal, but will be happy to incorporate them if the Reviewers propose them. While we retained the competitor removal interpretation, <u>in the current version we have done our best to follow the main Reviewer's suggestions and have expressed even more caution about this result. In particular, we have reinforced the careful wording in the summary; we deleted the "punishment" wording throughout; we have eliminated the sentence on the novelty of the findings; we have added the additional tests about alternative explanations, we have expressed the need for proper experimentation and explained that we cannot think of other plausible alternative explanations</u> (lines 149-158). We have left the interpretation of the results where it was (as a comment when the result is illustrated), because we consider it as an intriguing but side result. We feel that the later portion of the manuscript (main discussion) should focus on the climate-interaction relations.

e. We hope that the Reviewer does not find it offensive if we did not agree with some of the proposed arguments. <u>If the Editor and Reviewers still feel that this portion should be deleted, we will be available to do it in a further revision. However, we believe it would be a pity</u>.

6. **REVIEWER 1**: *Presentation of these results*

As a separate but related issue, I find the data presentation in Fig. 3a,b,e non-intuitive, confusing, and poorly explained. While writing the 'overview' above, I found that it was actually hard to put the result (as currently shown) into words. For starters, what exactly are those bars? For panel (a), where the rabbit variable is a continuous proportion, my instinct is that bars should be Mean (± SE) [or boxplots] % of rabbit prey across nests in each category. Even better (and possibly complementarily), the analysis would be a logistic regression with probability of lynx predation on the Y-axis as functions of % rabbit prey on the ground / in the nest, visualized with curves fit and with 'rugs' on the top and bottom X-axes to show the distribution of data points. This might require 2 panels, which would be fine. The authors might have some statistical argument against performing logistic regression given the nature of their data; that might be a reason not to include it in the manuscript, but I would still want to see it in a response letter.

Panels (3b) and (e) are more clearly contingency table analyses with pairs of binary categorical variables. Even in those panels, it is much more intuitive to have '% of nests depredated' on the Y axis, bar colors corresponding to +/- Rabbit, and bar groups for 'Ground' and 'Nest'. This would more clearly reflect the goal, which is to test the predictors lynx predation (dependent variable is conventionally on the Y-axis).

AUTHORS: We have re-arranged the figures so that the y-axis is the dependent variable and the grouping variables are re-positioned in the way requested by the Reviewer. The data are all contingency tables and we think it would be better for the reader to see directly the real data, rather than model outputs.

7. **REVIEWER 1**: *For all data figures (main text and ED), I'd like to see essential statistics in the figure captions, regardless of whether they are also in the text or not. Being able to see them in the same glance as the figures is really very helpful for reader comprehension. ED Table 1 has full model results for most of the models, which is good (it does not include the chi-squared tests for Fig. 3 which are only in the text: L128-131). Still, please add them in the captions (or the panels, if that is allowable and convenient). This is a very small redundancy in the scheme of things and it is more than justified by how much easier it makes it to understand the results, especially for casual readers. For chi-squared tests in Fig. 3, it is easy to include the full stats in the caption; for GLM results, report parameter estimates and P-values (or even just P-values) and include callouts to ED Table 1*.
AUTHORS: We added the statistics to all figure legends, as requested. We followed the example of the Walker et al. (2023) paper in Nature (the first one suggested in Comment 4 above), that seems aligned with the Reviewer's suggestion.

8. **REVIEWER 1**: *The statement of these results on L130-131 states "there was no such difference for prey remains observed inside the nest" but then states what appears to be statistical evidence that there are differences (P = 0.035; P = 0.056). Based on the chi-sq values reported, these P-values must be wrong (for a 2x2 contingency table, I get P = 0.75 and 0.56, respectively[?]). I assume that these are just typos in the P-values, because the chi-squared values match the figures, but you should re-check everything*.
AUTHORS: We have corrected these wrong figures, which were clearly inconsistent with the sentence and the figures, apologies for the mistake and our sincere thank you for pointing it out.

9. **REVIEWER 1**: *Finally: Red/orange and green is a poor color combination for colorblind readers. Even for those of us who see color, it is not pretty*.
AUTHORS: We changed the colors to more standard complementary ones, we hope the Reviewer will find them more attractive.

10. **REVIEWER 1**: *UNANSWERED QUESTIONS AND 'MISSING' DATA/ANALYSES*
Given the complexity of ecological dynamics under investigation (as the authors are the first to point out, L204), the manuscript is remarkably brief and sparing with display items in both the main-text (1 set of photos, 2 data figures) and extended data (1 map, 1 more set of photos, 1 data figure, 1 statistical table). In my view, the ratio of complexity to information content is very high. There would be no problem with presenting just 3 data figures, 1 table, and 200 lines of manuscript text if that were sufficient to establish the validity of the findings beyond reasonable doubt. However, I am left with a number of reasonable doubts and questions about issues that I think call for additional data and/or sensitivity analyses and/or discussion in the text. I do not think that any of them is unfair; they are all relevant to the reader's ability to gauge the validity of the inferences. The authors may not be able to address all of them, but they certainly can shed light on many of them.

[3a] Waterfowl data and temporal dimension. Time plays a critical role in the authors' account, which is effectively divided into 'pre-RHD' (before 2013) and 'post-RHD' (2013 onwards) intervals. The claim is that lynx-kite predation occurred only after RHD, and then only in drought years when rabbit densities dropped to very low levels. This is ostensibly because in non-drought years, lynx were able to prey on ducks and geese (L110), whereas in drought years waterfowl were unavailable, leading to kite predation. This is based on studies conducted from 1973 to 1984, showing that mallards were

the commonest prey after rabbits in the 1970s (Delibes 1980), and that consumption of ducks and geese (and birds in general) increased after a "severe drought" in 1982-83 (Beltrán & Delibes 1991). Notably, despite this severe drought, waterfowl were still clearly available to lynx. Beltrán & Delibes noted that the graylag goose population had increased from 3000 to 85000 between 1973 and 1984, list land-use change as a contributing factor, and – remarkably – seem to have anticipated the events in this study: "The lynx is capable of occasionally shifting its predation to suitable alternative prey so that in case of need (e.g., if rabbits become excessively scarce for a short period after an RHD epidemic) …"

Why this matters: The assumed role of waterfowl in lynx diets – and their lower availability during drought -- is crucial to the interpretation about drought effects because otherwise it is unclear why lynx did not prey on kites in non-drought years when rabbit abundance was as low or even lower than in the drought years. <u>Yet no information is presented about waterfowl abundance</u>, and the data cited are from ~50 years ago. Beltrán & Delibes suggest that waterfowl are actually more accessible during drought years, because they are weakened and spend more time in pastures, and because lynx are better able to access the wetland area (contrary to the interpretation implied in the study). de Felipe et al 2024 GCB present extensive data on waterbird abundance in Guadalquivir Marshes and show a sustained and dramatic decline of both herbivores (gray geese) and pre-Saharan dabbling granivores (mallard) from 1985 to 2021, which is attributed to "deterioration of the Doñana National Park natural marshes" and is not obviously consistent with the idea that waterbird availability waxes and wanes with drought. The availability of these data make the lack of any discussion of waterfowl abundance puzzling. Data on lynx movements, if available, would also be helpful for evaluating the plausibility of inferred role of waterfowl. As would any concrete evidence, from scat or observations, that lynx are preying on waterfowl in wet post-RHD years but not in dry post-RHD years.

Further, given these temporal trends and the key temporal dimensions of this work (the pre/post RHD epidemic, the inter-annual variability in drought), it seems necessary to see the data presented through time: What are the trends in rabbit density, waterfowl abundance, and drought across the study period (or from whenever the relevant data or representative proxies are available). There is no doubt that lynx predation on kites has become more frequent, but the authors present a nuanced interpretation, and temporally explicit trends over time are critical supporting evidence for that interpretation…or they may complicate the interpretation, requiring it to be revised.

AUTHORS: We understand the doubts expressed by the Reviewer and in the current version of the manuscript we have:

- added a figure that shows the temporal development of the predation peaks, together with rabbit availability and ducks' availability (current Figure 3). The figure confirms that the predation spikes occurred after 2013 and coincided with drought years in concert with troughs in duck availability.
- added an analysis of the availability of ducks (family Anatidae, the main alternative prey for lynx according to the previous papers cited by the Reviewer) during drought and non-drought years of this study. The data are based on aerial censuses conducted by plane and confirm the lower duck availability during drought years. The methods for these data have been incorporated in lines 729-742 and the analysis is shown as model c in Extended Data Table 1.

Some words of caution about the interpretation of previous papers provided by the Reviewer:

a. the article of Bertrán & Delibes focuses on the drought of the <u>spring</u> 1983. The geese data mentioned by the reviewer refer to the following winter (1983-1984) in which the marshes were starting to reflood (1984 was not a drought year), hence the higher availability of geese (i.e. it does not mean that lynx were preying more on waterbirds during drought).

b. But even independently of this, geese do not enter the "picture" of our study, because they are winter migrants. They are absent from Doñana in the spring, when lynx prey on breeding kites. We never mentioned geese in our original text.
c. In fact, the first column of Table 1 of Bertrán & Delibes supports our arguments: during drought, ducks decline strongly in the spring diet of lynx.
d. Similarly, the article of de Felipe et al. 2024 focuses exclusively on winter migrants, which are in large portion different species (e.g geese) and subject to very different trends than the breeding ducks important for lynx in spring. In the model on duck abundance that we have now added (model c, Extended Data Table 1), we use spring data and ducks' abundance does "waxes and vanes with drought" (see blue line in Figure 3).
e. Unfortunately, we do not have data on lynx diet and movement from recent years, but, as stated in point c above and in the manuscript, the importance of ducks as the main alternative prey for lynx is well shown in the cited papers, and these papers remark their importance especially in spring (e.g. 272 ducks preyed by lynx in the Delibes 1980 paper, out of 1537 droppings). Please note that, although we wish we could, we cannot study everything. During our fieldwork season, we already work full time no-stop for several months in a row, Saturdays and Sundays included. Finally, there is no biological reason to think that ducks should have stopped being an important alternative prey in recent years. Ducks are still present in the park in good numbers during years of normal inundation also after 2013 (see blue line of current Figure 3).

11. **REVIEWER 1**: *Categorization of 'drought' and temporal dimension (again). The authors categorize drought "as years in which inundation levels went below 20% of maximum flooding in February-March" (L489). Categorizing a potentially continuous variable is always a questionable decision, as information is lost and arbitrary criteria are required. In Fig. 2 we are shown seemingly very compelling trends. These are driven by 5 drought years when rabbit abundance was low; all other drought and nondrought years alike had low kite predation, regardless of rabbit abundance. Which years these were is not specified.*

Why it matters: While the R2 of these fits is high, these are still relatively small numbers of drought years. The numbers are passable given that these represent entire years of a 25+ years study. The worrying possibility is that these very tight trends are strongly influenced by the (arbitrary) criterion for classifying drought years (which again, are not show to be tied to the putative mechanism of alternative prey availability for lynx). A more convincing analysis, to me, would be to show that these trends are robust to analysis either by the abundance of waterbird prey OR by a quantitative version of the drought variable – e.g., actual % inundation instead of a threshold, or by rainfall. And again, showing trends in by year would be a highly informative complement.

AUTHORS: We have added Figure 3, which now shows more clearly which years were classified as drought. In the current version, we have also provided much more detail of how we classified a year as drought and explicitly listed the drought years (lines 592-613). We have also provided a complementary assessment that classifies years as more general "climatic anomalies", i.e. not only drought but also anomalies that may accentuate or complicate drought (severe heat in early spring that accelerates marsh desiccation, extreme rains late in spring that complicate drought even more, see the added text) (lines 614-632). This analysis confirms and reinforces the one on drought and is now shown in the Extended Data Table 2.

Regarding the usage of drought as a binary variable rather than continuous estimates, we consider it adequate for several reasons:

a. we agree that some level of subjectivity is typically required to define drought. But some level of subjectivity is also required to define which continuous variables to use instead of "binary drought" and, especially, when to measure them. For example, we re-ran the models on lynx predation on kites (models a and b of Extended Data Table 1) by fitting the interaction between rabbit density/availability and continuous underlying estimates of "drought". We tried several different options, such as the inundation level on several dates of February or March, the minimum level of inundation reached in February-March, we even tried the main axes of a PCA built with inundation levels plus extreme heat and rainfall. All these continuous indicators gave much worse model fit than simple binary "drought": compared to the model based on binary drought, all the AICc of the "continuous variables" models were higher (worse-fitting) by 10.4 - 79.0 AICc-units. Please note that, in all models with these continuous indicators, the interaction between rabbits and each continuous indicator continued to be always highly significant. Thus, incorporating these continuous estimates would not change the results, it would only lower the fit of the models.
b. for workers living in Doñana, drought is a strong biological reality with a clear dichotomous nature. In our field experience, when inundation levels drop below 20%, we experience a sort of "tipping point" where a normally blue-green environment converts into a brownish-greyish arid steppe. We notice these years especially by the sand-dust that constantly enters our eyes and covers our bodies and the paper sheets on which we write. In the current version, we have added a figure (Extended Figure 1) with pictures and maps that may help to convey this to readers unfamiliar with the Doñana environment.

11. **REVIEWER 1**: *One possibility suggested by language and data in de Felipe et al. 2024 GCB, and by other information elsewhere (some mentioned below) is that data reflect a more general breakdown of the historical status quo in this ecosystem, influenced also by land-use change and agricultural water diversion (mentioned in the ms) in addition to RHD and climate change and perhaps other factors, which is not the interpretation the authors have proposed.*

AUTHORS: Doñana is surely a complex ecosystem and, probably like any ecosystem in lowland-populated areas, it suffers from a variety of anthropogenic pressures. There is local discourse about the "general breakdown of the historical status quo of this ecosystem", but at the moment, this is more at the stage of "opinions". While we don't deny the progressive accumulation of multiple pressures on this ecosystem, in the context of the present study, if such a breakdown existed, we would expect either a gradual-progressive increase in lynx predation on kites, or, more likely, an abrupt start in lynx predation that is then sustained through the years because of a sort of tipping point-breakdown. Instead, we see up and down bursts of predation when an underlying rabbit crash acts in concert with drought, which curtails the availability of alternative prey. To us, this is sufficient to explain the observed dynamics well (Figures 2 and 3, models with $R^2 > 0.85$) without the need of invoking additional factors that, in our humble view, can only be presented as speculations. Should somebody come out with an index of "ecosystem breakdown" in the future, it would be surely interesting to fit it to the models. At the moment, all we could say is that, possibly and

qualitatively, the multiple anthropogenic pressures to which the ecosystem is subjected could have acted as a continuous underlying stressor that reinforces the rabbit scarcity (?). This would have to be presented as an absolute speculation. To be sincere, this sort of sentence would then probably have to be added to almost any ecological paper (there could always be the suspicion that some further, underlying anthropogenic factor is exaggerating almost any relationship).

12. **REVIEWER 1**: *Causal inference on the relationship between lynx predation and kite decline, and kite decline and owl increase (and temporal dimension, again). The study concludes not just a set of pairwise interactions, but a dynamic chain of interlinked (interlynxed?) events that implies a temporal sequence: First, rabbit crash; then lynx (sporadically) depredate kites; then kites decline; then little owls are released. The authors' interpretation suggests that we should see high rabbit abundance prior to 2013, followed by a decline of rabbit abundance, with intermittent droughts leading to both further reductions in rabbit abundance and reductions in waterfowl occupancy and consequent spikes in kite predation, followed by increased little owl abundance. And we should see this whether drought is categorized by threshold or shown as a continuous inundation or rainfall variable. As with the data on lynx-kite predation ~ rabbit abundance, a correlation is shown between kite abundance and owl abundance (Fig. 3f) that looks very convincing. But again, the temporal trend in this correlation is important.*
The statistical analyses (ED Table 1) test each response as a function of the putative proximal variable. For owls, this is black kite abundance, and strong support is found for a quadratic decreasing relationship ($R2=0.84$). But the interpretation supposes that the trends in little owls are ultimately a function of IGP by lynx: IGP is shown to predict kite variables, and kite abundance is shown to predict owls, but does IGP predict little owl abundance, as the interpretation suggests? The answer is not obvious, because the explanatory power of lynx predation on black kite abundance is relatively low ($R2 = 0.26$).
AUTHORS: Effectively the analysis of a more direct link between lynx predation and little owls was missing, we apologize for the omission. We have provided the additional test in Extended Data Table 1 (model m). Please note that, springing from this analysis, from this year we will start intensive data collection and prepare project proposals on little owls and other lower trophic level species, as this seems a promising avenue for future research. For the moment being, we just wanted to give a quick snapshot of the potential of the interplay between climate and predator interactions to affect third-party species.

13. **REVIEWER 1**: *In this regard, there is reason to wonder about the influence of factors not mentioned in the text. Land use and water diversion are obliquely mentioned in the text. Another factor is not addressed, which is recent work led by the lead author showing that a chemical spill in the late 1990s led to elevated levels of lead, arsenic, and other toxic metals, with very severe effects on kite reproductive success as of the early 2000s. Although that was a long time ago, it is also emphasized that kites are long-lived species (30 years). So while many of the analyses in this study are effectively controlled by comparisons between black kites in the contemporary ecosystem (presumably exposed to similar environmental effects), the kite population declines mentioned in the text could plausibly relate to bioaccumulation of toxins, rather than lynx predation alone, in which case that might partially also explain the trend in little owls (rather than lynx IGP). By the authors' interpretation, we should see kite abundance fluctuating up and down through time (not just decreasing monotonically), and little owls should fluctuate in concert (not just monotonically increasing).*

AUTHORS: The chemical spill of 1998 is unlikely to have affected the recent kite population. By when lynx started to prey heavily on kites, in the study area there were only two or three kites born in the year of the chemical spill or in the immediately following years. Furthermore, recent screenings of kites' eggs have found acceptable levels of chemical contamination, far from levels capable to cause declines.

14. **REVIEWER 1**: *Finally under this point, data on kite diets other than rabbits would be good to know. Is it possible that black kite abundance is also regulated bottom-up by the availability of prey, rather that purely lynx IGP as implied by the analysis in ED Table 1? Such data would be even more valuable given that kite diets are described inconsistently. In three separate places, using almost identical language, it is said that black kite "specializes on rabbits where these are abundant" (L75, L510, L524). This is based on two studies from Matas Gordas (I am not familiar with the location, but it is either inside or just north of PND, based on references 38, 61) and a third study from garbage dumps around Madrid (Blanco 2007, ref. 62, where the extent of rabbit predation does not indicate a high degree of specialization, i.e., 9-23% by number, 16-40% by biomass). The text emphasizes the specialization on rabbits by citing a figure of "up to 80% of the diet", which is the maximum observed in ref. 38 but struck me as an instance of selective citation that implies a considerably higher frequency of rabbits in the diet than was typically observed in refs. 38, 61, and 62 together. Oddly, kites are then described later in the Methods (L587-588) as "aggressive generalists, feeding on more than 250 prey species. Super-generalists of this kind…" These statements are not absolutely incompatible, but they paint very different implications. This is relevant in part to the plausibility of the 'punishment' hypothesis and in part to the repeated assertion that lynx and black kite are actual competitors (which entails not just prey overlap but also a negative effect on population growth rate of one species on the other).*

AUTHORS: We understand the inconsistencies. To fix them:

a. we have reported also the <u>mean</u> occurrence of rabbits in kite diet in our own study plot (58%), not just the maximum (80%);
b. deleted the citation of the study from Madrid, which effectively created confusion;
c. substituted the term "specializes on" with "preys heavily on", which may cause less confusion;
d. please note that it is extremely common (and almost standard) for birds of prey to "specialize" or have diets extremely dominated by one or a few very profitable prey taxa when this/these are abundant, but simultaneously complement the diet with a wide variety of other species. The two concepts are not really in such contrast with each other. However, in the current version we have tried to improve the coherency of the language and now we avoid the term "specialized" and "super-generalist" referred to the same species, which effectively could cause confusion in the reader (see changed language in lines 87-89, 650-652, 743-746).

15. **REVIEWER 1**: *Supporting inference based on other species. The same study by Sergio et al. 2024 on environmental contamination notes that the red kite population in Doñana "is currently in steep decline for unknown causes". Red kites eat rabbits just as frequently as black kites, if not more so (Veiga & Hiraldo, Holarctic Ecolog 13:67-71). Red kites are much less abundant than black kites, and I don't know their local distribution, but by the logic of this study, lynx should interact similarly with both red and black kites. Is there evidence that they do?*

AUTHORS: It would have been very interesting to study, but red kites have been long-absent from the study area.

16. **REVIEWER 1**: *Absolute quantitative summary/descriptive data. Data in the study are dominated by proportions. There is no problem with proportional data, but would still be helpful and complementary to be informed about the actual absolute numbers and frequencies of events. These can be partially pieced together by stray numbers in the text and on the figure axes; for example, on L633, reference is made to 108 total kite predation events. But one can only piece together a fragmentary picture. Presenting these more systematically to complement proportional data would further add to the transparency and readers' ability to be confident in the interpretation offered. Adopting the suggestion to show data (perhaps in the ED) as time series would be one way to do this, as would stipulating them in the text.*
AUTHORS: We did not add them in the text because it would be cumbersome to give long strings of sample size numbers in the Method or figure legends, but most of all because these data would be available anyway in a public repository upon publication and even as a quick and ready file that is associated with each figure. To give a better idea of the sample sizes involved, in lines 774-776 we now report the average and range of nests checked annually. To give you a rough idea, during the lynx predation peaks, the annual sample sizes ranged between 54 and 66 checked nests and 18 to 25 predated nests. These are considered good sample sizes for a nest-based raptor study.

17. **REVIEWER 1**: *A number of words and phrases are employed that I think should be revised. — e.g., L. 17 - "peaceful coexistence": Peaceful is a human construct that seems inapplicable (1% of which were killed by lynx during the period of 'peace'). "Neutral" would be better, as is the description on line 104 of how the species "basically ignored each other")*
AUTHORS: we have changed "peaceful" to "neutral", as suggested.

18. **REVIEWER 1**: *— e.g., L20, L68, L500, and throughout: "Lynx and kites compete for shared prey": what is the evidence for competition, besides shared prey (which is not sufficient basis alone to assume competition). For example, if kites are not eating enough rabbits to have a resulting negative effect on lynx population growth rate, or vice versa, then they are not actually competing event though they share pray. You could say "likely compete" or "potential competitor" or similar.*
AUTHORS: we have now used the suggested, more cautious phrasing.

19. **REVIEWER 1**: *— e.g., L. 33, L189 - "climate and biodiversity crisis": The word 'crisis' is a subjective characterization (cf. "disaster", "catastrophe", "cataclysm") and dubiously appropriate for a peer-reviewed primary research article about lynx—kite IGP. It is established that the climate is warming, but there is not a scientific consensus, and indeed much debate, as to whether this is appropriately described using terms such as 'crisis' Similarly, it is established that rates of biodiversity loss and population extirpation are accelerating and higher than plausible estimates of the background extinction rate, but here there is even less consensus as to the appropriateness of terms such as 'crisis'. The subjective use of such terms is criticized as politically motivated exaggeration by people on both the far right of the political spectrum (mainly with respect to climate change) and people on the far left (mainly with respect to biodiversity loss, when in tension with human livelihoods). This is one of the factors that is contributing to the erosion of scientists' credibility and the loss of public trust in science.*

AUTHORS: we have deleted the term "crisis" throughout, thank you for pointing this out.

20. **REVIEWER 1**: *— e.g., L168 - "worrying": for the same reasons as "crisis". Worrisomeness is not an objectively measurable quantity.*
AUTHORS: we have deleted the term, as suggested.

21. **REVIEWER 1**: *— e.g., L1, and in dozens of other places throughout - "climate change": the word 'change' implies that the droughts in this study are attributable to climate change per se, as opposed to merely being anomalous years; it is certainly plausible, indeed likely, that it is influenced by human GHG emissions, but that not known with certainty. It is appropriate to refer to 'climate' throughout; and it is appropriate to say what models forecst(although note that climate model forecasts vary a lot in confidence and precision, depending on both the specific model and the geography). It is also even appropriate to describe concerns about the impact of climate change on IGP as a motivation for the study. But attributing the drought and ensuing interaction dynamics to climate change, per se, goes too far, notwithstanding the suggestive climatic trends briefly mentioned in the Methods.*
AUTHORS: We understand the cautiousness of the Reviewer, but we would prefer to keep the current wording. We really don't see how drought could not increase in the future when the temperature is locally increasing by 0.5^{o}/decade, regional analyses forecast future increases in drought frequency and severity, and when the observed trends in climate are so often faster and more severe than forecasted. We believe that the implications of the study in terms of climate change should be explicit from the start.

22. **REVIEWER 1**: *L1 – Title: Remove the word 'change' or reword with a better alternative (e.g., drought [most precise] / extreme weather event / climate anomaly. Beyond that, I think the word 'disease' must also be in the title. Ultimately, as you show, these are not effects of climate effect per se, but rather an interaction between climate and a rabbit disease, with both being necessary and neither one sufficient to trigger the dynamics.*
AUTHORS: We thank the Reviewer for the suggestion. We thought of the various suggested alternatives and also other ones, but we believe that they would weaken the title and make it less attractive and general. For the moment being, we have retained the original title, because it is the one that, in our humble view, would best convey the results of the study and maximize attention.

23. **REVIEWER 1**: *L25-28 — I suggest a rewrite of these two sentences that summarizes the significance of the contribution without relying on so much jargon. See larger comment above about this.*
AUTHORS: We already changed the first sentence according to the suggestions of the Reviewer (see answer to Comment 4 above). We also changed the second by trying to avoid jargon.

24. **REVIEWER 1**: *L102: What are the 1662 nests? Nest*Year combinations? (N = 105-223 kite pairs in the study area)*
AUTHORS: yes, we have better specified that it is the cumulative number of nests.

25. **REVIEWER 1**: *L115: Are these percentages and cohort stats for a particular year or years? Which ones?*
AUTHORS: This is now illustrated in the newly added Figure 3.

26. **REVIEWER 1**: *L142: First demonstration of the fitness consequences of IGP on vertebrate predators — are you sure about that?*
AUTHORS: We are not aware of other studies that tested impacts on comprehensive estimates of fitness such as lifetime reproductive success. We have added "to the best of our knowledge" in case we missed any study. If unacceptable, or if the Reviewer knows about studies that we may have missed, we will be ready to remove the novelty claim during the next revision.

27. **REVIEWER 1**: *L145: "climate change" again*
AUTHORS: we deleted the term.

28. **REVIEWER 1**: *L161: Heightened predator competition — this seems like speculation*
AUTHORS: We rephrased the sentence to avoid the competition-speculation.

29. **REVIEWER 1**: *L162: Strong bouts of lethal predation "never observed before" — 1% of kite nests were predated before rabbit declines (earlier in the ms)*
AUTHORS: "never observed before" refers to "strong": <u>strong</u> bouts of predation were never observed before.

30. **REVIEWER 1**: *L171: "Comprehensive strategies tackling multiple anthropogenic threats". This might sound reasonable at first glance, but on scrutiny, it is a vague and impractical recommendation; it states that all anthropogenic threats must be "tackled", which is unrealistic as a management objective*
AUTHORS: We humbly disagree, we stated "multiple" not "all" anthropogenic threats. The concept is necessarily general. There will be cases in which multiple threats can be managed (human persecution, electrocution, unsustainable harvest of prey, etc), and cases in which it will be challenging or impossible. We have better specified that the suggestion applies to cases in which this is feasible (line 206-207), but, as a general message, we believe we should retain the general optimism invoked by the Reviewer. It is also more in agreement with the now incorporated requests by Reviewer #4 (Comment 7).

31. **REVIEWER 1**: *L186: Avoid using "evolve" to avoid confusion between evolution and behavioral plasticity*
AUTHORS: we changed "evolve" to "develop", thank you for pointing this out.

32. **REVIEWER 1**: *L69 and subject heading on L499: Change 'model system' = Drosophila, C. elegans to 'study stem' or swell studied system'. For the subheading on L499 up might change to "Trophic modualg*
AUTHORS: We have changed the terms as suggested (lines 82 and 641).

33. **REVIEWER 1**: *L502: "that compete.."*
AUTHORS: This had already been changed according to the indications of Comment 19 above.

Overall, the revisions requested by Reviewer #1 have required several weeks of work, re-analysis and study of the proposed additional papers. We hope that the Reviewer will appreciate that we did our best to follow the suggestions.

COMMENTS BY REVIEWER 2:

1. **REVIEWER 2**: *The manuscript "Climate change precipitates carnivore predation on raptors" investigates the effects of two serial stressors (disease and drought) on a food web in Doñana National Park. It shows that after disease reduces a common prey (rabbits), during drought lynx prey on black kites.*
Although this area is definitely less well investigated, I do not agree with the claim that we know virtually nothing about interactions among vertebrate top predators under climate change, and a quick search brings up a variety of papers on apparent competition, predator-prey and population dynamics (e.g., https://link.springer.com/article/10.1007/s00442-017-4017-y). Perhaps this might be better phrased, as "direct interactions" which I do agree with, as intraguild predation is more usually noted as a one-off phenomenon in studies and does warrant more focus.
AUTHORS: We have changed the claim to "direct interactions" (line 38), as suggested by the Reviewer. Thank you.

2. **REVIEWER 2**: *I also do not find the interpretation of the results, "a punishment arms-race" to be particularly compelling, and without further mechanistic details, feel that there are a wide range of potential explanations (see specific points below). Although this is certainly one potential explanation, I feel that the authors do not offer any alternatives which is slightly misleading*.
AUTHORS: We have deleted the term "punishment" (see more below; see also answer to Comment 5 by Reviewer #1, on pages 6-8 of this letter).

3. **REVIEWER 2**: *I found the language was sometimes a little unscientific and distracting (e.g., peaceful coexistence, when simply coexistence would suffice, or punishment of the intraguild competitor). These instances tend to anthropomorphises the interacting species and, in my opinion, reduces the clarity of the narrative. It would be better to use only well-defined and common ecological descriptors of the interactions to keep consistency with the wider literature. Along these lines some of the sentences are very long and winding and could be more succinctly written, for example in the abstract the third sentence is 56 words and 6 lines long, and could be split into 2-3 sentences*.
AUTHORS: We have rephrased and avoided terms like "peaceful coexistence" and "punishment" throughout, as also suggested by Reviewer #1. We have also split the long sentence into two sentences, as suggested (lines 13-18). We agree that the sentence was too long and is now more digestible, thank you.

4. **REVIEWER 2**: *The methods appear robust, however, based on the definitive statements made in the conclusions about "punishment" of competitors, I think there should be more detailed information on the diets of both the predators in order to drive towards some more complete mechanistic understanding. To me, there are quite a few confounding variables in the field study, which means that it is not possible to be confident in the proposed explanation for the observed patterns.*

AUTHORS: We have now added data on the availability of the main alternative prey (ducks) and we believe that this should strongly improve the support for the proposed interpretation (see answers to Comments 18 and 19 below).

5. **REVIEWER 2**: *Specific points:*
Abstract: Line 15-20: This sentence is very long. Consider splitting into 2-3 sentences.
AUTHORS: We have split the sentence into two separate ones, apologies for the previously convoluted one (lines 13-18).

6. **REVIEWER 2**: *Line 18: I would use the Latin binomials to be more specific. Also, you define some taxa but not all. For example you don't say that the predatory raptor is a black kite, or what the insectivorous bird is on line 23.*
AUTHORS: We have now added the names of the species involved everywhere. We were trying to avoid Latin binomials to save the limited space for the summary, remain more general and minimize text "interruptions". But we can add them during the next revision is really deemed fundamental.

7. **REVIEWER 2**: *Line 24: I would always be very careful about this. It is highly likely that there is already evidence of this to date, and that it is not the focus of the paper. I would rephrase to not say "first" but instead focus on the novelty. I.e., "These results demonstrate a rarely observed climate-induced intraguild predation effect that…".*
AUTHORS: This sentence has already been deleted to follow the suggestions of Reviewer #1.

8. **REVIEWER 2**: *Line 26: This is a little vague and is not necessarily specific to what you found here – try to specifically link the changes in food webs you observed and make a more specific statement that still speaks to this wider point.*
AUTHORS: We changed the sentence in the direction suggested by the Reviewer (lines 23-27). We hope we succeeded.

9. **REVIEWER 2**: *Line 32-33: Why are they recognised?*
AUTHORS: If we properly understand what the Reviewer is asking, this is explained in the following sentence.

10. **REVIEWER 2**: *Line 38-39: I don't agree that this is the case, there is a lot of information around the impacts of climate change on species interactions: see this review from 2008* https://onlinelibrary.wiley.com/doi/full/10.1111/j.1461-0248.2008.01250.x.
AUTHORS: Yes, we agree, the reported link is the first paper that we cite in the manuscript, i.e. the first that appears in our Reference list. We did not write that "there is scarce information around the impacts of climate change on species interactions". We had acknowledged such concept in all the previous portion of the paragraph, after which we started to narrow down the argument to the fact that: "…despite many recent advances for certain taxa and ecosystems [e.g. 14-16], we have very scarce knowledge of the interplay between climate and <u>some key</u> interactions". This then leads us to stress the lack of knowledge about interactions among top predators.

11. **REVIEWER 2**: *Line 47-50: Very long sentence*.
AUTHORS: We split this into two sentences, thank you (lines 60-63).

12. **REVIEWER 2**: *Line 75: Why is the longevity of the lynx also not provided? Why is it not relevant?*
AUTHORS: apologies for the omission. We added the longevity, but we have found trouble finding a reliable source. There are mentions of longevities up to 13 years on the internet, but only up to 11 years in scientific papers. For now, we have reported 11 years to stay on the safe side.

13. **REVIEWER 2**: *Line 75: Is it specialisation if they are abundant? I would rephrase this as it is a little contradictory – if they specialised on rabbits this would not just be when they were abundant.*
AUTHORS: The "specialization" phrasing has been deleted, also in line with the comments by Reviewer #1 (line 87-89).

14. **REVIEWER 2**: *Line 78: How representative is Doñana of the wider ecosystems? I.e., how generalisable are these results to lynx and kites elsewhere on the Iberian peninsula? Especially considering it is a wetland?*
AUTHORS: We are not sure we understand. We do not see why the impact of anthropogenic drivers on the interactions among predators should be different in other areas. We understand the interest in the situation of the wider Iberian peninsula, but maybe this level of detail when presenting the study system would be more pertinent for a more specialized zoological or ecological journal (if we properly understood the comment).

15. **REVIEWER 2**: *Line 108: This is very non-specific – try not to use phrasing like "ignored each other" it is confusing and misrepresents the structure of ecosystems (e.g., the presence of these taxa will affect behaviour and other aspects, so they do not ignore one another if they are not directly interacting).*
AUTHORS: We rephrased the sentence to state that they were "neutral" to each other, as suggested by Reviewer #1 (line 118).

16. **REVIEWER 2**: *Line 132: Or is it because these nests are more conspicuous, or that there will be more chicks on that nest (requiring more food) and therefore more energetically efficient. I am not convinced that the evidence you present points towards the fact that this is predation to reduce competition*.
AUTHORS: We are not sure what the Reviewer means by the fact that the nests with rabbits under them would be "more conspicuous". All nests in this analysis have remains under them and all have large nestlings, implying a substantial quantity of white excrement with a pungent smell under the nest, i.e. there were more simple and reliable ways to detect that a nest contained chicks. Regarding the number of chicks in the nest, we have added a test that shows that there was no significant difference in the number of kite chicks between nests with or without rabbit remains on the ground (lines 151-158). If anything, there were less chicks on average in nests with rabbits on the ground. There was also no difference in the cumulative mass of the brood contained in the nest, i.e. no difference in the potential "food reward" for the lynx decision to climb and predate the nest (lines 151-158). Again, if anything, the food reward was on average lower for nests with rabbits on the ground.

Please note that, for the moment being, we have retained the "competitor removal" interpretation and added the test of alternative explanations. <u>If the Editor and Reviewers still</u>

feel that this portion should be deleted, we will be available to do it in a further revision. However, we believe it would be a pity.

17. **REVIEWER 2**: *Line 122: Use spaces between values and SI units*
AUTHORS: We added the spaces.

18. **REVIEWER**: *Line 546: Although you know that lynx predate nests based on the described methodology you do not know what else they are eating, and as a result do not really understand the drivers of these predation events. Without diet data for lynx, it is my opinion that there can be very restricted and quite suppositional interpretation of these data. Faecal meta-barcoding, hard parts analysis or other information on lynx trophic interactions during drought and non-drought periods are needed for the conclusions made at the end of the manuscript*.
AUTHORS: As we explained in the text and methods, our sequence of thoughts and hypotheses is the following:

a. past knowledge on the diet of lynx in this population shows that it is extremely dependent on rabbits, but can diversify to a certain extent to include alternative prey (references [54-55] of the manuscript, based on thousands of scats).
b. When this happens, ducks are (by far) the primary alternative prey.
c. There is no biological reason by which the analyses of lynx diet of previous papers should have changed, as ducks are still abundant in non-drought years and there has been no sudden increase in any other potentially profitable alternative prey that we can think of.
d. In the scenario of the current rabbit crash, we hypothesized that alternative duck prey would be available in normal years but not in drought years, leading to heightened interactions between lynx and kites, which is what we found.
e. In the current version, we have incorporated data on ducks' abundance that confirm these dynamics (Figure 3 and model c of Extended Data Table 1). We believe and hope that this should be enough to fill the major gap that the Reviewer was missing. Ducks' availability is the key variable also invoked by Reviewer #1 and #3.
f. Figures 2 and 3 and their corresponding models in Extended Data Table 1 fit very well the above scenario. In our view, this is sufficient to support the interpretations. We strongly believe that additional data on lynx diet would not change the overall picture.
g. We agree that, ideally, faecal meta-barcoding and other analyses of lynx diet with and without drought would have been desirable to make the study more elegant. Ideally, 30-years surveys of every potential alternative lynx prey would have been desirable, but we cannot collect data on everything. The current study is already extremely expensive and physically and logistically demanding. We are already at the limit of what we can financially and logistically do.

19. **REVIEWER 2**: *Line 587: The densities of other prey taxa also need to be monitored to truly understand what is going on in this system, especially if the kites are super generalists*.
AUTHORS: We apologize, but, respectfully, it is unrealistic to propose that we should have surveyed more than 250 taxa for 30 years in order to have perfect knowledge of any thinkable aspect of the system. By this logic, close to 100% of ecological papers should not be published. We believe that, with the addition of ducks' abundance, we have now captured the key players of the process at play.

20. **REVIEWER 2**: *Line 626: But this higher availability of rabbit prey would also mean higher numbers of rabbits under the nests of the kites though? This is a confound with the findings, as although there are more rabbits for the lynx there are also more likely to be more rabbits for the kites and therefore have more rabbit remains under the nest*.

AUTHORS: This is an interesting idea, thank you. Because previous analyses had found higher rabbit abundance close to the marshes and these would also hold alternative duck prey, we thought that this could deter predation. The Reviewer notices that the opposite prediction could also be true, because of higher presence of rabbit remains under the nest. None of the two is then supported by the results (no effect of proximity to the marshes on predation probability). To incorporate both possibilities, we have now stated more generally that we tested whether marsh proximity could affect the likelihood of predation, without specifying anymore the direction of the expectation (line 723-724).

21. **REVIEWER 2**: *Line 645: Is there ontogenetic shift in the feeding of kite nestlings as they grow. As to my mind, you can't separate out the fact that larger nestlings will be eating more (and also maybe more rabbits) thus forming a better meal for a lynx, from the fact that the lynx might want to "punish" that competitor*.
AUTHORS: We apologize, the Reviewer was rightly confused by our lack of clarity. We had assumed it was clear (but in re-reading it was absolutely not) that these nests came from the same sample of the analysis of the previous paragraph (lines 804-806). This means that the comparison is between nests with chicks of similar age in the nest. We have now better specified the comparison-design and avoidance of such biases in lines 804-806 and 813-816. We have also more explicitly tested whether nests with rabbit remains on the ground had larger brood sizes or larger cumulative mass of chicks in the nest (= a larger food reward) than nests without rabbit remains on the ground: both tests are not significant (lines 151-158). If anything, on average, nests with rabbits on the ground are associated with a lower food reward for lynx. Sincere apologies for the confusion.

21. **REVIEWER 2**: *Figure 2: What is "normal" I would replace this with "non-drought period"*.
AUTHORS: If possible, we would like to retain the term "normal", because it better conveys that these are the more frequent, background conditions and that drought is the anomaly. But we can change it during the next revision is really deemed inappropriate.

22. **REVIEWER 2**: *Extended Data Figure 1: The fact that there are far fewer nests also in drought periods is not made clear in the main text. This is an important factor to consider when thinking about interactions. There may be less rabbits, but also less of everything else, and therefore there is an overarching increase*.
AUTHORS: Yes, this is the logic behind the idea of testing the <u>generalized</u> impact of "drought". In the specific case of the current system, because previous work so clearly identified ducks as the alternative main prey, and because ducks as waterbirds would be likely affected by drought, we focused strongly on ducks in our interpretation. In our case, we can demonstrate that with drought there are less rabbits and less ducks. That there is less also of other even minor prey is likely, but it would have to remain as a speculation and we doubt that it would add much to the processes of Figures 2 and 3.

COMMENTS BY REVIEWER 3 (Dr. Briana Abrahms):

1. **REVIEWER 3**: *This was an excellent and important study showing the dual affects of disease and drought on intraguild predation, with cascading consequences for individual fitness, population dynamics, and species interactions. Using three decades of monitoring data, the authors showed that lynx increased intraguild predation (IGP) on kites during drought periods following a disease-driven population decline in their shared prey, European rabbits. The increase in intraguild predation led to reduced lifetime reproductive success and population decline among kites. In turn, it also led to*

increased predation by kites on little owls. Such results provide important insight into how anthropogenic impacts cascade through food webs.
This was a well-written and well-designed study for which I find little to critique. In accordance with Nature review practices, I confirm that the statistics are sound and that error bars and CIs are appropriately defined in the figure captions. I am excited for its publication so that I may cite it and share it with my lab members and collaborators.

AUTHORS: We heartedly thank Dr. Abrahms for her enthusiasm about our study. It really means a lot to us.

2. **REVIEWER 3**: *My only critique is that the mechanism for which the increased IGP during drought is interpreted is that alternative prey sources are scarce during drought, forcing lynx to prey on kites. The corollary during non-drought years was that "lynx likely compensated the rabbit scarcity with abundant alternative prey" (Lines109-110). However, no data or support is provided for this interpretation, yet the explanation for why IGP would increase during droughts hinges on this. Can the authors provide any data or analysis demonstrating drought's effects on alternative prey availability?*

AUTHORS: We have now incorporated data on the availability of key alternative prey (ducks) and provided evidence that ducks abundance does decline strongly with drought (blue line in Figure 3 and model c of Extended Data Table 1).

COMMENTS BY REVIEWER 4:

1. **REVIEWER 4**: *The study is a compelling illustration of how one stressor can amplify the effects on another, with cascading effects throughout an ecological community. The study thoroughly assesses both demographic and community responses among predators to the combined effects of drought and disease on rabbit populations*.

AUTHORS: Our sincere thanks to the Reviewer for the appreciation of our work.

2. **REVIEWER 4**: *One potential concern is the small spatial extent of the study. Given the 10 km2 study plot (just over 3 x 3 km), is it possible that the effects observed here are quite limited with respect to the entire population of kites, owls, etc.? It would be helpful to know some more information about the distribution of the kite nests within the greater population and if they are particularly concentrated in this study plot for some reason*.

AUTHORS: The kite population of Doñana is distributed over a larger area. Its density is always high within 1 km of the marshes, but varies continuously in space, with areas in which it is more concentrated at higher density, such as the current study plot. There are other portions of Doñana with similarly high densities. So, we do not see the study plot as particularly unusual or unrepresentative. If needed, data on kite and owl densities are available for a slightly wider area.

3. **REVIEWER 4**: *A suggestion: An illustrative representation of all observed indirect responses to the drought-disease interaction would be very valuable (rabbit declines, increased lynx predation on kites, decreased kite abundance and reproductive success, increased owls?, decreased alternative prey?) – a sort of graphical depiction of all the results. This would allow the reader to clearly see the main conclusions, and it could also be used in a graphical abstract.*

AUTHORS: We were already planning such a graphical depiction, but found it a bit challenging to devise it in a manner that is not actually confusionary, given the many actors/processes involved. We have attempted such a graphical scheme in the current

Extended Data Figure 5 and Extended Data Figure 6. We placed two potential options. Personally, we would retain only Extended Data Figure 5.

4. **REVIEWER 4**: *Specific comments:*
Line 117 (and elsewhere, e.g. Fig 3): I'm not sure I would call these control nests, as that could imply an experiment. Referring to these as "unpredated" nests seems sufficient and more accurate to me.
AUTHORS: We have converted "control" to "non-predated" throughout.

5. **REVIEWER 4**: *Lines 132-137: I agree that one explanation for lynx predating on nests with more rabbit remains is an attempt to reduce competition. A more likely explanation I think would be simply that it is a stronger indicator of an active nests that could be predated. I would be careful not to over-interpret the 'mindset' of the lynx. It would actually be very valuable to know what other prey sources were available during these periods – were the lynx consuming the only reliable prey left, or specifically selecting for their competitors? Any information about abundances of alternative prey should be included.*
AUTHORS: We thank the Reviewer for the input. In this comparison, all nests have chicks in the nest and all nests have prey remains under the nest (rabbits or not), so all nests are active, with rabbits or not. So, the fact that lynx could associate rabbit with a higher likelihood that the nest is active does not explain the observed pattern. Also, as we explain in the text (lines 140-143) and in answer to Comment 5 by Reviewer #1 (pages 6-8 of this letter), it would be much easier for lynx to know that a nest is active based on fresh nestlings' excrements under the nest, which are very evident (pure white) and have a distinctive pungent smell. Finally, we have now incorporated additional tests that show that rabbit presence under the nest is not associated with more or fatter chicks in the nest (i.e. a higher reward). The difference is not significant, and, if anything, in the opposite direction (on average, higher food reward associated with nests without rabbit remains under the nest).

6. **REVIEWER 4**: *Line 147: Did kites move to another territory in this same study area or out of the study area entirely? If they left the study area and could no longer be monitored, this could change the inferences about lifetime fitness (perhaps animals that left found better conditions).*
AUTHORS: This analysis focuses on individuals with PVC rings. These individuals receive extremely intensive monitoring over a much larger area of over 500 km^2. We look for them everywhere and the detectability of breeders is extremely high, even when they change territory. Both sexes in this population are extremely philopatric and emigration is unknown despite more than 4000 individuals ringed as nestlings, and as also confirmed by satellite and GPS-tagging of some 200 individuals. So, the probability that lifetime reproductive success could be biased by emigration is extremely unlikely.

8. **REVIEWER 4**: *Lines 161-172: While I agree that more work in this area is much needed, this paragraph seems to ignore a fairly rich literature on multiple stressor effects and synergistic effects. Some framing within this literature would better situate this study within what is already known. Some citations provide below.*
AUTHORS: The Reviewer is absolutely right, apologies for not properly acknowledging such previous work and thank you for pointing this out. We have rephrased the paragraph to acknowledge such work (lines 190-198).

9. **REVIEWER 4**: *Fig. 2: I understand the argument that the amount of rabbits in the kite diet is a proxy for availability, but it seems to me that density is a much better one. I would have the x-axis in panel A read "Proportion in kite diet" to more accurately depict the data. I might also change the y-axes to say "Lynx predation rate on kites", as the reader may not read the legend carefully and the plot alone looks like it would be referring to predation rate on rabbits. Finally, it seems here that there are some drought years with high rabbit abundance. Were those years pre-disease? It could be useful to color the dots by if they were pre- or post- diseases to show the relationship between disease and rabbit decline. One minor stylistic point: I would recommend not using the default ggplot aesthetics.*

AUTHORS: We have changed the y-axis to "Lynx predation rate on kites", the Reviewer is absolutely right. The information on high rabbit densities in some drought years is now visible in the newly incorporated Figure 3. We changed the aesthetics of the ggplot-default to the more classical white background, as requested. If possible, we would prefer to retain "rabbit availability" in the X-axis of Figure 2, we think it is more elegant and the legend clearly states what it represents.

10. **REVIEWER 4**: *Fig 3. In general I would prefer more descriptive axes – e.g. in A) percentage of rabbits as kite prey items as y-axis, in-fig legend as predated by lynx vs. not predated by lynx. This would really help the figures to be more easily digestible. Also, what is the difference between "predated" and "suffered intraguild predation" (panels A,B,E vs C,D)?*

AUTHORS: These figures' axes have been changed according to indications from Reviewer #1 (see answer to Comment 6 by Reviewer #1, on page 8-9 of this letter). In the previous version of the manuscript, we had not placed "predated vs non-predated" (as elsewhere in the color-legend of panels C and D), because these panels refer to the lifetime reproductive success of <u>individuals</u> and we were worried to convey the wrong idea that the adult individual was predated by lynx, while it is its brood. We have now changed the color-legend so that it is the same as elsewhere and added a note at the end of the figure legend to state that, for these panels, predation refers to the brood of an individual, not the individual. We hope that this will cause less confusion.

10. **REVIEWER 4**: *Extended Data Figure 1, Line 938: Remove "of" after "myriad".*

AUTHORS: We removed it, thank you.

We thank the Editor and Reviewers for all their work on our manuscript, which we sincerely appreciate, and we hope to have clarified and improved all the issues raised. We believe that, thanks to all the suggestions, the current version of the manuscript has improved in clarity, completeness and solidity.

We are a bit worried by the sentence of the Editor that "*we would be happy to consider a revised manuscript (unless something similar has been accepted at Nature or appeared elsewhere in the meantime)*", so we (shamelessly) ask the Reviewers for patience and mercy and hope they will appreciate the many weeks of intensive work required to tackle all the issues raised.

Best regards,
Fabrizio Sergio & co-authors

Dear Dr. Armstrong,

Thank you for the additional comments by yourself and the Reviewers on our manuscript, now newly titled "Drought and disease trigger lethal interactions among top predators", as a slight modification to suggestions by yourself and Reviewer 1 (please see below Answer 3 to the Editor). We are glad that the Editor and Reviewers continue to be interested in the manuscript and have made several appreciative and constructive comments about our revision work. We have done our best to implement all the additionally suggested changes.

To make things easier for the Editor and Reviewers, we have written *in italic* the comment of the Reviewers preceded by "REVIEWER" and in standard-text, our response preceded by "AUTHORS". Please note that for some comments by the Reviewers and our answers, we have underlined some portions of the text in order to draw attention to relevant aspects of it.

COMMENTS BY THE EDITOR (Dr. Anna Armstrong):

1. **EDITOR**: *Your manuscript, "Climate change precipitates carnivore predation on raptors", has now been seen by the original four referees, and a new referee, referee 5, brought in to assess the inundation mapping (it was clear after the last round of review that we needed someone to assess this). You will see from their comments below that they continue to find your work of interest. That said, some important points are raised. We are very interested in the possibility of publishing your study in Nature, but would like to consider your response to these concerns in the form of a revised manuscript before we make a final decision on publication.*
AUTHORS: We are glad that the Editor and Reviewers found the manuscript very interesting. It would be an honour to publish again Nature.

2. **EDITOR**: *We therefore invite you to revise your manuscript taking into account the referee comments. For publication in Nature we will you need to follow the advice of referee 5 regarding the inundation mapping, and provide more transparency around the methods and data here. We also ask that you take seriously their question about whether drought is the correct terminology. It may be sensible to bring on board a referee with specific expertise in remote sensing and GIS, as referee 5 suggests, to ensure that this aspect of the study is as rigorous as possible. It may also be important to bring in data from Landsat 8 and Sentinel-2 to strengthen this aspect of the study. Beyond this, we ask that you follow carefully the referees' advice about the presentation of the work, and take out any statements that deviate from or overstate the findings. Please also ensure that the limitations of what can be concluded in the absence of, for example, dietary data, are made clear (see the comments of referee 2).*
AUTHORS: We tried to implement all suggested changes and clarify all doubts (see detailed answers below).

3. **EDITOR**: *TITLE: Titles cannot exceed 75 characters (including spaces); they must not contain punctuation. A variant of the title suggested by referee`1 could work, "Disruptive influence of drought (or low surface water availability, depending on your response to referee 5 regarding terminology) and disease on top predator interactions".*
AUTHORS: If possible, we would like to propose a slight variant: "Drought and disease trigger lethal top predator interactions". <u>If this is not liked, we'll be happy to change it to the title proposed by the Editor</u>.

Finally, please note that we waited to reduce the text and references, which would be easily done. We would first prefer to be sure that the Referees are fine and happy with the integral text.

COMMENTS BY REVIEWER 1 [redacted]

1. **REVIEWER 1**: *I want to especially highlight my appreciation for the responses (to me and other reviewers) that expressed principled disagreement. There is no need to apologize or be tentative about that (and I will not be tentative about continuing to disagree on a few points, below). Overall, I find the responses largely convincing with respect to the primary concerns articulated by me and the other three reviewers*.

AUTHORS: We are glad that our revision work and responses were considered satisfactory.

2. **REVIEWER 1**: *Title. I have read and considered the authors' responses to my concern about "climate change" in the title. I still disagree, rather strenuously, and as expressed prevoiusly, I think it is critically important for the scientific community to be disciplined about this. The study is about a chain of events precipitated by an interaction of drought and epidemic disease. Climate change is associated with droughts (along with essentially every other kind of weather phenomenon) but they are not the same thing. I am not suggesting that the authors cannot mention climate change or discuss it in relation to their study. I am not taking a position on whether droughts in this ecosystem are the result of anthropogenic climate change or not. My position is that the current title of the paper -- "Climate change precipitates carnivore predation on raptors" (a declarative statement of cause and effect) -- misrepresents the contents of the paper and the scientific advance it presents. It is imprecise (climate change is many things, including droughts, deluges, typhoons, record high temperatures, record low temperatures, etc.) and simultaneously narrow (it omits the critical role of disease). The authors' response to my previous comment -- "we really don't see how drought could not increase in the future when the temperature is locally increasing by 0.5/decade" -- acknowledges the assumption being embedded. So I think it is a poor choice even leaving aside concerns about how nonscientists and the media are likely to misinterpret it.*

Ultimately I do not understand the authors' rationale for clinging to this formulation. Phrases like "extreme weather" and "drought" will catch just as many eyes without crossing into this gray area: "Disruptive influence of drought and disease on interactions among top carnivores/predators"

To me, that is an even more compelling title that also more fully and accurately tells the reader what the study about. In that formulation, and many others, climate change is only just below the surface and will be unmistakeable to your primary audience. Reporters will ask you about whether this has anything to do with climate change, and you can say whatever you believe best serves the public interest. And nobody will be able to accuse you of having been slippery with words or misleadingly pushing a "climate change agenda".

AUTHORS: We now better understand the concerns expressed by the Reviewer. We have followed the Reviewer and also the Editor's conceptual suggestion, and changed the title to a slight variant of the proposed one: "Drought and disease trigger lethal interactions among top predators". If this is not liked, we'll be happy to change it to the title proposed by the Editor and Reviewer.

3. **REVIEWER 1**: *The first sentence of the summary paragraph is OK with me (but substitute 'increasingly' for 'growingly'). But for all of the same reasons, I would change at least the 2nd mention of "climate change" in the summary paragraph to "extreme weather events" or "climatic perturbations" or similar*.

AUTHORS: We have changed both aspects of the text, as requested.

4. **REVIEWER 1**: *23. "In the future, synergistic effects of multiple...".*
AUTHORS: We changed the text as suggested (line 24).

5. **REVIEWER 1**: *24. "keystone species" -- this to me implies that all top predators are keystone species, which is simply not true. I don't see the need for this term, which opens up a whole box of worms. The word keystone appears three times in the summary paragraph alone. See comments above about climate change. I continue to sense that the authors are trying to 'sell' the study by using terms that they consider flashy, and I continue to advise them to temper that instinct. The study is plenty flashy enough; it doesn't need any extra dressing.*
AUTHORS: We have changed the emphasis, deleted the implicit assumption of all top predators being keystone species (which was not our intention) and only retained the term “keystone” once in the summary paragraph.

6. **REVIEWER 1**: *See also "virtually nothing" (line 37; --> "little"), "urgent" (line 51; --> "There is a need"), "iconic" (line 54 --> "One common interaction among predators"), etc. If the authors could just tone it down a bit, throughout, the whole thing will come across more convincingly. We all make up our own minds whether a study is interesting and important or not; insisting too breathlessly on these things only tends to make us suspicious.*
AUTHORS: We changed the text as suggested (lines 36, 49 and 52).

7. **REVIEWER 1**: *This advice extends the "competitor reduction" interpretation -- the authors have done an adequate job of flagging this issue (a concern identified by 3 of the 4 original reviewers), but I think they could still push it even less, with stronger net effect. If it were my paper in review at Nature, I would introduce it like this:*
"One interpretation of these results is that lynx are systematically removing competitors during potential starvation years. We find this interpretation plausible -- and in fact probable -- for the following reasons 1, 2, 3 ... An alternative hypothesis is A, but that wouldn't explain B or C. Another possibility is X, but X doesn't explain Y or Z. [i.e., acknowledge and proceed to convincingly rebut the lines of argument brought by Reviewers 1, 2, 4]. Ultimately, we find the first interpretation to be the most plausible, although additional manipulative experiments will be required to confirm it." I advise doing it that way.

When I read something like that -- OR LIKE THE WAY YOU EXPLAINED IT TO ME IN THE RESPONSE LETTER -- where the force is carried in the impeccable logic rather than the 'hard sell', I think "These authors are not trying to beat me with their sexy interpretation, they are presenting the evidence and counterarguments, and the evidence and arguments makes sense. I respect the calmly confident and incisive way in which they weigh evidence, giving no impression of having a 'pet hypothesis' but simply judging propositions in light of the data. I believe them."

Conversely, the hard sell always puts me in fight mode: "I feel my arm being twisted, and I don't like that. I'm going to search for problems in the story." And you can see evidence of this in my previous review. I went and tracked down all those old references and papers about Doñana because I felt you were trying to sell me a half-baked cake; I don't like paying full fee for a half-baked cake, and I don't know anybody who does (and anyone who does buy such a cake soon learns that it was not well enough baked)

My recommendation on this next revision: Take a big step back, let the manuscript sit for a few days, and go through it and simply try to find all the places where somebody like me might argue that you are overselling -- and cut and trim and deflate any such instances you find. I know that I'll be citing your study, but I'll feel better if you follow not just the letter but the spirit of what I'm trying to communicate about this.
AUTHORS: We restructured the line of arguments as suggested by the Reviewer (lines 143-154).

8. **REVIEWER 1**: *37-39 - This sentence, which at least two reviewers flagged for concern because of "virtually nothing" (and for me, invoking "climate change" as the only relevant driver -- you seem to want to hide the disease element?) -- anyway, the sentence doesn't seem necessary. You could cut it, the segue would be smooth, the focus still general, and you would dive down into IGP starting on Line 54, and you would not have to repeat so many iterations of saying that this or that is unknown: "We know virutally nothing" (12, 37), "precious little knowledge" (60), "virtually no research" (61), "An even less known" (68), "completely uknown" (75).*
AUTHORS: We have deleted the sentence, as suggested.

9. **REVIEWER 1**: *76-78 ... But I don't see the need for this paragraph. This has all been said. There is no need for a synopsis at this stage of the ms -- and no need to say yet again that "we lack key knowledge", a point made at least 6 times already according to my list above.*
AUTHORS: We have deleted the sentence, as suggested.

10. **REVIEWER 1**: *79-116 I would break up this extremely long paragraph.*
AUTHORS: We broke it up into three paragraphs, thank you for pointing this out to us.

11. **REVIEWER 1**: *On the whole, I think that the authors have done their due dilligence in addressing the most salient concerns, and I am convinced by the core of the story. My two main lingering concerns are laid out in great detail above and mainly focus on presentation. The 'competitor retailiation" argument is -- I think we all agree -- not ironclad but at least as consistent with the available evidence as anything the reviewers have raised. The authors can still do more to build the case gently, and therefore more convincingly, but at this point the difference is more stylistic than substantive (although presentation is important!) One can always dream up more data that would be nice to have, but those do not seem strictly essential to support the core conclusions of the work.*
AUTHORS: We heartedly thank the Reviewer for his appreciation of the study.

COMMENTS BY REVIEWER 2:

1. **REVIEWER 2**: *Reviewing the revised manuscript, it is clear the authors have done a fantastic job in revising the manuscript in response to 4 challenging reviews. Many of the points I raised were satisfactorily answered, and I think the reframing of the manuscript that has been completed in response to my (Reviewer 2) and Reviewer 1's comments really improves the work and means that it feels much more rounded as opposed to using the data to tell a specific story. Saying this, I still do not feel that the conclusions can be robustly supported without dietary data (and other contextual data that has not been collected), and for this reason I do not believe that it meets the high bar that is set for publication in Nature. Without these data, it is still not clear what the mechanisms are for these changes and thus the generalisability to other ecosystems is not possible. Although this, in my opinion, precludes the publication in a journal such as Nature, it would make a fantastic contribution to somewhere like Ecology Letters, Global Change Biology or other journals with a strictly ecological focus, where the reader can assess the generalisability themselves and make more informed interpretations. Although I appreciate this might be disappointing for the authors considering the efforts that went into revising the manuscript, this work will not be wasted and I look forward to seeing the work published in the near future.*
AUTHORS: We are glad the Reviewer found our revision work "fantastic" and the manuscript much improved. We have better acknowledged the absence of annual lynx dietary data over the three decades of the study and why we believe it should not affect the results (lines 706-709 and 832-834).

COMMENTS BY REVIEWER 3 (Dr. Briana Abrahms):

1. **REVIEWER 3**: *The authors have fully addressed my main concern. I appreciate them adding data on duck abundance in the revised Figure 3. This now much better completes the picture and supports the authors' interpretation.*
AUTHORS: We thank the Reviewer for her appreciation of the revision.

COMMENTS BY REVIEWER 4:

1. **REVIEWER 4**: *I appreciate the authors' careful consideration of my comments, as well as those of the other reviewers. I am largely satisfied by the revisions. While I remain somewhat skeptical of the competitor removal argument, the authors have made a strong case and have much experience on the ground observing the animals, so I find their explanation sufficient. Figure 3a is a very compelling illustration of the multiple stressor effect. Overall, I think this paper is a great contribution in showing how multiple stressors can rewire ecological interactions.*
AUTHORS: We are very happy that the Reviewer described the manuscript as a "compelling illustration" and "great contribution".

COMMENTS BY REVIEWER 5:

1. **REVIEWER 5**: *This study is a very interesting example on detailed ecological interaction between species and how multi-disciplinary approaches/data can provide additional understanding of observed behavior. I found the reading interesting and authors' arguments about the importance are convincing. However, there are still some improvements and clarifications necessary to make this study sound for Nature publication.*
AUTHORS: We are glad that the Reviewer found the study very interesting.

2. **REVIEWER 5**: *Here are my main critics:*
1) Terminology and expressions are often less scientific and express often subjective "feelings" rather than facts based on data and analyses. I do totally understand that some statements are based on long field experience, which are very important. However, I would suggest to underline those observations/statements more objectively (please compare detailed comments).
AUTHORS: We have done our best to follow the Reviewers suggestions and make our terminology and suggestions less subjective and more "neutral" (see detailed comments and answers below).

3. **REVIEWER 5**: *2) The presentation of spatial data (Extended Data Figure 1 and 2) should be improved. That will make more professional impression and provide additional transparency about used data.*
AUTHORS: We improved Extended Data Figures 1 and 2 as suggested by the Reviewer (see detailed comments and answers below).

5. **REVIEWER 5**: *3) Additional Extended Data Figures showing the distribution of number of monitored kite nests over time would also be of advantage as well as a Figure on available inundation maps and interpolation.*
AUTHORS: We added all the requested information (see newly added Extended Data Figures 5 and 6; see also detailed comments and answers below).

6. **REVIEWER 5**: *Additional Extended Data Figure presenting meteorological and hydrological parameters (e.g. using data from meteorological and hydrological stations from the wetlands measurements network).*
AUTHORS: We have added Extended Data Figure 5 which we believe should clarify the issues raised (see details in Answers 8, 19 and 26).

7. **REVIEWER 5**: *5) In regards to inundation data/information, there are definitely some issues which has to be followed up to avoid criticism and jeopardize the interpretation and outcome of this study.*
AUTHORS: Please see detailed comments and answers below (especially Answers 8, 19 and 26).

8. **REVIEWER 5**: *General comments on surface water maps, their assessment and importance: I totally understand the focus and narrative of this study. <u>The used information about flooding is simply an additional input to quantify whether a year could be called as a drought year or not. Therefore, the focus should not be on the flood mapping approach</u>. Therefore, used inundation maps and provided explanation shall raise as less questions as possible. However, because "drought" seems to be a major factor in the narrative and explanation of observed increase of kite predatory by lynx due to absence of surface water and ducks, I would recommend to provide more transparency. Furthermore, I would like to provide some suggestions and recommendation to the authors, which could be taken into account to improve that part and make it less vulnerable to critic. Since this manuscript is under evaluation for a Nature publication, I would expect that all aspects are at highest scientific and presentation level. <u>First of all, the water mapping methodology which is presented in https://doi.org/10.3390/rs8090775 is very enough and can be considered as accurate surface water classification for the study area. Especially, because studies focusing on a local environment produce mostly more accurate results in comparison to approaches mapping surface water on larger scales (nation, continental, global). This could be a good argument why the authors are using "in house" inundation maps</u>. However, the approach was published ten years ago in 2016. Since then, there was a big progress in satellite remote sensing data, using cloud computing and AI to fully exploit multi-sensor data and new additional open-source datasets (e.g. from Copernicus program Sentinel-2 would be the most relevant dataset). In this way, spatial resolution as well as temporal frequency can be increased tremendously. I believe that both aspects are important for presented study. Therefore, I see a lot of potential critics in that direction as this progress is fully neglected. To avoid this, I recommend that the authors transparently present more details on inundation maps and <u>discuss existing draw backs and why those are probably irrelevant for this study analyses</u>. I highly recommend to involve an experienced remote sensing data scientist and GIS expert to spend time and help improving that part and additional Figures and this way avoid unnecessary and avoidable critics in that direction which could compromise your major interpretation. In the study from 2016 <u>https://doi.org/10.3390/rs8090775</u> the authors of inundation maps already mentioned: "The method is being updated for Landsat 8 OLI and Sentinel-2 images in order to extend the time series of flooding masks and hydroperiods." If that was the case, that should be presented here as well or at least discussed why you do not use additional data from Landsat 8 and Sentinel-2.*
AUTHORS: The Reviewer <u>requests us to use a newer satellite technology or discuss why we did not use it</u>. We thank the Reviewer for the input. We already have two GIS experts as co-authors and we have an in-house GIS laboratory with top-level GIS technicians and

scientists that have been dealing with inundation estimation for the past decades. We consulted again with them in detail about this theme and we also consulted with another separate GIS scientist. We surely understand the potential value of a more recent technology in assessing inundation rates. However, in this specific case, we are very confident that this would not change our classification (and would only generate an unnecessary delay of many months), for the following reasons:

1. the current, Landsat inundation maps have been extensively **ground-truthed** and this is an extremely important process to ensure the reliability of the satellite data. This would have to be repeated with the new technology, which would require months to plan, implement and to cover all conditions. However, as the Reviewer says, this is not a study on GIS methodologies. We use an already well-established and ground-truthed method, we do not see anything wrong with it. The Reviewer also did not report anything wrong with the method and actually praised it.
2. The Copernicus **Sentinel 2 data are available only since 2017** (satellite launched in 2015, very few images in the initial years, real data available only from 2017), i.e. they would not cover all our time series (since 1993). Thus, a portion of the data would still have to require the earlier methodology (implying that it is acceptable anyway), and mixing two technologies/methodologies within the same analysis (with different resolutions, one ground-truthed the other not, etc) does not seem advisable.
3. Sentinel 2 gives maps with 10 m pixels, compared to the 30 m pixels of Landsat. However, **we do not think that this difference in spatial resolution would be so important for the following reason**. In our analysis, the evaluation of inundation rates was assessed over a large area of 684 km^2 covering all the Doñana marshes. We used a large area because we were interested in characterizing conditions that apply to the whole ecosystem, because ducks, kites and other potential prey are highly mobile species and thus we expect them to respond not just to conditions in the lynx study area, but to ecosystem-wide conditions. In re-reading the manuscript, we realized we did not specify such spatial extent of the inundation plot in the previous version. Our mistake and our apologies. We have now added such detail in the text (lines 608-612) and shown the inundation assessment plot in Extended Data Figure 1 (lines 1264-1267). **Because inundation rates were calculated over such a large area, the higher resolution provided by the new technology is very unlikely to change the percentages, or change them in irrelevant manners for the scope of this study. They surely would not change them to the point of converting a drought year into a non-drought year or vice versa**.
4. We consulted with our supporting GIS laboratory and processing the new Sentinel 2 images would take months, they could (possibly) prepare them by October, given their other commitments. **This several months-delay would not be justified by a proportional benefit and we are extra confident that it would end up in exactly the same classification**. We understand that new technologies are attractive, but, in our view, within this context it would be overkill, like “killing a mosquito with a gun”.
5. In Answer 26 below, we show that, **independently of interpolation, data from the real (non-interpolated) inundation masks confirm as drought (inundation troughs) the years that we had classed as drought**. In our view, this should already

clear potential doubts about the reliability of the interpolated estimates. The real Landsat data are already enough to show which were drought years and the Reviewer acknowledges that "in-house" maps are fine and absolutely acceptable. Data on ducks (a obligate wetland-dependent species) confirm these years as drought (Figure 3).

6. Each Sentinel 2 satellite takes an image every 10 days, against the 16 days of Landsat. For both Sentinel and Landsat, there are 2 simultaneously active satellites, implying a frequency of images every 5 and 8 days for Sentinel and Landsat on average, respectively. This is not such a radical difference. In our view, it would not justify months of delay, especially when the real Landsat images already confirm the drought years.
7. While we understand the need for quantitative definitions and technological modernity, we would like to stress that we were (intensively) present and experienced the ecosystem in all the years of study. We witnessed and lived all the droughts and non-droughts and, in our view, direct experience should still be as important as sensors, just as it should be for field observations, e.g. of animal behaviour. For example, if a GPS radio-tag estimated an animal as stationary in a given moment but we see it flying in that moment, who should we believe? (we are citing a real example from our own experience). While we understand and appreciate the importance of technology and remote sensing and benefit enormously by it in our research, we also stress the added value (not the substitution, the added value) of direct observation by field biologists, which is growingly and all too often absent, ignored or treated as of inferior quality. In our case, we are extra confident in our estimates because we know by direct experience that they correspond to reality and have a real biological effect that we directly witnessed and experienced. A new technology would (should) only re-confirm what we saw and experienced. If it did not, we would have to worry about the reliability of the technology. If we were estimating conditions for a continent we have never even visited or for a period in which we were never there, then we would understand the need for technological extra-care to avoid estimation errors and "hallucinations", as sometimes produced by modern AI-based methodologies (which, like any technology, are attractive, but not free from problems).
8. If you want, we can present the above arguments in the text. However, we feel like the portion on inundation estimation is becoming longer and longer (already 3 full pages) and a bit out of proportion with its relative importance for a paper whose prime objective is not the assessment of GIS methodologies or hydrology. Personally, we kindly disagree with the Reviewer's comment: "*I see a lot of potential critics in that direction as this progress is fully neglected*". Why should there be this criticism? Then, why should there not be also criticism for using a technology that has never been ground-truthed? Or why should there not be also criticism for data-processing that is based on AI-techniques that might be proven problematic by future assessments? Where does it end? A famous Latin proverb states: "*Excusatio non petita, accusatio manifesta*" (could be translated as "He who excuses himself without being asked, accuses himself"). Personally, we feel like it is more suspicious to go way over what is necessary to defend a definition before even being criticised, than to simply: (1) state our quantitative definition so that it is repeatable (lines 606-609), (2) explain its

potential limitations (lines 672-683, see Answer 19 below) and (3) show that, even with non-interpolated data, the inundation troughs are there (lines 628-632, see Answer 26 below). There is no reason why the real, ground-truthed data of Landsat should be wrong. However, if the Editor and Reviewer feel it is fundamental, we will add such pre-criticism defence statements, although we feel it would make us look unnecessarily "defensive".

9. **REVIEWER 5**: *L 12: „we know virtually nothing" and L 37-39: "For example, we know virtually nothing about how climate change affects direct interactions among vertebrate top predators, such as intraguild predation".*
This is a crucial statement, which I would have expected to be referenced or underlined with a review. Otherwise, I hope you are aware of the impact such statement can have and can hold your argumentation.
AUTHORS: We did lots of electronic searches but could not find such studies. However, we have distempered the first sentence (lines 12-13). The second sentence has already been deleted in response to Comment 8 by Reviewer #1.

10. **REVIEWER 5**: *L 95 "…over coming years" expression is quite vague. Please use exact future temporal interval you are referring to from cited references. Usually, climate change scenarios provide specific intervals.*
AUTHORS: We have added more specific intervals, as specified by the cited articles (line 91).

11. **REVIEWER 5**: *L 116: It would be interesting to see the distribution of 1662 kite nest over the three decades as a Figure (at least in Extended Data). That would help to get a better feeling on the background data. For example, how many nests were monitored and how many destroyed kite nests were observed for each year? In L 775-776 you do provide averages, but a full transparency in data presentation is necessary.*
AUTHORS: We now provide the requested data in Extended Data Figure 6. Please note that these data would have already been available to the reader, as all data will be placed in a public repository upon publication.

12. **REVIEWER 5**: *L 118: The numbering and mentioning of Figures are not correct. The very first time a Figure is mentioned is Figure 2 in L 118. First time Figure 1 is mentioned is L 683. Please correct accordingly.*
AUTHORS: The missing citation for Figure 1 is in line 76, apologies for the omission.

13. **REVIEWER 5**: *L123-124: "During normal non-drought years…"*
The term "normal" is somehow subjective. Shouldn't it be based on long term climate measurements? I can imagine that climate scientist might ask for long term measurements and statistic on which you consider a year as "normal". E.g. certain annual precipitation +/- StdDev? Certain distribution of monthly precipitation over the year?
AUTHORS: We meant "non-drought", we removed the term "normal".

14. **REVIEWER 5**: *L 239: "…mix of good and bad news" sounds very unscientific to me.*
AUTHORS: We reworded the sentence to a hopefully more "scientific" language (line 233). We'll be happy to follow additional advice if still deemed inappropriate.

15. **REVIEWER 5**: *L 592-593: "…which inundation levels…" The term "level" refers to height of surface water. Please use inundation area instead. Also in L 607.*

AUTHORS: We changed the terminology in both sentences (lines 607 and 625), thank you for pointing this out.

16. **REVIEWER 5**: *L 601: "Global accuracy…" this term is quite unusual and misleading. Usually, in remote sensing the term "overall accuracy" (taken into account over and underestimation) is more common to express what you probably mean by "global accuracy".*

AUTHORS: We changed it to "overall accuracy", as requested.

17. **REVIEWER 5**: *L 602-603: GitHub link does not work. I could not see the protocol, nor the code or flood masks.*

AUTHORS: We have tried the link on several computers and it works. We have pasted it in a different way and it will hopefully work. The protocol is exactly the same as specified in the 2016 paper that the Reviewer mentioned as satisfactory (Comment 8 above). Please note that in any case all shapefile inundation masks will be placed in a public repository upon publication.

18. **REVIEWER 5**: *L 606-608: "We then extracted the minimum inundation level reached on these fixed dates and classified a year as drought when such level was lower than 20 % of the maximum inundation." Here, I would like to raise two issues. First, could you please clarify if 20% of maximum inundation are referring to maximum inundation of the same hydrological year or to long-term maximum inundation observed over the entire study period?.*

AUTHORS: We meant the latter: maximum inundation referring to long-term maximum inundation observed over the entire study period. We have now better specified this in line 624-627. Thank you for pointing out this lack of clarity.

19. **REVIEWER 5**: *Secondly, I would recommend that the authors critically discuss whether they would really like to use the term "drought". If that is the case, more information and more background on drought would be necessary because the term "drought" in scientific sense is complex and defined differently depending on the discipline (e.g. hydrological drought, meteorological drought, agricultural drought). Since the kite predatory seems to depend on absence of surface water causing a reduction of ducks, I personally would feel more comfortable to use the term "surface water availability" instead of drought. Especially, as I assume that the absence of surface water in that wetland is not ONLY affected by changing precipitation regime but is a combination with water management and overuse for agriculture. <u>I believe to cover all that aspects will go beyond the objectives of this study</u>. Although, I believe that this study would highly benefit from a transparent presentation of meteorological and hydrological data. For example, one Figure showing monthly temperature, total precipitation as well as river discharge or river level. That would present climatological variability of study region which is an important background information to understand the climate variability context.*

AUTHORS: We followed the Reviewer's advice and tried to be as transparent as possible about drought, its definition, limitations etc. To this end, we have added the following specific section about this (lines 664-683, see below):

Drought terminology

In this study we hypothesized that lynx could be strongly disadvantaged when low availability of rabbits (the lynx main prey) is compounded with low availability of ducks (the main lynx alternative prey). Because ducks are waterbirds obviously dependent on water, our analyses are centered on the impact of conditions of very low surface water availability, and thus focus strongly on so-called “hydrological drought”. Such water availability is strongly driven but not completely determined by meteorology. For example, cumulative rainfall fallen between 1 October and 1 March explains 74% of the among-year variation in the percentage of the marshes inundated on 1 March ($\beta = 0.49 \pm 0.05$, $t = 9.72$, $P< 0.0001$). Thus, discriminating between hydrological and meteorological drought can be complex and beyond the scope of this paper, whose objective is to examine the wildlife consequences, not the causes of drought. For simplicity, here we use the generic term “drought” to indicate hydrological drought, estimated as very low surface water availability (i.e. years in which more than 80% of the wetlands are dry). In our field experience, these are years characterized by high aridity and very low prey availability for all predators in the ecosystem. We acknowledge that, as with any extreme event, other classifications could be possible. However, models based on alternative continuous estimates that we could think of (e.g. inundated area on key dates, cumulative winter rainfall) gave the same conceptual results (e.g. highly significant interactions between these estimates and rabbit abundance), but much worse model fit (> 10 AICc-units increments). Throughout the paper, the generic term “drought” could be used interchangeably with “hydrological drought” or “very low surface water availability”.

In the current version of the manuscript, we have better acknowledged (lines 664-683) that water availability is linked (substantially, but not perfectly, as any ecological process) to weather conditions (e.g. cumulative rainfall fallen between 1 October and 1 March explains 74 % of the among-year variation in the percentage of the marshes inundated on 1 March). Thus, the division between “meteorological drought” or “hydrological drought” is not so clear-cut and the need to decide whether to focus on “meteorological drought” or “hydrological drought” maybe not be so important? To us, summarizing and simplifying, the involved processes are:

1. meteorology → 2. inundation rates → 3. predator interactions

Our study examines the consequences of 2 for 3, not the effect of 1 on 2, which is the expertise of meteorologists and hydrologists. In our view, the exact mechanism of causation of drought and low water availability is not the scope of this paper. The paper is about the wildlife consequences of drought, not an analysis of the causes of drought. For lynx, ducks, kites and the processes which are the focus of this study, it is better to directly focus on extremely low water availability as a main manifestation of drought than on meteorological relations, as this will be directly more relevant for the target processes of top predator interactions.

In our view, and already in the previous version of the manuscript, we gave a quantitative definition of what in our field experience is a low-enough inundation (80% of the wetlands are dry-hard clay instead of water with its associated wetland vegetation and rich life) that duck availability is low and the ecosystem as a whole yields low prey availability. Locally, these years were unanimously identified as “sequías” (drought) by local field workers, park wardens, farmers, villagers etc. Of course, other definitions or estimations of drought could be possible, but this regards any extreme event and a large share of ecological processes. For example, “fitness” is estimated in the ecological literature as long-term survival, longevity, number of offspring generated through life, number of recruits generated through life, genes passed over to the next generation, etc. These are all legitimate components of fitness, but we do not see authors “battling” to defend why the estimate that they employed is valid as if it is going to be criticized the next day.

In whatever case, in the current version of the manuscript, we have added a specific section (lines 664-683) where we:

- give our definition of drought and the rationale for its use;
- acknowledge the limits of this definition and estimation.

We believe and hope that this will suffice. In any case, readers will have the shapefiles of inundation. The analyses can be easily redone, but we feel very confident and comfortable that the results are robust. For example, as we already stated in the previous cover letter, we have redone the analyses by using alternative continuous estimates (e.g. inundated areas on various key dates, cumulative autumn-winter rainfall). Compared to our currently-presented model based on dichotomous drought, all the AICc of these "continuous variables" models are much higher (= worse-fitting) by 10.4 - 79.0 AICc-units. Furthermore, in all these models with continuous indicators, the interaction between rabbits and each continuous indicator continues to be always highly significant (i.e. the results are unchanged and confirmed anyway). Similarly, we have rerun the models by leaving one year out each time and the results are always the same (highly significant interactions between rabbit abundance and drought). We have also redone the analyses by (wrongly) classifying each key year of high predation as a non-drought year instead of a drought year and in that case the year is systematically flagged as an extreme statistical outlier (by whatever diagnostic: Cook's distances, leverage values, deviance residuals; e.g. extremely high Cook's distances ranging from 4-12). As even further conceptual support, the models of Fig. 2 explain 88% of the variance (which we would not expect if the underlying ecological variables were misidentified) and even the troughs in the abundance of ducks (wetland birds obligatorily dependent on water) confirm that the years identified as drought were effectively drought (5- to 8-fold crashes in duck abundance between consecutive drought and non-drought years or vice versa: see Figure 3).

If the editor and reviewer see it as fundamental, we could try to change the text to "surface water availability", but we think it would be a mistake and weaken the efficacy of the text by making arguments fuzzier. To us, the average reader would think we are basically talking about drought without calling it drought.

20. **REVIEWER 5**: *L 627: "15 May" is that correct? Before you used 15 March. If 15 May is correct, I wonder why you use this date to estimate spring precipitation? Its neither meteorological nor astronomical spring period. Could you please explain?*

AUTHORS: Yes it is correct: we wanted to characterize years in which unusual, late rainfall caused a sudden, late inundation. In the current version, we have changed the term "extreme spring rainfall" which probably generated confusion to "extreme, late rainfall" that better captures the concept we wanted to convey.

210. **REVIEWER 5**: *L 629-630: "So defined, anomalous years coincided with the drought years listed above, but with the addition of 2011, given its copious spring rains" It is unclear to me why you add 2011 if there was more rain than usual? Or is it a translation mistake?*

AUTHORS: We characterized anomalous years as those with drought OR with extreme, late rains (OR with extreme temperatures). 2011 was not a drought year, but it had extreme, late rains. We did not want to flag extreme rains only in one direction, and so we classed as "anomalous" any year with late rains (drought or not), so as to stay conservative.

22. **REVIEWER 5**: *L 776: "(N = 64.9 checked nests/year on average, range = 20-103 and 52-86 after 2013)" –> missing the range before 2013.*

AUTHORS: It was the first of the two reported ranges, but it was effectively not so clear. Our apologies. We have now changed it to: "N = 64.9 checked nests/year on average, range = 20-103 before 2013 and 52-86 after 2013" (line 822-823).

23. **REVIEWER 5**: *L 777: "Data on rabbit density were only available since 2005. For the preceding years, rabbit density was back-estimated by a regression…" This HAS to be also indicated in Figure 3!*

AUTHORS: We better specified this in the legend (lines 1153-1154), thank you for pointing this out.

24. **REVIEWER 5**: *L 949, L 1227, L 552: should be MSS (Multispectral Scanner System) not only "MS".*

AUTHORS: Please see next answer.

25. **REVIEWER 5**: *L 599-600: Landsat MSS has a spatial resolution of 60m!*

AUTHORS: Some clarification is needed: the sensor MSS of Landsat with a resolution of 60 m was used until 1984. Subsequent sensors had a resolution of 30 m (e.g. https://science.nasa.gov/mission/landsat/spectral-bands-and-applications/#mss). In this analysis, we use only data from 1993, thus the 30 m resolution is correct. We mistakenly included the MSS sensor in our previous text, which we have now deleted. Apologies for the confusion generated. We fixed the text (line 1296).

26. **REVIEWER 5**: *L 604-606: "To make inundation rates comparable across years, we linearly interpolated consecutive inundation masks so as to estimate the inundated area for the fixed dates of 15 February, 1 March, 15 March and 30 March every year." This is a crucial moment which you need to prepare as transparent as possible. As you mentioned, usability of Landsat is depending on cloud coverage. Landsat frequency is 16 days for used data. The results of linear interpolation will highly depend on "when" and how many scenes were available. According to Figure 5 from https://doi.org/10.3390/rs8090775 the number of flood masks can be less than 10/year. A figure spanning the three decades showing the time series of derived inundation area and interpolation curve would be very helpful to see that the used flooding data does not introduce some bias because of scarcity of Landsat scenes during critical season. Additionally, a plot of river level or discharge which hopefully will show similar temporal variability with your interpolation would strengthen your argumentation and show that the approach does not neglect surface water variability.*

AUTHORS: We believe that there is a quick and easier way to avoid the potential "interpolation doubts" cited by the Reviewer by directly showing the real inundation rates in the original Landsat images. These data do not suffer from potential artefacts of interpolation techniques. In the current version, we have added such graph as Extended Data Figure 5 (lines 628-632). The red points in the figure quickly highlight the years in which the percentage inundation went below 20% (i.e. ≥ 80% of the marshes were dry) in the key window of February-March and confirm the classification based on interpolation. The text commenting this new addition and confirmation can be found in lines 628-632. Please note how even the data on duck abundance (obligate wetland dependent species) shown in Figure 3 confirm the same years as droughts (5- to 8-fold crashes in duck abundance between consecutive drought and non-drought years, please see Figure 3b). It's unconceivable for the Doñana ecosystem that ducks would settle in high numbers during a drought, or settle in low numbers during a high-inundation year. In this sense, "ducks don't lie".

Regarding the interpolation, a few quick comments, just in case. In Extended Data Figure 5 we have also added the percentage inundated area on the dates just before and just after the

temporal window of February-March, so the readers can get a quick idea of the relevant image-frequency for each year. We did not add further dates or the figure become visually clogged, unreadable and confusing. In general, cloud-free Landsat images tend to be less frequent: (1) in the early years of the study and (2) in high-inundation years, because these are more rainy and thus more frequently cloudy, resulting in lower frequency of cloudless Landsat images. Regarding point (1), rabbits were always abundant in the early years of the study (pre-2013), implying that lynx did not "need" ducks and was thus "buffered" from drought, i.e. the analysis is basically unaffected by the drought classification. In those years, even if we had misclassified drought or non-drought years (which we did not), the models and their ecological results would have been unaffected (e.g. the R^2 of Figure 1 would remain the same). 1996 is the only early year in which rabbit abundance was low in the early part of the study, but flooding was extremely massive already in February, to the point that circulating around the park even with 4x4 cars was extremely difficult (at the time it was the highest inundation since the earliest record of 1975). There is no doubt that 1996 was not a drought. Regarding point (2), lower frequency of Landsat images on rainy-cloudy years implies that the years where the interpolation could be less precise are not the drought years, but those with already abundant or very abundant inundation, i.e. those that are very far from being wrongly assigned as drought (e.g. 1996 itself). Thus, overall, the current classification is conservative and unlikely to suffer from interpolation problems, besides the fact that the real (non-interpolated) Landsat images are already enough to confirm the drought years, which are also confirmed by ducks' abundance. Should there be any doubts about any specific year, we would be confident and happy to clarify them.
Finally, we do not have data on river level/discharge.

27. **REVIEWER 5**: *L 1063: Figure 3: Why is there a drop of rabbits in early nighties?*
AUTHORS: The reasons were not investigated at the time and so are unfortunately completely unknown.

28. **REVIEWER 5**: *Ext. Fig. 1 and Ext. Fig. 2: Basic cartographic information is missing: scale bar, coordinates, what is used as background image, which month or year are the flood masks from?.*
AUTHORS: We added all the requested information to the figures and legends, thank you for pointing this out.

We thank the Editor and Reviewers for all their additional work on our manuscript, we hope to have clarified and improved all the issues raised. We believe that, thanks to all the suggestions, the current version of the manuscript has improved in clarity, completeness and solidity. It would an honour to publish again in Nature.

Best regards,
Fabrizio Sergio & co-authors

Dear Dr. Armstrong,

Thank you for the additional comments by yourself and Reviewer #5 on our manuscript, now newly titled "Drought and disease trigger novel interactions among top predators", as suggested by yourself.

Following your requests, in this new version of the manuscript, we have:

1. changed the title in the manner suggested by the Editor;
2. added a caveat-sentence on the availability of additional inundation maps (lines 482-484), as requested by the Editor;
3. moved the acknowledgement of funding to an ad hoc section titled "Funding", as requested.
4. deleted the Extended Data Figures 7 and 8, as these were illustration-schemes of conceptual results generated by AI, which are not currently allowed. This follows an email request of information about this to Dr. Samuel J. Cockman;
5. submitted all figures and Extended Data material as separate files according to Nature instructions for authors;
6. provided the manuscript in .docx format;
7. provided the Third Party Table and all the other requested checklists.

Number of words in current manuscript = 3382.

[redacted]

[redacted]

COMMENTS BY REVIEWER 5 (Dr. Igor Klein):

1. **REVIEWER 5**: *Dear Dr Sergio and co-authors, Thank you for your detailed replies and revisions. Here are my comments for this round of review. In general, that version is much improved! My main concerns about terminology and with few statements "overselling" were taken care off. Especially thanks to reviewer#1's comments and very well explained concerns and authors revisions in this versi*on.

AUTHORS: We are glad that our revision work and responses were considered satisfactory.

2. **REVIEWER 5**: *Comment 8. REVIEWER 5 from last round:*
Thank you for detailed explanation and arguments. I see your point and I can understand where you coming from with your explanations. I personally am okay with that. I also agree that spatial resolution of 10 m vs 30 m would not introduce much difference in that case. However, temporal resolution is much more important for your case study and how many cloud free images you have over your study period. Anyways, I cannot judge whether the inclusion of denser time series would in fact have increase the number of cloud-free images and thus the number of water mask contributing to more stable and robust results. At the end, it is your decision and judgement based on your field experience and observation of flood dynamics and what additional mapping efforts are practicable and which additional outcome of water mask time series might contribute for any kind of improvement. My goal and responsibility as a reviewer were to underline the existing opportunities and I also did that because it was mentioned in the water mapping study from 2016 that the "in-house" methods would be modified for Landsat-8/Sentinel-2.
Best wished and good luck with upcoming rounds,
Igor Klein
AUTHORS: We heartedly thank Dr. Klein for his appreciation and for his current and previous input and opinion. We are confident that the data shown in Extended Data Figure 5 adequately portray the inundation dynamics of the ecosystem, as further reinforced and confirmed by duck data from Fig. 3b.

We thank the Editor and Reviewer for all their additional work aimed at improving our manuscript.

Best regards,

Fabrizio Sergio & co-authors
